# Synthesis and Antiplasmodial Activity of Bisindolylcyclobutenediones

**DOI:** 10.3390/molecules26164739

**Published:** 2021-08-05

**Authors:** Duc Hoàng Lande, Abed Nasereddin, Arne Alder, Tim W. Gilberger, Ron Dzikowski, Johann Grünefeld, Conrad Kunick

**Affiliations:** 1 Institut für Medizinische und Pharmazeutische Chemie, Technische Universität Braunschweig, Beethoven straße 55, 38106 Braunschweig, Germany; h.lande@icloud.com (D.H.L.); j.gruenefeld@tu-bs.de (J.G.); 2 Department of Microbiology and Molecular Genetics, IMRIC, The Kuvin Center for the Study of Infectious and Tropical Diseases, The Hebrew University-Hadassah Medical School, Jerusalem 91120, Israel; abedn@ekmd.huji.ac.il (A.N.); rond@ekmd.huji.ac.il (R.D.); 3 Genomics Applications Laboratory, Core Research Facility, Faculty of Medicine, The Hebrew University-Hadassah Medical School, Jerusalem 91120, Israel; 4 Centre for Structural Systems Biology, 22607 Hamburg, Germany; alder@bnitm.de (A.A.); gilberger@bnitm.de (T.W.G.); 5 Bernhard Nocht Institute for Tropical Medicine, 20359 Hamburg, Germany; 6 Department of Biology, University of Hamburg, 20146 Hamburg, Germany; 7 Zentrum für Pharmaverfahrenstechnik (PVZ), Technische Universität Braunschweig, Franz-Liszt-Straße 35A, 38106 Braunschweig, Germany

**Keywords:** bisindolylmaleimide, cyclobutenedione, drug design, drug screening, Friedel-Crafts reaction, glycogen synthase kinase-3, indole, malaria, molecular docking, plasmodium

## Abstract

Malaria is one of the most dangerous infectious diseases. Because the causative *Plasmodium* parasites have developed resistances against virtually all established antimalarial drugs, novel antiplasmodial agents are required. In order to target plasmodial kinases, novel *N*-unsubstituted bisindolylcyclobutenediones were designed as analogs to the kinase inhibitory bisindolylmaleimides. Molecular docking experiments produced favorable poses of the unsubstituted bisindolylcyclobutenedione in the ATP binding pocket of various plasmodial protein kinases. The synthesis of the title compounds was accomplished by sequential Friedel-Crafts acylation procedures. In vitro screening of the new compounds against transgenic NF54-*luc P. falciparum* parasites revealed a set of derivatives with submicromolar activity, of which some displayed a reasonable selectivity profile against a human cell line. Although the molecular docking studies suggested the plasmodial protein kinase *Pf*GSK-3 as the putative biological target, the title compounds failed to inhibit the isolated enzyme in vitro. As selective submicromolar antiplasmodial agents, the *N*-unsubstituted bisindolylcyclobutenediones are promising starting structures in the search for antimalarial drugs, albeit for a rational development, the biological target addressed by these compounds has yet to be identified.

## 1. Introduction

Occurring mainly in tropical and subtropical areas, malaria remains one of the most threatening infectious diseases on earth. Transmitted by the bite of an infected female *Anopheles* mosquito, malaria is caused by pathogenic protozoa of the genus *Plasmodium*, wherein the species *P. falciparum* is the most dangerous pathogen. It is responsible for *malaria tropica*, the most severe form of the disease, which can be fatal if left untreated. It is estimated that there were 229 million malaria cases in 2019, of which about 409,000 were fatal, most of them in sub-Saharan Africa [1]. Besides its impact on individual and public health, malaria causes enormous economic damage and is linked to the endemic poverty of the affected countries [2].

Plasmodia are intracellular protozoa whose complex life cycles are divided into two phases: asexual reproduction that occurs in vertebrates and sexual reproduction in the vector, the *Anopheles* mosquito [3]. As vector control and prevention measures, the World Health Organization promotes insecticide-treated bednets and the use of insecticides for regular indoor spraying. Implementation of these procedures has resulted in a significant decrease in infection number in the recent two decades [4]. Unfortunately, this progress has come to a standstill in recent years [1,5,6]. To treat severe malaria in countries with high resistance rates to conventional malaria drugs, the WHO recommends artemisinin-based combination therapies as the medication of choice. Increasing resistance to all classes of antimalarial agents, including artemisinin derivatives, poses a threat to endemic regions. A reason for the emerging resistance is the improper use of antimalarial drugs, which exerts selection pressure on the parasites [4,7,8,9,10].

For the treatment of infections with resistant parasites, new drugs are needed. To minimize the risk of cross-resistance, these new medications should be distinguished from established chemotherapeutics by mode of action and chemical structure. In this regard, it would be favorable to address alternative biological targets in the parasites. The plasmodial kinome offers such potential alternative targets [11,12,13]. In the genome of *P. falciparum* at least 65 kinases can be identified that belong to established eukaryotic protein kinase groups, many of which are similar to human protein kinases [12,14]. Nevertheless, significant phylogenetic divergence between the mammalian and the plasmodium kinome results in mechanistic and structural differences of the enzymes which may be exploited for drug design strategies. Basic reverse genetics experiments and phosphoproteomic analyses resulted in a list of plasmodial kinases that are essential either in erythrocytic schizogonia or in the sexual developmental stage of parasites [14]. Thus, plasmodial protein kinases have been suggested as drug targets [11,12,13]. However, while the inhibitor of the plasmodial phosphatidylinositol 4-kinase (*Pf*P4K) MMV390048 is currently studied in clinical trials [15,16], compounds acting on protein kinases of the parasites have not yet reached this status [6].

**Figure 1:**
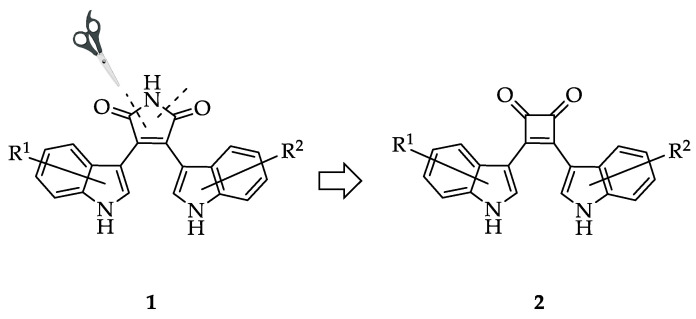
Design of 3,4-bis(indol-3-yl)cyclobut-3-ene-1,2-diones **2** by formally cutting out the imide nitrogen of bisindolylmaleimides (BIMs, **1**). R^1^ and R^2^ indicate substituents attached to positions 1, 2, 4, 5, 6, or 7 of the indole ring system.

Bisindolylmaleimides (BIMs, **1**) are an established protein kinase inhibitor class [17,18], represented by prominent examples such as GF 109203X [19], enzastaurin [20] and ruboxistaurin [21]. To identify protein kinase inhibitors based on new chemical matter, we designed the compound class of 3,4-bis(indol-3-yl)cyclobut-3-ene-1,2-diones **2** by replacing the imide ring of BIMs with a cyclobut-3-ene-1,2-dione element. The core structure of these molecules is derived from squaric acid (Figure 1). While a few examples of squaric acid diamides with antiplasmodial or other biological activities are known [22,23,24,25], up to now only a single 3,4-bis(indol-3-yl)cyclobut-3-ene-1,2-dione was prepared in a meager yield (0.67%) [26], and biological activity of this congener was not reported.

Before synthesis methods for the production of bisindolylcyclobutenediones **2** were developed, docking studies were carried out to virtually fit the putative new inhibitors into the ATP binding pockets of exemplary plasmodial protein kinases. Since these in silico analyses generated promising results, the compounds **2** were synthesized and tested against erythrocytic stages of *P. falciparum* parasites. Indeed, some congeners proved to be active in submicromolar concentrations.

Based on the docking studies, the protein kinase *Pf*GSK-3 [27,28,29,30] was assumed as a putative target structure for the antiplasmodial compounds within the parasites. However, in vitro enzyme inhibition assays refuted this assumption. The relevant biological target structure for bisindolylcyclobutenediones in plasmodia is therefore still unknown and has yet to be elucidated.

## 2. Results

### 2.1. Molecular Docking

The unsubstituted 3,4-bis(indol-3-yl)cyclobut-3-ene-1,2-dione **2a** was docked into known structures of plasmodial protein kinases using the GOLD program [31], whereby no constraints were defined and the presence or absence of water molecules at suitable positions of the ATP binding pocket was permitted by the application of the “toggle” function. The plasmodial protein kinase structures were downloaded from the protein data bank [32], if available. The protein kinases *Pf*GSK-3 (homology model [33], based on template 1J1B [34]), *Pf*map-2 (PDB: 3NIE), *Pf*PK5 (PDB: 1V0P [35]), *Pf*CDPK2 (PDB: 4MVF) *Pf*lammer (PDB: 3LLT) and *Pf*PK7 (PDB: 2PML) were used as templates. The obtained docking results were assessed by visual inspection, and the most probable orientations are shown below. The structure of **2a** was modified taking into account the suggested orientations to optimize the bioactivity. Furthermore, it was intended to generate clues regarding the relevant biological targets by comparing the structure-activity relationships in the compound class with the orientations in the six plasmodial protein kinases suggested by the docking analyses [36].

#### 2.1.1. *Pf*GSK-3

**Figure 2:**
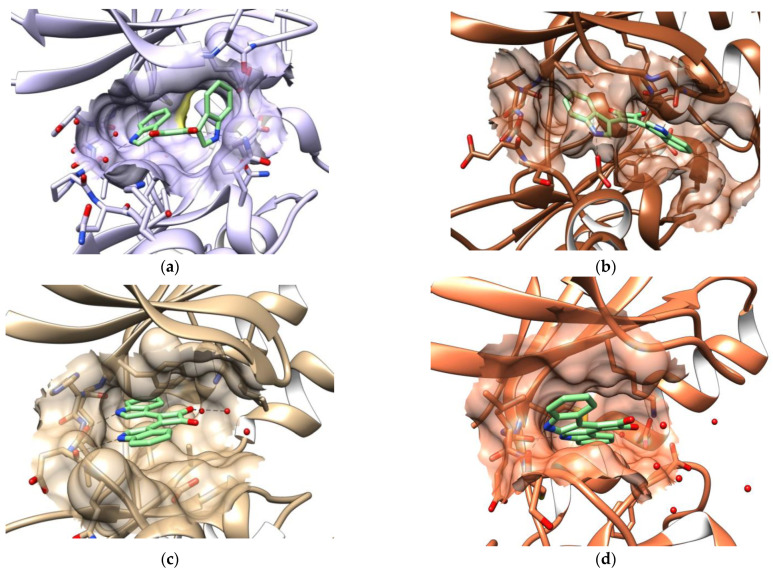

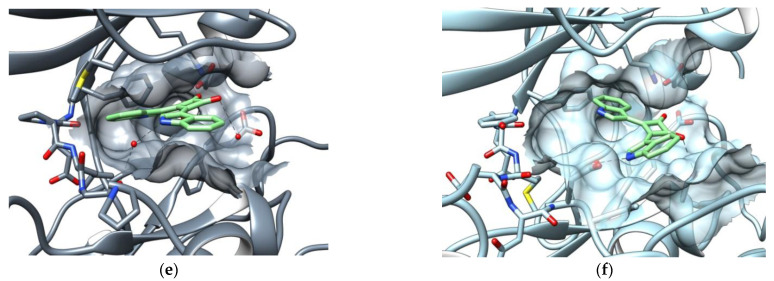
Poses of **2a** in the ATP binding site of six plasmodial protein kinases resulting from molecular docking. (**a**) *Pf*GSK-3 homology model based on template 1J1B. (**b**) *Pf*map-2 (PDB: 3NIE). (**c**) *Pf*PK5 (PDB: 1V0P). (**d**) *Pf*CDPK2 (PDB: 4MVF). (**e**) *Pf*lammer (PDB: 3LLT). (**f**) *Pf*PK7 (PDB: 2PML). Dashed lines indicate suggested hydrogen bonds.

Since no experimentally determined spatial structure of *Pf*GSK-3 exists at present, a homology model of the *Pf*GSK-3 was selected for the investigations described here, which had been created using a crystal structure of human GSK-3 [33]. In the depicted pose, an indole ring of **2a** occupies the adenine region and interacts with the hinge region via a hydrogen bond. The other indole ring is orthogonally arranged and forms a hydrogen bridge to the protein. Furthermore, a keto group of the ligand acts as an H-bond acceptor and interacts with the side chain of Lys166. The cyclobutenedione ring is positioned in the sugar region. In this docking pose, the ligand fills the binding pocket very well (Figure 2a).

#### 2.1.2. *Pf*map-2

In the docking experiment with **2a** in *Pf*map-2 (plasmodial mitogen-activated protein kinase 2; PDB: 3NIE), only one orientation of the molecule in the ATP binding pocket was identified, in which, however, no hydrogen bonds are formed to the hinge region (Figure 2b). An indole ring occupies parts of the ribose and adenine areas and forms a hydrogen bond to an aspartyl residue in the front region of the binding pocket. The second indole element is located in the front part of the binding pocket. One of the carbonyl groups forms a hydrogen bridge to catalytic lysine. Overall, this predicted pose appears unfavorable for a tight binding of the ligand.

#### 2.1.3. *Pf*PK5 

Molecular docking of **2a** in *Pf*PK5 (PDB: 1V0P, [35]) provided a very realistic pose in which the three ring systems are arranged almost in plane. Both indole-NH groups form hydrogen bonds with the hinge region. In addition, there are interactions of a water molecule with a carbonyl group of the cyclobutenedione element (Figure 2c).

#### 2.1.4. *Pf*CDPK2

The predicted pose of **2a** in the plasmodial kinase *Pf*CDPK2 (PDB: 4MVF) shown in Figure 2d is very similar to the suggested orientation in *Pf*PK5. Also in this case, the linked ring systems are arranged planar in the area of the adenine binding site. While the indole-NH groups point in the direction of the hinge region and form hydrogen bonds there, the carbonyl groups of the ligand in this pose are not involved in direct bonds to the protein.

#### 2.1.5. *Pf*lammer

The docking of **2a** in the plasmodial kinase *Pf*lammer (PDB: 3LLT) resulted in an orientation of the cyclobutenedione ring towards the Lys-Glu salt bridge. One of the two carbonyl groups forms a hydrogen bridge to the conserved lysine in the rear area of the binding pocket. While direct bonds to the hinge region are lacking, one of the indole nitrogen atoms forms an interaction with this region mediated by a water molecule (Figure 2e).

#### 2.1.6. *Pf*PK7

The docking orientation of **2a** in *Pf*PK7 (2PML) shows that an indole ring is planar to the cyclobutenedione element and lies deep in the binding pocket. The other indole ring is inclined by about 45° relative to the cyclobutenedione element and is placed in the sugar-binding region of the active center. **2a** forms two hydrogen bonds in this pose, but these are not directed towards the hinge region. The ligand does occupy the adenine binding site (Figure 2f).

### 2.2. Design Considerations

The structure of **2a** was modified, taking into account the docking experiments’ orientations to optimize the bioactivity. These structure variations were also intended to generate clues regarding the relevant biological targets from the observed structure-activity relationships. In this context, symmetrical and nonsymmetrical analogs of **2a** were designed by a formal introduction of substituents in both indole units. The known bisindolylmaleimide **8** [37] was included in the study for comparison.

### 2.3. Chemistry

**Scheme 1:**
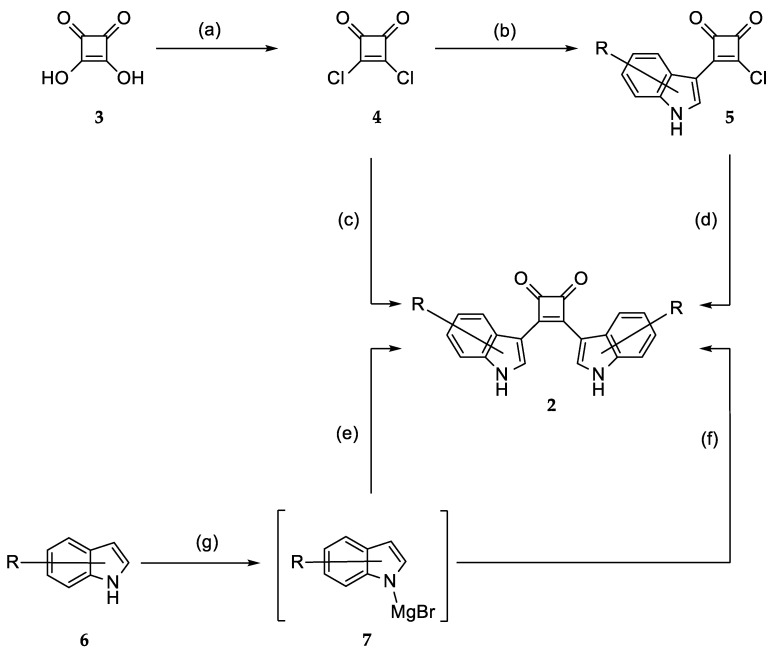
Syntheses of bisindolylcyclobutenediones **2a**–**2ar**. (a) C_2_Cl_2_O_2_, CCl_4_, DMF, 50 °C, 4 h (79%); (b) indole (1 eq), Et_2_O, 0 °C→rt, ca. 24 h; (c) 1. indole (2 eq), CH_2_Cl_2_, AlCl_3_, 0 °C→rt, 20–144 h; 2. HCl/H_2_O (<12%); (d) 1. indole (1 eq), CH_2_Cl_2_, AlCl_3_, 0 °C→rt, 10–65 h; 2. HCl/H_2_O (5–70%); (e) 1. **4**, toluene/Et_2_O/THF (5:1:1), N_2_, reflux, 4–6 h; 2. H_2_O (7–37%); (f) 1. **5**, toluene/Et_2_O/THF (5:1:1), N_2_, reflux, 4–6 h; 2. H_2_O (7–66%); (g) EtMgBr, N_2_, Et_2_O, toluene, 60 °C, 1 h. For substituents R refer to Table 1.

**Scheme 2:**
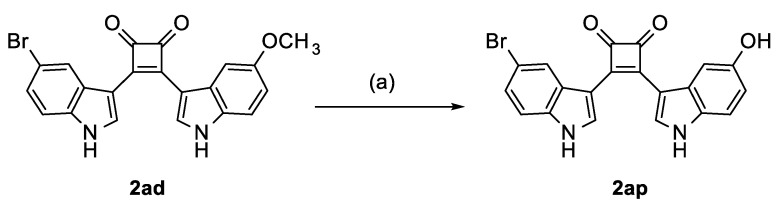
Synthesis of bisindolylcyclobutenedione **2ap**. *Reagents and Conditions*: (a) 1. BBr_3_ (8–16 eq), CH_2_Cl_2_, RT, 15 h, 2. H_2_O, RT, 1 h.

The synthesis of the bisindolylcyclobutenediones **2a**–**2ar** was based on 3,4-dichlorocyclobut-3-ene-1,2-dione (squaric acid dichloride, **4**), which is readily obtained from squaric acid (**3**) and oxalyl chloride [38]. It has been reported that 3,4-bisarylcyclobutene-1,2-diones can be prepared by a reaction of **4** with two equivalents of an aromatic compound in the presence of aluminum chloride [39]. However, this method is only suitable for symmetrical compounds and resulted in very low yields of product **2a**. Better results were gained by a sequential process. For this method, the dichloride **4** was first reacted with one indole equivalent [40]. Subsequent reaction with a second equivalent of the same or another indole resulted in better yields and enabled the production of asymmetric compounds with two differently substituted indole units. A further improvement was achieved by strengthening the nucleophilicity of the indoles **6** by conversion into magnesium derivatives **7**, a procedure that has already been described for the production of asymmetric bisindolylmaleimides [41]. The indole magnesium intermediates **7** were then transformed into the title compounds by reaction either with squaric acid dichloride **4** or monoarylated intermediate **5** (Scheme 1). The hydroxylated compound **2ap** was produced by ether cleavage with boron tribromide from the methoxy-substituted analog **2ad** (Scheme 2).

### 2.4. Biological Evaluation

All synthesized new bisindolylcyclobutenediones **2a**–**2ar** and the related bisindolylmaleimide **8 [37]** were screened for inhibition of erythrocytic stages of *Plasmodium falciparum* employing a bioluminescence-based viability assay on transgenic parasites that constitutively express luciferase (*Pf*NF54-*luc*) [30]. Initially, the compounds were evaluated in single-dose test runs (3 µM). If >20% viability inhibition was observed, dose-response curves were generated and IC_50_ values were calculated (Table 1). Depending on the substitution pattern on the indole subunits, several congeners proved to be rather potent, exhibiting considerable inhibition in single-digit micromolar or in submicromolar concentrations. The bisindolylmaleimide **8** [37] inhibited the parasites in a single-digit micromolar concentration, similar to many of the bisindolylcyclobutenediones in the study. Because the bisindolylcyclobutenediones **2** display a higher degree of structural novelty, only this compound class was further pursued for its antiplasmodial activity. Some among the active compounds displayed substituents at the indole scaffolds in 1, 2, 5- or 7-position, respectively. These structural features were considered unfavorable for orientations as illustrated in Figure 2 with **2a** and *Pf*map-2, *Pf*PK5, *Pf*CDPK2, *Pf*lammer, and *Pf*PK7, respectively. However, a structure generated for **2a** in *Pf*GSK-3 in which the indole units are oriented perpendicular to each other (Figure 2a) was considered compatible with the indicated substitution pattern. Consequently, the potent antiplasmodial derivatives **2k**, **2ad,** and **2ae** were tested in a kinase Glo assay [42] for inhibition of recombinant *Pf*GSK-3. However, these exemplary structures were inactive up to a concentration of 10 µM, so the biological target responsible for the antiplasmodial activity of the title compounds has not yet been identified. For the compounds with particularly potent antiplasmodial activity, cytotoxicity was also determined using the human cell line THP-1 to assess selectivity with respect to parasite cells (Table 2). In these studies, representative **2ai** was found to be particularly beneficial with a selectivity index of 259.

## 3. Discussion

In view of the increasingly critical resistance situation in drug therapy for malaria, the development of additional active agents is urgently required. These agents should be structurally distinct from established antiplasmodial drugs and directed against previously unaddressed biological targets. Representing a well-studied enzyme class, the plasmodial protein kinases comprise entities which are essential for parasite development and replication. Given the high number of protein kinase inhibitors approved as anticancer drugs, protein kinases are generally accepted as “druggable”. Hitherto, the compound class of 3,4-bis(indol-3-yl)cyclobut-3-ene-1,2-diones **2** has been neglected in the search for biologically active compounds. This is surprising since the compounds show structural similarity to bisindolylmaleimides **1**, which are established protein kinase inhibitors. Therefore, we investigated whether the unsubstituted prototype **2a** could be fitted into the ATP-binding pockets of plasmodial protein kinases by a docking routine. In these experiments, structures of plasmodial kinases from the Protein Data Bank were used as templates. A commonly observed binding motif generated by docking showed in some cases (*Pf*GSK-3, *Pf*Pk5, *Pf*CDPK2) hydrogen bonding between the hinge region of the kinase and the potential ligands, with at least one of the indole nitrogen atoms acting as a hydrogen bond donor (Figure 2). A literature survey revealed that bisindolylcyclobutenediones without substituents on one or both nitrogen atoms were previously unknown. A series of corresponding compounds was therefore synthesized. Sequential Friedel-Crafts reactions of squaric acid dichloride (**4**) with two differently substituted indole systems provided access to unsymmetrically substituted title compounds. Since the biological target in plasmodia was not a foregone conclusion, the new compounds were tested in phenotypic screening. Phenotypic screenings have the advantage over target-oriented methods in that the antiparasitic effect can be achieved by attacking different, even initially unknown, target structures. In addition, positive results from phenotypic screenings ensure that the active ingredients can overcome the cell membrane of the parasites and remain there intracellularly long enough to exert their effect [43,44,45,46,47,48,49].

Testing of the unsubstituted prototype **2a** in a viability assay on genetically modified parasites constitutively expressing luciferase resulted in inhibition of the parasites in the single-digit micromolar concentration range. Although bisindolylmaleimide **8 [37]** inhibited the parasites at comparable concentrations, we decided rather to further investigate the structurally novel 3,4-bis(indol-3-yl)cyclobut-3-ene-1,2-diones **2**. Substitution at the 5-position on one of the indole rings of **2a** with halogen or cyano substituents led to slightly improved results (**2b**, **2c**, **2f**). The introduction of a 5-iodo substituent even shifted the antiplasmodial activity into the submicromolar range. In contrast, a symmetric 5,5-disubstitution with halogen, cyano, or methoxy substituents resulted in lower inhibitory activities (**2n**, **2o**, **2p**, **2aq**).

While methyl substitution at both indole nitrogen atoms (compound **2i**) significantly decreased the antiplasmodial activity, single *N*-methylation gave improved results in some cases (**2q**, **2u**, **2y**). The otherwise unsubstituted representative **2k** with a basic side chain on one of the two indole nitrogen atoms also showed a solid antiplasmodial efficacy. In contrast, diminished activity was observed when *N*-methylation at one of the indole nitrogen atoms was combined with a substitution at the 5-position of the other indole ring (**2t**, **2af**, **2ag**). While most representatives with 2-substituted indole rings were hardly active (**2g**, **2l**, **2s**, **2z**, **2aa**), some congeners with 2-phenylindole substitution still showed activity (**2h**, **2m**, **2u**). A benzyloxy substituent on one of the two indole rings prevented potent antiplasmodial activity of the corresponding derivatives **2e**, **2ar**.

The influence of a methoxy substituent in the 5-position at one of the two indole elements connected with the substitution at the other indole ring was striking but inconsistent. While the bromo substitution at the second indole ring in 4- and 6-position reduced the antiparasitic effect (**2am**, **2an**), the 5- or 7-bromo isomers exhibited strong antiplasmodial activity in the submicromolar concentration range (**2ad**, **2aj**). Similarly, the 5-methoxy-substituted derivatives with chloro (**2ac**, **2ai**) or iodo substitution (**2ae**, **2ak**) in 5- or 7-position on the other indole ring also showed strong antiparasitic activity. The 7-substituent could also be replaced by an ethyl residue (**2al**).

The question arose whether a hypothesis about a protein kinase as a biological target in plasmodia could be derived from the observed structure-activity relationships. Substitution in the 2-position on one or both indole rings prevents a planar arrangement of these structural elements and would be incompatible with the predicted orientations of the compounds in *Pf*PK5, *Pf*CDPK2, and *Pf*lammer (Figure 2). The structure-activity relationships derived from these observations present an inconsistent picture when viewed as a whole, making it doubtful whether plasmodial kinases are indeed the biological targets of this new class of drugs. However, it is clear that monosubstitution at one of the two nitrogen atoms is possible, whereas substitution at both indole nitrogen atoms reduces the activity. This argues against both indole nitrogen atoms interacting simultaneously with the hinge region of a plasmodial kinase. The combined substitution pattern on both indole rings also indicates that in the biologically active conformation, the two indole rings are most likely not aligned planar to each other.

Of the enzyme-ligand complexes shown in Figure 2, the structure of *Pf*GSK-3 is most consistent with the above observations. Using some 5-bromosubstituted analogs (**2b**, **2t**, **2ag**, **2ao**) as examples, this possibility was further investigated by docking experiments. The 5-monobrominated compound **2b** (IC_50 *P. falciparum*_ = 3 µM) can be unconstrainedly docked into the homology model of *Pf*GSK-3, with the bromine atom filling a hydrophobic pocket in the backspace of the ATP-binding site. An additional 5-methoxy group on the second indole ring advantageously occupies a small binding pocket in the upper region of the ATP-binding site, which could explain the enhanced inhibitory activity of this compound (**2ad**, IC_50_ *_P.f_* = 0.47 µM) (Figure S1). In this orientation, *N*-methylation on the methoxy-substituted indole ring would also be compatible (**2ao**, IC_50_ *_P.f_* = 0.54 µM). In contrast, *N*-methylation on the bromine-substituted indole ring would prevent its interaction with the hinge region, which could account for the inactivity of this compound (**2ag**, IC_50_ *_P.f_* > 10 µM).

In light of these considerations, it was hypothesized that *Pf*GSK-3 could represent the biologically relevant target structure in plasmodia. However, this assumption was refuted by testing three particularly potent compounds on isolated recombinant *Pf*GSK-3 enzyme. At a concentration of 10 µM, the particularly potent antiplasmodial agents **2k**, **2ad** and **2ae** did not inhibit *Pf*GSK-3. Therefore, the target structure responsible for the antiplasmodial activity of the substance class must be identified in further studies. In this regard, other mechanisms of action and other targets (e.g. hemoglobin degradation, hemozoin formation, β-hematin inhibition) should be considered which already have been identified for indole-derived antimalarial agents (recently reviewed by Li et al. [50]).

When used as drugs, antimicrobial agents should, as far as possible, only damage the pathogen cells without affecting the viability of the host cells. Therefore, to obtain an overview of selectivity in this class of compounds, a selection of particularly potent antiplasmodially active compounds were evaluated using the human cell line THP-1 (Table 2). While most compounds showed only weak selectivity, the three compounds **2ad**, **2ae**, and **2ai** turned out to be highly selective with selectivity indices ranging from two to three digits.

The bisindolylcyclobutenediones are compounds with multiple aromatic ring systems and a high fraction of sp^2^-hybridized carbon atoms, which also typically exhibit low solubility in aqueous media due to the solid crystal lattice structure. For compounds intended to be used as drugs, this often results in poor absorption after oral administration and thus low bioavailability. Further studies of this class of compounds must therefore be directed not only at elucidating the molecular mechanism of action of the antiplasmodial activity. Rather, derivatives must be prepared that have a higher fraction of sp^3^-hybridized carbon atoms and exhibit optimized solubility.

Nevertheless, the pronounced antiparasitic activity, novelty value of the structural class, and selectivity versus human cells warrant further exploration of these antiplasmodial agents.

## 4. Materials and Methods

### 4.1. General Information

Starting materials, reagents, and solvents were purchased from Acros Organics (Geel, Belgium), Sigma Aldrich (Steinheim, Germany), and Alfa Aesar (Karlsruhe, Germany). The known bisindolylmaleimide **8** was synthesized according to a published procedure [37]. ^1^H-NMR spectra were recorded on a Bruker Avance DRX-400 (400 MHz), a Bruker Avance III-400 (400 MHz), or a Bruker Avance II-600 (600 MHz), respectively, using DMSO-*d_6_* as solvent. The elemental analyses were recorded on a CE Instruments FlashEA 1112 Elemental Analyzer (Thermo Quest, San Jose, CA, USA). The reactions were monitored by TLC on Polygram SIL G/UV254 plates (Macherey-Nagel, Düren, Germany). Isocratic HPLC experiments were performed on a Merck Hitachi LaChrom Elite system (pump: L-2130, DAD detector: L-2450; autosampler: L-2200; column: Merck LiChroCART 125-4. LiChrospher 100 RP-18 (5 Å); elution rate 1.000 mL/min; dectection wavelength: 254 nm and 280 nm, overall run time: 15 min unless otherwise described); t_ms_ = total retention time, t_m_ = dead time; eluents: mixtures of acetonitrile and double-distilled water (Hitachi High Technologies Corporation, Tokyo, Japan). Gradient HPLC was performed on a Merck Hitachi LaChrom Elute system (pump: L2130, UV detector: L-2400; autosampler: L-2200; column: Merck LiChroCART 125-4, LiChrospher 100 RP-18 (5 Å); elution rate 1.000 mL/min; dectection wavelength: 254 nm unless otherwise described; overall run time: 25 min unless otherwise described, the eluents were mixed on-line). Gradient elution conditions: 0→2 min: acetonitrile/H_2_O 10:90, 2→12 min: acetonitrile/H_2_O 10:90→acetonitrile/H_2_O 90:10, 12→20 min: acetonitrile/H_2_O 90:10 [36]. The mass spectra were recorded on a Thermofinnigan MAT95XL (Thermo Finnigan MAT, Bremen, Germany).

### 4.2. Compound Synthesis and Characterization

#### 4.2.1. General Procedure A for the Syntheses of 3-Chloro-4-(1*H*-indol-3-yl)cyclobut-3-ene-1,2-dione Derivatives **5a**–**f**

The syntheses were performed following an adapted protocol initially developed by Schmidt et al. [40]. To squaric acid dichloride (**4**) (1.51 g, 10.0 mmol) [38] dissolved in dry diethyl ether (25 mL) and cooled to 0 °C, a solution of the appropriate indole (12.0 mmol) in the same solvent (25 mL) is added dropwise. The mixture is allowed to warm to room temperature and stirred for twenty-four hours. The resulting precipitate is filtered, washed with diethyl ether, and crystallized from chloroform/*N,N*-dimethylformamide. The product is dried at 110 °C in vacuo.

#### 4.2.2. General Procedure B for the Syntheses of 3,4-Bis(indol-3-yl)cyclobut-3-ene-1,2-diones **2l, 2m, 2p**

With reference to Green et al. [39] and Matsuoka et al. [26], the 3,4-bis(indol-3-yl)cyclobut-3-ene-1,2-diones **2l, 2m, 2p** are synthesized by the following procedure: To a suspension of 3,4-dichlorocyclobut-3-ene-1,2-dione (**4**, 0.300 g, 2.00 mmol) and anhydrous aluminum chloride (800 mg, 6.00 mmol) in dry dichloromethane, the appropriate indole (4.00 mmol) is added. The mixture is stirred for 10–65 h at room temperature with moisture protection. Subsequently, the reaction suspension is stirred in a mixture of water (50 mL) and five drops of 37% aqueous hydrochloric acid. The resulting precipitate is separated and washed with water. After drying, the residue is dissolved in acetone (10 mL) and eluted through a column of acidic aluminum oxide with acetone. The yellow or tawny fraction is collected and evaporated under reduced pressure. The solid residue is crystallized from an appropriate solvent or purified by silica gel column chromatography. The resulting product is dried at 80–90 °C in vacuo.

#### 4.2.3. General Procedure C for the Syntheses of 3,4-Bis(indol-3-yl)cyclobut-3-ene-1,2-diones **2b**–**i, 2n, 2q**–**t, 2v**–**y, 2aa**–**ac, 2ae**–**ai**

With reference to Green et al. [39] and Matsuoka et al. [26], the 3,4-bis(indol-3-yl)cyclobut-3-ene-1,2-diones **2b**–**i, 2n, 2q**–**t, 2v**–**y, 2aa**–**ac, 2ae**–**ai** are synthesized by the following procedure: To the stirred suspension of an appropriate 3-chloro-4-(1*H*-indol-3-yl)cyclobut-3-ene-1,2-dione derivative (1.00 mmol) and anhydrous aluminum chloride (400 mg, 3.00 mmol) in dry dichloromethane (20 mL), precooled to 0 °C, a solution of an appropriate indole (1.50 mmol) in the same solvent (10 mL) is added dropwise. The reaction mixture is allowed to warm to room temperature. After stirring for twenty-four to forty-eight hours at room temperature with moisture protection, the suspension is added to a mixture of water (50 mL) and 5 drops of 37% aqueous hydrochloric acid. The resulting precipitate is filtered and washed with water. After drying, the solid is eluted through a column of acidic aluminum oxide using acetone. The yellow or tawny eluent is collected and evaporated under reduced pressure. Subsequently, the solid residue is crystallized from the given solvent or purified by silica gel column chromatography using ethyl acetate/petroleum ether. The resulting product is dried at 80–90 °C in vacuo.

#### 4.2.4. General Procedure D for the Syntheses of 3,4-Bis(indol-3-yl)cyclobut-3-ene-1,2-diones **2o, 2u, 2z, 2aj**–**ao**

With reference to Green et al. [39] the 3,4-bis(indol-3-yl)cyclobut-3-ene-1,2-diones **2o, 2u, 2z, 2aj**–**ao** are synthesized by the following procedure. To the stirred suspension of an appropriate 3-chloro-4-(1*H*-indol-3-yl)cyclobut-3-ene-1,2-dione (1.00 mmol) derivative and anhydrous aluminum chloride (400 mg, 3.00 mmol) in dry dichloromethane (20 mL), precooled to 0 °C, a solution of an appropriate indole (1.50 mmol) in the same solvent (10 mL) is added dropwise. The reaction mixture is allowed to warm to room temperature. After stirring for twenty-four hours at room temperature with moisture protection, the suspension is added to a mixture of water (50 mL) and 5 drops of 37% aqueous hydrochloric acid. The resulting precipitate is filtered and washed with water. After drying, the residue is dissolved in ethyl acetate (30 mL) and washed with water (2 × 50 mL) and brine (50 mL). The aqueous layer is extracted with ethyl acetate (3 × 20 mL). After drying the combined organic layers with anhydrous sodium sulfate, the solvent is removed under reduced pressure. Subsequently, the resulting residue is crystallized from a suitable solvent and dried at 80–90 °C in vacuo.

#### 4.2.5. General Procedure E for the Syntheses of 3,4-Bis(indol-3-yl)cyclobut-3-ene-1,2-dione **2aq**

With reference to Faul et al. [41] the 3,4-bis(indol-3-yl)cyclobut-3-ene-1,2-dione derivative **2aq** is synthesized by the following procedure. To a stirred solution of the appropriate indole (5 mmol) in anhydrous toluene (8.35 mL) is added ethyl magnesium bromide (3.0 M in diethyl ether, 1.67 mL) under nitrogen. After heating to 60 °C for one hour, a solution of squaric acid dichloride (**4**) in dry tetrahydrofuran (1.64 mL) is added slowly. Subsequently, the reaction mixture is refluxed for four h under nitrogen, cooled to room temperature, and diluted with ethyl acetate (25 mL). The organic layer is washed successively with 1 M aqueous hydrochloric acid (15 mL), water (2 × 15 mL), and brine (15 mL) and dried with anhydrous sodium sulfate. After evaporation under reduced pressure, the product is crystallized from the given solvent and then dried at 80–90 °C in vacuo.

#### 4.2.6. General Procedure F for the Syntheses of 3,4-Bis(indol-3-yl)cyclobut-3-ene-1,2-diones **2a, 2ad**, **2ar**

With reference to Faul et al. [41] the 3,4-bis(indol-3-yl)cyclobut-3-ene-1,2-dione derivatives **2a**, **2ad**, **2ar** are synthesized by the following procedure. To a stirred solution of an appropriate indole (3 mmol) in anhydrous toluene (8.35 mL) is added ethyl magnesium bromide (3.0 M in diethyl ether, 1.67 mL) under nitrogen. After heating to 60 °C for one hour, a suspension of an appropriate 3-chloro-4-indol-3-ylcyclobut-3-ene-1,2-dione derivative in a mixture of dry toluene/diethyl ether/tetrahydrofuran (8.35 mL/1 mL/2.67 mL) is added slowly. Subsequently, the reaction mixture is refluxed for four h under nitrogen, cooled to room temperature, and diluted with ethyl acetate (25 mL). The organic layer is washed successively with 1 M aqueous hydrochloric acid (15 mL), water (2 × 15 mL), brine (15 mL), and dried with anhydrous sodium sulfate. After evaporation under reduced pressure, the product is crystallized from the given solvent and then dried at 80–90 °C in vacuo.

#### 4.2.7. Synthesis Procedures and Characterization Data for Individual Compounds

##### 3-Chloro-4-(1H-indol-3-yl)cyclobut-3-ene-1,2-dione (**5a**)

Synthesis according to General Procedure A from squaric acid dichloride (**4**, 3.09 g, 0.02 mol) and 1*H*-indole (2.39 g, 0.02 mol). The yield of the ocher solid was 3.10 g (67%). IR (KBr): 3193 cm^−1^ (NH), 1773 cm^−1^, 1755 cm^−1^ (C=O), 1616 cm^−1^ (C=C, arom.); ^1^H-NMR (600 MHz, DMSO-*d*_6_): δ (ppm) = 7.20 (ddd, *J* = 8.1, 7.0, 1.2 Hz, 1H, ArH), 7.22 (ddd, *J* = 8.1, 7.1, 1.3 Hz, 1H, ArH), 7.51 (dt, *J* = 8.1, 1.0 Hz, 1H, ArH), 8.20 (d, *J* = 3.0 Hz, 1H, ArH), 8.30 (ddt, *J* = 7.9, 1.4, 0.8 Hz, 1H, ArH), 12.27 (s, 1H, NH); ^13^C-NMR (151 MHz, DMSO-*d_6_*): δ (ppm) = 112.3, 121.2, 121.6, 122.9, 128.7 (CH), 105.8, 124.7, 136.4, 171.2, 192.8 (2C), 193.3 (C); C_12_H_6_ClNO_2_ [231.64]; Anal. calcd. for C_12_H_6_ClNO_2_: C 62.22, H 2.61, N 6.05; found C 61.95, H 2.52, N 5.82; MS (EI): *m*/*z* (%) = 231 [M]^+^∙(40), 203 [M^+^ − 28] (6), 175 [M^+^ − 56] (100).

##### 3-Chloro-4-(1-methyl-1H-indol-3-yl)cyclobut-3-ene-1,2-dione (**5b**)

Synthesis according to General Procedure A from squaric acid dichloride (**4**, 453 mg, 3.00 mmol) and 1-methyl-1*H*-indole (0.44 mL, 3.52 mmol). The yield of the yellow solid was 0.21 g (27%). IR (KBr): 1812 cm^−1^, 1777 cm^−1^, 1738 cm^−1^ (C=O, C=C), 1619 cm^−1^, 1556 cm^−1^, 1509 cm^−1^ (C=C, arom.); ^1^H-NMR (400 MHz, DMSO-*d*_6_): δ (ppm) = 3.93 (s, 3H, CH_3_), 7.26 (ddd, *J* = 8.0, 7.1, 1.1 Hz, 1H, ArH), 7.32 (ddd, *J* = 8.2, 7.1, 1.3 Hz, 1H, ArH), 7.57 (dt, *J* = 8.2, 0.9 Hz, 1H, ArH), 8.26 (s, 1H, ArH), 8.29 – 8.32 (m, 1H, ArH); ^13^C-NMR (101 MHz, DMSO-*d*_6_): δ (ppm) = 33.2 (CH_3_), 110.8, 121.5, 121.9, 123.0, 132.4 (CH), 104.9, 125.2, 137.1, 170.7, 192.8 (2C), 193.2 (C); C_13_H_8_ClNO_2_ [245.66]; Anal. calcd. for C_13_H_8_ClNO_2_: C 63.56, H 3.28, N 5.70; found C 63.44, H 3.23, N 5.59; MS (EI): *m*/*z* (%) = 245 [M]^+∙^(32), 217 [M^+^ − 28] (2), 189 [M^+^ − 56] (100).

##### 3-Chloro-4-(5-methoxy-1H-indol-3-yl)cyclobut-3-ene-1,2-dione (**5c**)

Synthesis according to General Procedure A from squaric acid dichloride (**4**, 1.53 g, 10.1 mmol) and 5-methoxy-1*H*-indole (1.51 g, 10.3 mmol). The yield of the green powder was 2.22 g (74%). IR (KBr): 3184 cm^−1^ (NH), 1800 cm^−1^, 1776 cm^−1^, 1741 cm^−1^ (C=O, C=C), 1631 cm^−1^, 1543 cm^−1^ (C=C, arom.); ^1^H-NMR (400 MHz, DMSO-*d*_6_): δ (ppm) = 3.78 (s, 3H, CH_3_), 6.88 (dd, *J* = 8.8, 2.5 Hz, 1H, ArH), 7.40 (dd, *J* = 8.8, 0.6 Hz, 1H, ArH), 7.84 (d, *J* = 2.5 Hz, 1H, ArH), 8.13 (d, *J* = 3.1 Hz, 1H, ArH), 11.41 (s, 1H, NH); ^13^C-NMR (101 MHz, DMSO-*d*_6_): δ (ppm) = 60.6 (CH_3_), 109.2, 117.9, 118.4, 134.5 (CH), 111.0, 111.1, 130.9, 136.7, 160.1, 176.6, 197.9, 198.0 (C); C_13_H_8_ClNO_3_ [261.52]; Anal. calcd. for C_13_H_8_ClNO_3_: C 59.65, H 3.06, N 5.35; found C 59.38, H 3.09, N 5.32; MS (EI): *m*/*z* (%) = 261 [M]^+^∙(45), 233 [M^+^ − 28] (2), 205 [M^+^ − 56] (100).

##### 3-Chloro-4-(2-phenyl-1H-indol-3-yl)cyclobut-3-ene-1,2-dione (**5d**)

Synthesis according to General Procedure A from squaric acid dichloride (**4**, 1.51 g, 10.0 mmol) and 2-phenyl-1*H*-indole (2.08 g, 10.8 mmol). The crude product was purified by column chromatography on silica gel using ethyl acetate/petroleum ether (1/1). The yield of the orange powder was 2.00 g (65%). IR (KBr): 3219 cm^−1^ (NH), 1769 cm^−1^, 1743 cm^−1^ (C=O); ^1^H-NMR (400 MHz, DMSO-*d*_6_): δ (ppm) = 7.16 (ddd, *J* = 8.1, 7.1, 1.1 Hz, 1H, ArH), 7.23 (ddd, *J* = 8.1, 7.1, 1.3 Hz, 1H, ArH), 7.40–7.50 (m, 4H, ArH), 7.54–7.62 (m, 2H, ArH), 8.06 (dd, *J* = 7.7, 1.0 Hz, 1H, ArH), 12.30 (s, 1H, NH); ^13^C-NMR (101 MHz, DMSO-*d*_6_): δ (ppm) = 111.7, 120.6, 121.7, 122.8, 128.1, 128.6, 129.0 (CH), 102.1, 126.9, 128.3, 131.9, 136.5, 140.0, 173.6, 193.3, 196.2 (C); C_18_H_10_ClNO_2_ [307.73]; Anal. calcd. for C_18_H_10_ClNO_2_: C 70.26, H 3.28, N 4.55; found C 70.08, H 3.26, N 4.46; MS (EI): *m*/*z* (%) = 307 [M]^+^ (45), 279 [M^+^ − 28] (14), 251 [M^+^ − 56] (67), 216 [M^+^ − 91] (100).

##### 3-(5-Bromo-1H-indol-3-yl)-4-chlorocyclobut-3-ene-1,2-dione (**5e**)

Synthesis according to General Procedure A from squaric acid dichloride (**4**, 1.97 g, 0.01 mol) and 5-bromo-1*H*-indole (1.50 g, 0.01 mol). The yield of the ocher powder was 432 mg (14%). IR (KBr): 3460 cm^−1^ (NH), 1817 cm^−1^, 1773 cm^−1^, 1740 cm^−1^ (C=O, C=C), 1614 cm^−1^, 1550 cm^−1^ (C=C, arom.); ^1^H-NMR (400 MHz, DMSO-*d_6_*): δ (ppm) = 7.37 (dd, *J* = 8.7, 2.0 Hz, 1H, ArH), 7.49 (dd, *J* = 8.6, 0.5 Hz, 1H, ArH), 8.21 (d, *J* = 3.0 Hz, 1H, ArH), 8.49 (d, *J* = 2.0 Hz, 1H, ArH), 12.42 (s, 1H, NH); ^13^C-NMR (101 MHz, DMSO-*d_6_*): δ (ppm) = 114.4, 124.0, 125.4, 129.4 (CH), 105.7, 113.6, 126.6, 135.2, 170.8, 193.2 (2C), 194.6 (C); C_12_H_5_BrClNO_2_ [310.53]; MS (EI): *m*/*z* (%) = 309 [M]^+^∙(28), 253 [M^+^ − 56] (100); Anal. calcd. for C_12_H_5_BrClNO_2_: C 46.41, H 1.62, N 4.51; found C 46.57, H 1.56, N 4.52.

##### 3-Chloro-4-(2-methyl-1H-indol-3-yl)cyclobut-3-ene-1,2-dione (**5f**)

Synthesis according to General Procedure A from squaric acid dichloride (**4**, 1.21 g, 9.17 mmol) and 2-methyl-1*H*-indole (1.49 g, 9.84 mmol). The yield of the green powder was 2.25 g (83%). IR (KBr): 3447 cm^−1^ (NH), 1765 cm^−1^, 1736 cm^−1^ (C=O); ^1^H-NMR (400 MHz, DMSO-*d*_6_): δ (ppm) = 2.80 (s, 3H, CH_3_), 7.12 (dtd, *J* = 16.0, 7.2, 1.4 Hz, 2H, ArH), 7.35 (dd, *J* = 7.2, 1.6 Hz, 1H, ArH), 8.21 (dd, *J* = 7.4, 1.5 Hz, 1H, ArH), 12.12 (s, 1H, NH); ^13^C-NMR (100.7 MHz, DMSO-*d*_6_): δ (ppm) = 14.1 (CH_3_), 111.1, 120.7, 121.6, 122.0 (CH), 103.8, 126.6, 136.2, 141.8, 173.4, 192.5, 193.7, 195.2 (C); C_13_H_8_O_2_ClN [245.66]; MS (EI): *m*/*z* (%) = 245 [M]^+^∙(36), 217 [M^+^ − 28] (5), 189 [M^+^ − 56] (100); HRMS (EI) (*m*/*z*): Calcd. for [M]^+^ = 245.02381; found [M]^+^ = 245.02362.

##### 3,4-Bis(1H-indol-3-yl)cyclobut-3-ene-1,2-dione (**2a**)

Synthesis according to General Procedure F from 3-chloro-4-(1*H*-indol-3-yl)cyclobut-3-ene-1,2-dione (**5a**, 0.262 g, 1.13 mmol) and 1*H*-indole (0.360 mg, 3.08 mmol). Crystallization from ethanol/toluene yielded a yellow powder (59 mg, 66%).

IR (KBr): 3388 cm^−1^ (NH), 1766 cm^−1^, 1704 cm^−1^ (C=O), 1616 cm^−1^, 1589 cm^−1^, 1513 cm^−1^ (C=C, arom.), ^1^H-NMR (400 MHz, DMSO-*d*_6_): δ (ppm) = 7.17 (ddd, *J* = 8.1, 7.1, 1.1 Hz, 2H), 7.28 (ddd, *J* = 8.2, 7.1, 1.2 Hz, 2H), 7.58 (dt, *J* = 8.2, 0.9 Hz, 2H), 7.93 (dt, *J* = 8.0, 0.9 Hz, 2H), 8.26 (d, *J* = 3.0 Hz, 2H), 12.44 (s, 2H, NH), ^13^C-NMR (101 MHz, DMSO-*d*_6_): δ (ppm) = 112.6, 121.3, 122.6, 123.0, 131.1 (CH), 106.3, 125.0, 136.8, 177.0, 194.8 (C); C_20_H_12_N_2_O_2_ (312.33); Anal. calcd. for C_20_H_12_N_2_O_2_: C 76.91, H 3.87, N 8.97; found C 76.41, H 3.70, N 8.87; MS (EI): *m*/*z* (%) = 312 [M]^+^ (35), 256 [M^+^ − 56] (100); isocratic HPLC: 97.1% at 254 nm, 97.6% at 280 nm, t_ms_ = 3.63 min, t_m_ (DMSO) = 1.15 min (ACN/H_2_O 50:50); gradient HPLC: 95.1% at 254 nm, t_ms_ = 11.0 min, t_m_ (DMSO) = 1.25 min; λ_max_ (nm): 271, 331, 410.

##### 3-(5-Bromo-1H-indol-3-yl)-4-(1H-indol-3-yl)cyclobut-3-ene-1,2-dione (**2b**)

Synthesis according to General Procedure C (reaction time: 48 h) from 3-chloro-4-(1*H*-indol-3-yl)cyclobut-3-ene-1,2-dione (**5a**, 256 mg, 1.11 mmol) and 5-bromo-1*H*-indole (392 mg, 2.00 mmol). The yield of the tawny powder was 166 mg (38%). IR (KBr): 3425 cm^−1^ (NH), 3127 cm^−1^ (CH, arom.), 2919 cm^−1^ (CH, aliph.), 1748 cm^−1^, 1714 cm^−1^ (C=O); ^1^H-NMR (400 MHz, DMSO-*d_6_*): δ (ppm) = 7.19 (ddd, *J* = 8.1, 7.1, 1.1 Hz, 1H, ArH), 7.30 (ddd, *J* = 8.2, 7.1, 1.2 Hz, 1H, ArH), 7.41 (dd, *J* = 8.6, 2.0 Hz, 1H, ArH), 7.51–7.63 (m, 2H, ArH), 7.90 (ddt, *J* = 8.0, 1.2, 0.6 Hz, 1H, ArH), 8.22 (d, *J* = 2.0 Hz, 1H, ArH), 8.26 (d, *J* = 3.0 Hz, 1H, ArH), 8.35 (d, *J* = 3.1 Hz, 1H, ArH), 12.50 (d, *J* = 3.2 Hz, 1H, NH), 12.57 (d, *J* = 3.3 Hz, 1H, NH); ^13^C-NMR (151 MHz, DMSO-*d*_6_): δ (ppm) = 112.7, 114.6, 121.4, 122.6, 123.1, 124.8, 125.6, 131.2, 132.0 (CH), 106.0, 106.2, 113.8, 124.9, 127.0, 135.5, 136.9, 176.3, 177.3, 194.6, 194.7 (C); C_20_H_11_BrN_2_O_2_ [391.22]; Anal. calcd. for C_20_H_11_BrN_2_O_2_: C 61.40, H 2.83, N 7.16; found C 61.38, H 2.74, N 6.85; MS (EI): *m*/*z* (%) = 390 [M]^+^ (29), 334 [M^+^ − 56] (100); isocratic HPLC: 99.4% at 254 nm and 99.6% at 280 nm, t_ms_ = 5.84 min, t_m_ = 1.11 min (ACN/H_2_O 50:50); λ_max_ (nm): 273, 329, 346; gradient HPLC: 98.0% at 254 nm, t_ms_ = 11.9 min, t_m_ = 1.25 min.

##### 3-(5-Chloro-1H-indol-3-yl)-4-(1H-indol-3-yl)cyclobut-3-ene-1,2-dione (**2c**)

Synthesis according to General Procedure C (reaction time: 20 h) from 3-chloro-4-(1*H*-indol-3-yl)cyclobut-3-ene-1,2-dione (**5a**, 241 mg, 1.04 mmol) and 5-chloro-1*H*-indole (227 mg, 1.50 mmol). Boiling in ethanol/toluene yielded a yellowish powder (133 mg, 38%). IR (KBr): 3395 cm^−1^ (NH), 1749 cm^−1^, 1714 cm^−1^ (C=O), 1618 cm^−1^, 1539 cm^−1^ (C=C, arom.); ^1^H-NMR (600 MHz, DMSO-*d_6_*): δ (ppm) = 7.20 (ddd, *J* = 8.1, 7.0, 1.1 Hz, 1H, ArH), 7.27–7.33 (m, 2H, ArH), 7.57–7.63 (m, 2H, ArH), 7.91 (ddt, *J* = 8.1, 1.4, 0.7 Hz, 1H, ArH), 8.08 (dt, *J* = 2.1, 0.6 Hz, 1H, ArH), 8.28 (d, *J* = 3.1 Hz, 1H, ArH), 8.35 (d, *J* = 3.1 Hz, 1H, ArH), 12.50 (d, *J* = 3.1 Hz, 1H, NH), 12.58 (d, *J* = 3.0 Hz, 1H, NH); ^13^C-NMR (151 MHz, DMSO-*d_6_*): δ (ppm) = 112.7, 114.2, 121.4, 121.9, 122.6, 123.1, 123.2, 131.3, 132.2 (CH), 106.2. 106.2, 124.9, 125.8, 126.4, 135.3, 136.9, 176.4, 177.4, 194.7, 194.8 (C); C_20_H_11_ClN_2_O_2_ [347.77]; Anal. calcd. for C_20_H_11_ClN_2_O_2_: C 69.27, H 3.20, N 8.08; found C 68.87, H 3.10, N 7.93; MS (EI): *m*/*z* (%) = 346 [M]^+∙^(23), 290 [M^+^-56] (100); isocratic HPLC: 95.9% at 254 nm and 96.8% at 280 nm, t_ms_ = 5.17 min, t_m_ = 1.11 min (ACN/H_2_O 50:50); λ_max_ (nm): 273, 328 and 347; gradient HPLC: 98.3% at 254 nm, t_ms_ = 11.8 min, t_m_ = 1.25 min.

##### 3-(1H-Indol-3-yl)-4-(5-methoxy-1H-indol-3-yl)cyclobut-3-ene-1,2-dione (**2d**)

Synthesis according to General Procedure C (reaction time: 20 h) from 3-chloro-4-(1*H*-indol-3-yl)cyclobut-3-ene-1,2-dione (**5a**, 241 mg, 1.04 mmol) and 5-methoxy-1*H*-indole (322 mg, 2.19 mmol). The yield of the yellow powder was 40 mg (11%). IR (KBr): 3298 cm^−1^ (NH), 3144 cm^−1^ (CH, arom.), 2964 cm^−1^ (CH, aliph.), 1748 cm^−1^, 1704 cm^−1^ (C=O); ^1^H-NMR (600 MHz, DMSO-*d_6_*): δ (ppm) = 3.53 (s, 3H, CH_3_), 6.89 (dd, *J* = 8.8, 2.5 Hz, 1H, ArH), 7.15 (ddd, *J* = 8.0, 7.0, 1.0 Hz, 1H, ArH), 7.27 (ddd, *J* = 8.2, 7.1, 1.1 Hz, 1H, ArH), 7.31–7.34 (m, 1H, ArH), 7.47 (dd, *J* = 8.8, 0.5 Hz, 1H, ArH), 7.58 (dt, *J* = 8.1, 0.9 Hz, 1H, ArH), 7.91 (dt, *J* = 8.0, 0.9 Hz, 1H, ArH), 8.21 (s, 1H, ArH), 8.25 (s, 1H, ArH), 12.38 (s, 2H, NH); ^13^C-NMR (151 MHz, DMSO-*d*_6_): δ (ppm) = 54.8 (CH_3_), 104.7, 112.5, 112.8, 113.3, 121.1, 122.5, 122.9, 131.0, 131.4 (CH), 106.2, 106.3, 125.1, 125.5, 131.6, 136.6, 154.5, 176.3, 176.9, 194.4, 195.0 (C); C_21_H_14_N_2_O_3_ [342.35]; MS (EI): *m*/*z* (%) = 342 [M]^+^ (35), 286 [M^+^-56] (100); HRMS (EI) (*m*/*z*): Calcd. for [M]^+^ = 342.09989; found [M]^+^ = 342.10005; isocratic HPLC: 98.6% at 254 nm and 98.9% at 280 nm, t_ms_ = 3.49 min, t_m_ = 1.11 min (ACN/H_2_O 50:50); λ_max_ (nm): 229, 273, 336; gradient HPLC: 97.6% at 254 nm, t_ms_ = 10.8 min, t_m_ = 1.25 min.

##### 3-[5-(Benzyloxy)-1H-indol-3-yl]-4-(1H-indol-3-yl)cyclobut-3-ene-1,2-dione (**2e**)

Synthesis according to General Procedure C (reaction time: 10 h) from 3-chloro-4-(1*H*-indol-3-yl)cyclobut-3-ene-1,2-dione (**5a**, 262 mg, 1.13 mmol) and 5-benzyloxy-1*H*-indole (335 mg, 1.50 mmol). The oily product obtained after column chromatography on Al_2_O_3_ was taken up in ethyl acetate (3 mL). Upon addition of petroleum ether a tan precipitate appeared, which was subsequently crystallized from petroleum ether/ethyl acetate/ethanol. The yield of the tan powder was 20 mg (5%). IR (KBr): 3405 cm^−1^ (NH), 1750 cm^−1^, 1712 cm^−1^ (C=O), 1628 cm^−1^, 1540 cm^−1^, 1512 cm^−1^ (C=C, arom.); ^1^H-NMR (600 MHz, DMSO-*d_6_*): δ (ppm) = 4.73 (s, 2H, CH_2_), 6.95 (dd, *J* = 8.8, 2.5 Hz, 1H, ArH), 7.15 (ddd, *J* = 8.1, 7.1, 1.0 Hz, 1H, ArH), 7.23–7.37 (m, 6H, ArH), 7.47 (d, *J* = 8.8 Hz, 2H, ArH), 7.60 (dt, *J* = 8.2, 0.9 Hz, 1H, ArH), 7.87 (dt, *J* = 8.0, 1.0 Hz, 1H, ArH), 8.23 (d, *J* = 3.0 Hz, 1H, ArH), 8.27 (d, *J* = 3.2 Hz, 1H, ArH), 12.36 (d, *J* = 3.2 Hz, 1H, NH), 12.44 (d, *J* = 3.1 Hz, 1H, NH); ^13^C-NMR (151 MHz, DMSO-*d_6_*): δ (ppm) = 69.3 (CH_2_), 106.2, 112.6, 113.4, 113.5, 121.2, 122.5, 123.1, 127.5, 128.3, 131.1, 131.7 (CH), 106.3, 106.4, 125.3, 125.5, 127.3, 127.7, 128.3, 131.8, 136.8, 137.1, 153.7, 176.3, 177.0, 194.5, 195.1 (C); C_27_H_18_N_2_O_3_ [418.45]; MS (EI): *m*/*z* (%) = 418 [M]^+^ (59), 362 [M^+^ - 56] (100); HRMS (EI) (*m*/*z*): Calcd. for [M]^+^ = 418.13119, found [M]^+^ = 418.13169; isocratic HPLC: 95.2% at 254 nm and 95.5% at 280 nm, t_ms_ = 7.26 min, t_m_ = 1.11 min (ACN/H_2_O 50:50); λ_max_ (nm): 273; gradient HPLC: 95.0% at 254 nm, t_ms_ = 12.4 min, t_m_ = 1.25 min.

##### 3-[2-(1H-Indol-3-yl)-3,4-dioxocyclobut-1-ene-1-yl]-1H-indol-5-carbonitrile (**2f**)

Synthesis according to General Procedure C (reaction time: 20 h) from 3-chloro-4-(1*H*-indol-3-yl)cyclobut-3-ene-1,2-dione (**5a**, 265 mg, 1.14 mmol) and 1*H*-indole-5-carbonitrile (213 mg, 1.50 mmol). The residue obtained after column chromatography on Al_2_O_3_ was crystallized from ethanol/toluene. The yield of the yellow powder was 200 mg (52%). IR (KBr): 3300 cm^−1^ (NH), 2229 cm^−1^ (C≡N), 1750 cm^−1^, 1723 cm^−1^ (C=O), 1619 cm^−1^, 1554 cm^−1^, 1528 cm^−1^ (C=C, arom.); ^1^H-NMR (600 MHz, DMSO-*d_6_*): δ (ppm) = 7.19 (ddd, *J* = 8.1, 7.1, 1.1 Hz, 1H, ArH), 7.30 (ddd, *J* = 8.2, 7.0, 1.1 Hz, 1H, ArH), 7.60 (dt, *J* = 8.1, 0.9 Hz, 1H, ArH), 7.65 (dd, *J* = 8.5, 1.6 Hz, 1H, ArH), 7.75 (dd, *J* = 8.5, 0.7 Hz, 1H, ArH), 7.88 (ddt, *J* = 8.0, 1.3, 0.7 Hz, 1H, ArH), 8.39 (d, *J* = 3.0 Hz, 1H, ArH), 8.42 (d, *J* = 3.1 Hz, 1H, ArH), 8.47 (dt, *J* = 1.5, 0.7 Hz, 1H, ArH), 12.54–12.58 (m, 1H, NH), 12.82–12.86 (m, 1H, NH); ^13^C-NMR (151 MHz, DMSO-*d_6_*): δ (ppm) = 112.8, 114.1, 121.6, 122.6, 123.3, 125.1, 127.8, 131.7, 132.8 (CH), 103.3, 106.2, 106.9, 120.2, 124.9, 125.2, 137.0, 138.6, 175.6, 178.1, 194.5, 195.0 (C); C_21_H_11_N_3_O_2_ [337.34]; MS (EI): *m*/*z* (%) = 337 [M]^+^ (12), 281 [M^+^ − 56] (100); HRMS (EI) (*m*/*z*): Calcd. for [M]^+^∙= 337.08458, found [M]^+^ = 337.08486; isocratic HPLC: 97.0% at 254 nm and 97.2% at 280 nm, t_ms_ = 6.42 min, t_m_ = 1.11 min (ACN/H_2_O 40:60); λ_max_ (nm): 224, 327 and 346; gradient HPLC: 98.5% at 254 nm, t_ms_ = 10.7 min, t_m_ = 1.25 min.

##### 3-(1H-Indol-3-yl)-4-(2-methyl-1H-indol-3-yl)cyclobut-3-ene-1,2-dione (**2g**)

Synthesis according to General Procedure C (reaction time: 19 h) from 3-chloro-4-(2-methyl-1*H*-indol-3-yl)cyclobut-3-ene-1,2-dione (**5e**, 233 mg, 0.95 mmol) and 1*H*-indole (233 mg, 1.99 mmol). The yield of the yellow powder was 52 mg (17%). IR (KBr): 3367 cm^−1^, 3256 cm^−1^ (NH), 1759 cm^−1^, 1706 cm^−1^ (C=O), 1618 cm^−1^, 1555 cm^−1^,1522 cm^−1^ (C=C, arom.); ^1^H-NMR (600 MHz, DMSO-*d*_6_): δ (ppm) = 2.55 (s, 3H), 6.98 (ddd, *J* = 7.9, 7.1, 1.0 Hz, 1H), 7.08 (ddd, *J* = 8.0, 7.1, 1.0 Hz, 1H), 7.14–7.18 (m, 2H), 7.23 (ddd, *J* = 8.2, 7.1, 1.1 Hz, 1H), 7.44–7.47 (m, 1H), 7.53 (dt, *J* = 8.1, 0.9 Hz, 1H), 7.84 (d, *J* = 8.1 Hz, 1H), 7.91 (s, 1H), 12.17 (s, 1H), 12.38 (s, 1H); ^13^C-NMR (151 MHz, DMSO-*d*_6_): δ (ppm) = 13.9 (CH_3_), 111.6, 112.5, 120.3, 121.1, 121.4, 121.9, 122.1, 123.0, 132.1 (CH), 103.9, 106.6, 125.3, 125.4, 136.4, 136.5, 141.2, 178.4, 178.7, 194.7, 195.8 (C); C_21_H_14_N_2_O_2_ [326.36]; Anal. calcd. for C_21_H_14_N_2_O_2_: C 77.29, H 4.32, N 8.58; found C 77.00, H 4.20, N 8.49; MS (EI): *m*/*z* (%) = 326 [M]^+^ (30), 270 [M^+^ − 56] (100); isocratic HPLC: 96.7% at 254 nm, 96.5% at 280 nm, t_ms_ = 3.85 min, t_m_ = 1.11 min (ACN/H_2_O 50:50); λ_max_ (nm): 273; gradient HPLC: 95.1% at 254 nm, t_ms_ = 11.1 min, t_m_ = 1.25 min.

##### 3-(1H-Indol-3-yl)-4-(2-phenyl-1H-indol-3-yl)cyclobut-3-ene-1,2-dione (**2h**)

Synthesis according to General Procedure C (reaction time: 21 h) from 3-chloro-4-(1*H*-indol-3-yl)cyclobut-3-ene-1,2-dione (**5a**, 238 mg, 1.03 mmol) and 2-phenyl-1*H*-indole (430 mg, 3.25 mmol). The yield of the yellow powder was 280 mg (70%). IR (KBr): 3248 cm^−1^ (NH), 3066 cm^−1^ (CH, arom.), 1752 cm^−1^, 1712 cm^−1^ (C=O); ^1^H-NMR (400 MHz, DMSO-*d_6_*): δ (ppm) = 6.99–7.11 (m, 2H, ArH), 7.12–7.35 (m, 5H, ArH), 7.38–7.48(m, 2H, ArH), 7.48–7.60 (m, 3H, ArH), 7.75–7.81 (m, 1H, ArH), 7.85 (d, *J* = 7.9 Hz, 1H, ArH), 12.24 (d, *J* = 3.0 Hz, 1H, NH), 12.44 (s, 1H, NH); ^13^C-NMR (101 MHz, DMSO-*d*_6_): δ (ppm) = 112.1, 112.4, 120.7, 121.3, 121.5, 122.2, 122.9, 122.9, 128.2, 128.3, 128.6, 132.1 (CH), 102.7, 106.9, 125.1, 126.4, 131.5, 136.5, 136.8, 139.8, 178.9, 180.2, 194.2, 196.8 (C); C_26_H_16_N_2_O_2_ [388.43]; Anal. calcd. for C_26_H_16_N_2_O_2_: C 80.40, H 4.15, N 7.21; found C 79.99, H 4.11, N 6.89; MS (EI): *m*/*z* (%) = 388 [M]^+^ (17), 360 [M^+^ − 28] (8), 332 [M^+^ − 56] (100); isocratic HPLC: 98.5% at 254 nm and 98.6% at 280 nm, t_ms_ = 5.69 min, t_m_ = 1.11 min (ACN/H_2_O 50:50); λ_max_ (nm): 280, 297, 331; gradient HPLC: 98.0% at 254 nm, t_ms_ = 11.7 min, t_m_ = 1.25 min.

##### 3,4-Bis(1-methyl-1H-indol-3-yl)cyclobut-3-ene-1,2-dione (**2i**)

Synthesis according to General Procedure C (reaction time: 24 h) from 3-chloro-4-(1-methyl-1*H*-indol-3-yl)cyclobut-3-ene-1,2-dione (**5b**, 248 mg, 1.01 mmol) and 1-methyl-1*H*-indol (0.190 mL, 1.52 mmol). Due to the poor solubility, the green brown precipitate was boiled successively in ethanol/toluene and then in acetone for purification avoiding column chromatography. The yield of the yellow-green powder was 300 mg (56%). IR (KBr): 1766 cm^−1^, 1731 cm^−1^ (C=O), 1615 cm^−1^, 1576 cm^−1^, 1561 cm^−1^, 1517 cm^−1^ (C=C, arom.); ^1^H-NMR (600 MHz, chloroform-*d*): δ (ppm) = 3.93 (s, 6H, CH_3_), 7.15 (ddd, *J* = 8.1, 7.1, 1.0 Hz, 2H, ArH), 7.35 (dd, *J* = 8.2, 7.1 Hz, 2H, ArH), 7.42–7.47 (m, 2H, ArH), 7.79 (dd, *J* = 8.0, 0.9 Hz, 2H, ArH), 8.01 (s, 2H, ArH); ^13^C-NMR (151 MHz, Chloroform-*d*): δ (ppm) = 33.7 (CH_3_), 110.1, 121.7, 123.5, 123.6, 125.7 (CH), 106.6, 134.1, 137.7, 176.4, 195.4 (C); C_22_H_16_N_2_O_2_ [340.38]; MS (EI): *m*/*z* (%) = 340 [M]^+∙^(32), 284 [M^+^ − 56] (100); HRMS (EI) (*m*/*z*): Calcd. for [M]^+^ = 340.12063, found [M]^+^∙= 340.12124; isocratic HPLC: 95.1% at 254 nm and 97.4% at 280 nm, t_ms_ = 4.53 min, t_m_ = 1.11 min (ACN/H_2_O 60:40); λ_max_ (nm): 224, 273 and 333; gradient HPLC: 95.1% at 254 nm, t_ms_ = 12.8 min, t_m_ = 1.25 min.

##### 3-{1-[3-(Dimethylamino)propyl]-1H-indol-3-yl}-4-(1H-indol-3-yl)cyclobut-3-ene-1,2-dione (**2k**)

To a suspension of 3-chloro-4-(1*H*-indol-3-yl)cyclobut-3-ene-1,2-dione (5**a**, 235 mg, 1.01 mmol) in anhydrous dichloromethane (10 mL) at 0 °C, anhydrous AlCl_3_ (400 mg, 3.00 mmol) was added. A solution of 3-(1*H*-indol-1-yl)-*N,N*-dimethylpropane-1-amine (0.248 mL, 1.20 mmol) in dichloromethane (10 mL) was slowly added dropwise with stirring,. While stirring was continued for 24 h, the temperature was allowed to rise from 0 °C to RT. The reaction mixture was then poured into water (50 mL) with stirring and filtered. The filtrate was added to ethyl acetate (100 mL). The mixture was washed with sat. Na_2_CO_3_ solution (2 × 50 mL), water (2 × 50 mL) and saturated NaCl solution (2 × 50 mL). After drying the organic phase over anhydrous Na_2_SO_4_, it was concentrated under reduced pressure. The resulting oily residue was eluted through silica gel with ethanol/ethyl acetate/triethylamine (50/50/1), wherein the yellow phase was collected and concentrated. The residue was then crystallized from acetone and dried in vacuo at 80–90 °C. The yield of yellow powder was 35 mg (9%). IR (KBr): 3426 cm^−1^ (NH), 1750 cm^−1^ (C=O), 1615 cm^−1^, 1553 cm^−1^, 1521 cm^−1^ (C=C, arom.); ^1^H-NMR (600 MHz, DMSO-*d*): δ (ppm) = 2.01 (quin, *J* = 7.1 Hz, 2H, CH_2_-CH_2_-CH_2_-N(CH_3_)_2_), 2.23 (s, 6H, CH_3_), 2.35 (s, br, 2H, CH_2_-CH_2_-CH_2_-N(CH_3_)_2_), 4.39 (t, *J* = 7.0 Hz, 2H, CH_2_-CH_2_-CH_2_-N(CH_3_)_2_), 7.18 (ddd, *J* = 8.0, 7.0, 1.0 Hz, 1H, ArH), 7.22 (ddd, *J* = 8.1, 7.0, 0.9 Hz, 1H, ArH), 7.32 (dddd, *J* = 34.1, 8.1, 7.1, 1.2 Hz, 2H, ArH), 7.59 (dt, *J* = 8.3, 1.0 Hz, 1H, ArH), 7.71 (dt, *J* = 8.3, 0.9 Hz, 1H, ArH), 7.92 (dt, *J* = 8.0, 1.0 Hz, 1H, ArH), 7.98 (dt, *J* = 8.1, 1.0 Hz, 1H, ArH), 8.29 (d, *J* = 5.3 Hz, 2H, ArH), 12.48 (s, 1H, NH); ^13^C-NMR (151 MHz, DMSO-*d*_6_): δ (ppm) = 26.3 (CH_2_-CH_2_-CH_2_-N(CH_3_)_2_), 44.2 (CH_2_-CH_2_-CH_2_-N(CH_3_)_2_), 44.6 (2C, CH_3_), 55.5 (CH_2_-CH_2_-CH_2_-N(CH_3_)_2_), 111.1, 112.7, 121.3, 121.6, 122.7, 123.0, 123.1, 123.2, 131.2, 133.8 (CH), 105.6, 106.3, 125.0, 125.6, 136.7, 136.9, 176.4, 177.0, 194.7, 194.8 (C); C_25_H_23_N_3_O_2_ [397.48]; MS (EI): *m*/*z* (%) = 397 [M]^+^∙(79), 269 [M^+∙^-128] (100); HRMS (EI) (*m*/*z*): Calcd. for [M]^+^∙= 397.17848, found [M]^+^∙= 397.17823; isocratic HPLC: 95.1% at 254 nm and 97.4% at 280 nm, t_ms_ = 3.13 min, t_m_ = 1.11 min (ACN/triethyl ammonium sulfate buffer pH 2.7: 33:67); λ_max_ (nm): 272, 335 and 345.

##### 3,4-Bis(2-methyl-1H-indol-3-yl)cyclobut-3-ene-1,2-dione (**2l**)

Synthesis according to General Procedure B (reaction time: 30 h) from squaric acid dichloride (**4**, 302 mg, 2.00 mmol) and 2-methyl-1*H*-indole (525 mg, 4.01 mmol). Crystallization from ethyl acetate yieled a yellow powder (59 mg, 56%). IR (KBr): 3385 cm^−1^ (NH), 1760 cm^−1^, 1720 cm^−1^ (C=O), 1619 cm^−1^, 1557 cm^−1^,1518 cm^−1^ (C=C, arom.); ^1^H-NMR (400 MHz, DMSO-*d*_6_): δ (ppm) = 2.40 (s, 6H, CH_3_), 6.90 (ddd, *J* = 8.0, 7.1, 1.0 Hz, 2H, ArH), 7.10 (ddd, *J* = 8.1, 7.1, 1.1 Hz, 2H, ArH), 7.20–7.25 (m, 2H, ArH), 7.39 (dt, *J* = 8.1, 0.9 Hz, 2H, ArH), 12.12 (s, 2H, NH); ^13^C-NMR (101 MHz, DMSO-*d*_6_): δ (ppm) = 13.8 (CH_3_), 104.8, 111.3, 120.4, 120.5, 121.9 (CH), 126.4, 135.9, 141.9, 180.1, 195.6 (C); C_22_H_16_N_2_O_2_ [340.38]; MS (EI) (*m*/*z*): 340 [M]^+^ (32), 284 [M^+^ − 56] (100); HRMS (EI) (*m*/*z*): Calcd. for [M]^+^ = 340.12063, found [M]^+^ = 340.12098; isocratic HPLC: 99.9% at 254 nm, 99.9% at 280 nm, t_ms_ = 3.02 min, t_m_ = 1.25 min (ACN/H_2_O 50:50); λ_max_ (nm): 275; gradient HPLC: 97.0% at 254 nm, t_ms_ = 11.2 min, t_m_ = 1.25 min.

##### 3,4-Bis(2-phenyl-1H-indol-3-yl)cyclobut-3-ene-1,2-dione (**2m**)

Synthesis according to General Procedure B (reaction time: 19 h) from squaric acid dichloride (**4**, 196 mg, 1.30 mmol) and 2-phenyl-1*H*-indole (567 mg, 2.93 mmol). Crystallization from ethyl acetate yieled an orange powder (72 mg, 12%). IR (KBr): 3350 cm^−1^ (NH), 1727 cm^−1^, 1711 cm^−1^ (C=O), 1548 cm^−1^,1520 cm^−1^ (C=C, arom.); ^1^H-NMR (400 MHz, DMSO-*d*_6_): δ (ppm) = 7.01 (ddd, *J* = 8.1, 7.1, 1.0 Hz, 2H), 7.05–7.22 (m, 8H), 7.26–7.32 (m, 4H), 7.36 (dt, *J* = 8.1, 0.9 Hz, 2H), 7.43 (d, *J* = 8.0 Hz, 2H), 12.12 (s, 2H, NH); ^13^C-NMR (100.7 MHz, DMSO-*d*_6_): δ (ppm) = 111.7, 120.8, 121.1, 122.7, 127.3, 128.2, 128.5 (CH), 104.2, 126.5, 131.0, 136.5, 141.2, 181.6, 196.0 (C); C_32_H_20_N_2_O_2_ [464.52]; MS (EI) (*m*/*z*): 464 [M]^+^ (33), 436 [M^+^ − 28] (12), 420 [M^+^ − 44] (32), 408 [M^+^ − 56] (100); HRMS (EI) (*m*/*z*): Calcd. for [M]^+^ = 464.15193, found [M]^+^ = 464.15250; isocratic HPLC: 97.4% at 254 nm, 99.3% at 280 nm, t_ms_ = 3.90 min, t_m_ = 1.03 min (ACN/H_2_O 60:40); λ_max_ (nm): 344, 297 and 238; gradient HPLC: 95.1% at 254 nm, t_ms_ = 12.4 min, t_m_ = 1.25 min.

##### 3,4-Bis(5-methoxy-1H-indol-3-yl)cyclobut-3-ene-1,2-dione (**2n**)

Synthesis according to General Procedure C from 3-chloro-4-(5-methoxy-1*H*-indol-3-yl)cyclobut-3-ene-1,2-dione (**5c**, 267 mg, 1.02 mmol) and 5-methoxy-1*H*-indole (315 mg, 2.14 mmol); yellow powder (Yield: 12%). IR (KBr): 3274 cm^−1^ (NH), 1748 cm^−1^, 1715 cm^−1^ (C=O), 1625 cm^−1^, 1586 cm^−1^,1533 cm^−1^ (C=C, arom.); ^1^H-NMR (400 MHz, DMSO-*d_6_*): δ (ppm) = 3.51 (s, 6H, CH_3_), 6.88 (ddd, *J* = 8.8, 2.5, 0.3 Hz, 2H, ArH), 7.31–7.35 (m, 2H, ArH), 7.46 (dd, *J* = 8.8, 0.5 Hz, 2H, ArH), 8.20 (d, *J* = 0.4 Hz, 2H, ArH), 12.32 (s, 2H, NH); ^13^C-NMR (151 MHz, DMSO-*d*_6_): δ (ppm) = 54.7 (CH_3_), 104.6, 112.8, 113.2, 131.4 (CH), 106.3, 125.7, 131.5, 154.4, 176.3, 194.6 (C); C_22_H_16_N_2_O_4_ [372.38]; Anal. calcd. for C_22_H_16_N_2_O_4_: C 70.96, H 4.33, N 7.52; found C 70.69, H 4.17, N 7.31; MS (EI): *m*/*z* (%) = 372 [M]^+^ (35), 316 [M^+^ − 56] (100); isocratic HPLC: 96.5% at 254 nm, 98.0% at 280 nm, t_ms_ = 3.28 min, t_m_ = 1.11 min (ACN/H_2_O 50:50); λ_max_ (nm): 277; gradient HPLC: 97.6% at 254 nm, t_ms_ = 10.9 min, t_m_ = 1.25 min.

##### 3,4-Bis(5-bromo-1H-indol-3-yl)cyclobut-3-ene-1,2-dione (**2o**)

Synthesis according to General Procedure D from 5-bromo-1*H*-indole (589 mg, 3.00 mmol) and squaric acid dichloride (**4**, 151 mg, 1.00 mmol). Crystallization from toluene/acetone yieled a tan powder (37 mg, 8%). IR (KBr): 3390 cm^−1^ (NH), 1749 cm^−1^, 1701 cm^−1^ (C=O); ^1^H-NMR (600 MHz, DMSO-*d_6_*): δ (ppm) = 7.42 (dd, *J* = 8.6, 2.0 Hz, 2H, ArH), 7.56 (dd, *J* = 8.6, 0.6 Hz, 2H, ArH), 8.20 (dd, *J* = 2.0, 0.5 Hz, 2H, ArH), 8.36 (s, 2H, ArH), 12.64 (s, 2H, NH), ^13^C-NMR (151 MHz, DMSO-*d_6_*): δ (ppm) = 114.7, 124.9, 125.7, 132.2 (CH), 105.9, 113.9, 126.9, 135.6, 176.7, 194.6 (C); C_20_H_10_Br_2_N_2_O_2_ [470.12]; Anal. calcd. for C_20_H_10_Br_2_N_2_O_2_: C 50.88, H 2.56, N 5.93; found C 50.53, H 2.14, N 5.62; MS (EI): *m*/*z* (%) = 468 [M]^+^ (12), 412 [M^+^ − 56] (100); isocratic HPLC: 97.6% at 254 nm and 98.4% at 280 nm, t_ms_ = 9.81 min, t_m_ = 1.11 min (ACN/H_2_O 50:50); λ_max_ (nm): 327, 278, 228; gradient HPLC: 98.1% at 254 nm, t_ms_ = 12.8 min, t_m_ = 1.25 min.

##### 3,3’-(3,4-Dioxocyclobut-1-ene-1,2-diyl)bis(1H-indole-5-carbonitrile) (**2p**) 

Synthesis according to General Procedure B (reaction time: 24 h) from squaric acid dichloride (**4**, 295 mg, 1.95 mmol) and 1*H*-indole-5-carbonitrile (570 mg, 4.01 mmol). Crystallization from chloroform/DMF yielded a yellow powder (59 mg, 12%). IR (KBr): 3232 cm^−1^ (NH), 2230 cm^−1^ (C≡N), 1764 cm^−1^, 1727 cm^−1^ (C=O), 1619 cm^−1^, 1556 cm^−1^, 1513 cm^−1^ (C=C, arom.); ^1^H-NMR (600 MHz, DMSO-*d_6_*): δ (ppm) = 7.68 (dd, *J* = 8.5, 1.7 Hz, 2H, ArH). 7.78 (dd, *J* = 8.5, 0.8 Hz, 2H, ArH), 8.46 (dt, *J* = 1.6, 0.7 Hz, 2H, ArH), 8.56 (d, *J* = 3.1 Hz, 2H, ArH), 12.95 (d, *J* = 3.6 Hz, 2H, NH); ^13^C-NMR (151 MHz, DMSO-*d_6_*): δ (ppm) = 114.6, 126.4, 128.3, 133.7 (CH), 104.0, 107.1, 120.5, 125.5, 139.2, 177.4, 195.1 (C); C_22_H_10_N_4_O_2_ [362.35]; MS (EI): *m*/*z* (%) = 362 [M]^+^∙(6), 206 [M^+^ - 56] (100); HRMS (EI) (*m*/*z*): Calcd. for [M]^+^ = 362.07983, found [M]^+^ = 362.07983; isocratic HPLC: 95.3% at 254 nm and 95.9% at 280 nm, t_ms_ = 5.11 min, t_m_ = 1.11 min (ACN/H_2_O 50:50); λ_max_ (nm): 239, 322 and 400; gradient HPLC: 96.9% at 254 nm, t_ms_ = 10.5 min, t_m_ = 1.25 min.

##### 3-(1H-Indol-3-yl)-4-(1-methyl-1H-indol-3-yl)cyclobut-3-ene-1,2-dione (**2q**)

Synthesis according to General Procedure C from 3-chloro-4-(1*H*-indol-3-yl)cyclobut-3-ene-1,2-dione (**5a**, 295 mg, 1.95 mmol) and 1-methyl-1*H*-indole (0.274 mL, 2.19 mmol) yielded a yellow powder (88 mg, 26%). IR (KBr): 3422 cm^−1^ (NH), 3171 cm^−1^, 1749 cm^−1^, 1713 cm^−1^ (C=O), 1616 cm^−1^, 1551 cm^−1^, 1524 cm^−1^ (C=C, arom.); ^1^H-NMR (400 MHz, DMSO-*d_6_*): δ (ppm) = 3.98 (s, 3H, CH_3_), 7.13–7.40 (m, 4H, ArH), 7.62 (ddt, *J* = 29.0, 8.2, 0.9 Hz, 2H, ArH), 7.89–8.03 (m, 2H, ArH), 8.24–8.30 (m, 1H, ArH), 8.35 (s, 1H, ArH), 12.44 (s, 1H, NH); ^13^C-NMR (101 MHz, DMSO-*d*_6_): δ (ppm) = 33.4 (CH_3_), 111.0, 112.5, 121.3, 121.5, 122.6, 122.8, 123.1, 131.0, 134.5 (CH), 105.3, 106.4, 125.2, 125.4, 136.7, 137.4, 176.3, 176.9, 194.6, 194.7 (C); C_21_H_14_N_2_O_2_ [326.36]; Anal. calcd. for C_21_H_14_N_2_O_2_: C 77.29, H 4.32, N 8.58; found C 76.80, H 4.17, N 8.21; MS (EI): *m*/*z* (%) = 231 [M]^+^ (35), 203 [M^+^ − 28] (4), 175 [M^+^ − 56] (100); isocratic HPLC: 99.3% at 254 nm, 99.4% at 280 nm, t_ms_ = 5.69 min, t_m_ = 1.11 min (ACN/H_2_O 50:50); λ_max_ (nm): 222, 272, 333; gradient HPLC: 98.1% at 254 nm, t_ms_ = 11.8 min, t_m_ = 1.25 min.

##### 3-(1H-Indol-3-yl)-4-(5-iodo-1H-indol-3-yl)cyclobut-3-ene-1,2-dione (**2r**)

Synthesis according to General Procedure C (reaction time: 20 h) from 3-chloro-4-(1*H*-indol-3-yl)cyclobut-3-ene-1,2-dione (**5a**, 234 mg, 1.01 mmol) and 5-iodo-1*H*-indole (360 mg, 1.48 mmol). Crystallization from ethyl acetate yielded a yellow powder (160 mg, 36%). IR (KBr): 3305 cm^−1^ (NH), 1751 cm^−1^, 1699 cm^−1^ (C=O), 1613 cm^−1^, 1537 cm^−1^, 1508 cm^−1^ (C=C, arom.); ^1^H-NMR (600 MHz, DMSO-*d_6_*): δ (ppm) = 7.19 (ddd, *J* = 8.0, 7.0, 1.0 Hz, 1H, ArH), 7.30 (ddd, *J* = 8.2, 7.1, 1.2 Hz, 1H, ArH), 7.43 (dd, *J* = 8.5, 0.6 Hz, 1H, ArH), 7.53–7.62 (m, 2H, ArH), 7.90 (dt, *J* = 8.0, 1.0 Hz, 1H, ArH), 8.22 (s, 1H), 8.33 (s, 1H, ArH), 8.39 (dd, *J* = 1.7, 0.5 Hz, 1H, ArH), 12.53 (s, 2H, NH); ^13^C-NMR (151 MHz, DMSO-*d_6_*): δ (ppm) = 112.7, 115.0, 121.4, 122.6, 123.2, 131.1, 131.1, 131.3, 131.6 (CH), 85.5, 105.7, 106.2, 124.9, 127.6, 135.9, 136.9, 176.4, 177.3, 194.7, 194.8 (C); C_20_H_11_IN_2_O_2_ [438.22]; Anal. calcd. for C_20_H_11_IN_2_O_2_: C 54.82, H 2.35, N 6.39; found C 54.93, H 2.49, N 6.25; MS (EI): *m*/*z* (%) = 438 [M]^+∙^(33), 382 [M^+^ − 56] (100); isocratic HPLC: 97.6% at 254 nm and 97.7% at 280 nm, t_ms_ = 6.47 min, t_m_ = 1.11 min (ACN/H_2_O 50:50); λ_max_ (nm): 273, 330 and 345; gradient HPLC: 97.9% at 254 nm, t_ms_ = 12.2 min, t_m_ = 1.25 min.

##### 3-(5-Bromo-1H-indol-3-yl)-4-(2-phenyl-1H-indol-3-yl)cyclobut-3-ene-1,2-dione (**2s**)

Synthesis according to General Procedure C (reaction time: 24 h) from 3-chloro-4-(2-phenyl-1*H*-indol-3-yl)cyclobut-3-ene-1,2-dione (**5d**, 309 mg, 1.00 mmol) and 5-bromo-1*H*-indole (297 mg, 1.52 mmol). Crystallization from ethyl acetate yielded an orange powder (130 mg, 28%). IR (KBr): 3253 cm^−1^ (NH), 1753 cm^−1^, 1712 cm^−1^ (C=O), 1615 cm^−1^, 1548 cm^−1^,1524 cm^−1^ (C=C, arom.); ^1^H-NMR (400 MHz, DMSO-*d_6_*): δ (ppm) = 7.07–7.39 (m, 7H, ArH), 7.42–7.51 (m, 1H, ArH), 7.51–7.62 (m, 3H, ArH), 7.76 (s, 1H, ArH), 7.93 (d, *J* = 13.7 Hz, 1H, ArH), 12.34 (s, 1H, NH), 12.51 (s, 1H, NH); ^13^C-NMR (101 MHz, DMSO-*d*_6_): δ (ppm) = 112.1, 114.3, 120.9, 121.5, 123.0, 124.6, 125.3, 128.2, 128.2, 128.6, 132.9 (CH), 102.8, 106.5, 113.9, 126.7, 128.3, 131.4, 135.2, 136.8, 140.0, 179.1, 179.19, 194.3, 196.5 (C); C_26_H_15_BrN_2_O_2_ [467.32]; Anal. calcd. for C_26_H_15_BrN_2_O_2_: C 66.82, H 3.24, N 5.99; found C 67.10, H 3.48, N 5.70; MS (EI): *m*/*z* (%) = 466 [M]^+^ (20), 438 [M^+^ − 28] (10), 410 [M^+^ − 56] (100); isocratic HPLC: 97.7% at 254 nm, 97.9% at 280 nm, t_ms_ = 3.77 min, t_m_ = 1.15 min (ACN/H_2_O 60:40); λ_max_ (nm): 230, 292; gradient HPLC: 95.7% at 254 nm, t_ms_ = 12.5 min, t_m_ = 1.25 min.

##### 3-(5-Bromo-1H-indol-3-yl)-4-(1-methyl-1H-indol-3-yl)cyclobut-3-ene-1,2-dione (**2t**)

Synthesis according to General Procedure C (reaction time: 22 h) from 3-chloro-4-(1-methyl-1*H*-indol-3-yl)cyclobut-3-ene-1,2-dione (**5b**, 189 mg, 0.77 mmol) and 5-bromo-1*H*-indole (221 mg, 1.13 mmol). Crystallization from dichloromethane/ethyl acetate/ethanol yielded a yellow powder (190 mg, 61%). IR (KBr): 3420 cm^−1^ (NH), 1749 cm^−1^, 1712 cm^−1^ (C=O), 1615 cm^−1^, 1548 cm^−1^,1521 cm^−1^ (C=C, arom.); ^1^H-NMR (400 MHz, DMSO-*d*_6_): δ (ppm) = 3.99 (s, 3H, CH_3_), 7.25 (ddd, *J* = 8.1, 7.1, 1.0 Hz, 1H, ArH), 7.32–7.45 (m, 2H, ArH), 7.55 (dd, *J* = 8.7, 0.5 Hz, 1H, ArH), 7.67 (dt, *J* = 8.3, 0.9 Hz, 1H, ArH), 7.89–7.99 (m, 1H, ArH), 8.26–8.32 (m, 2H, ArH), 8.44 (s, 1H), 12.58 (s, 1H, NH); ^13^C-NMR (101 MHz, DMSO-*d_6_*): δ (ppm) = 33.4 (CH_3_), 111.1, 114.5, 121.7, 122.8, 123.2, 124.9, 125.6, 132.0, 134.7 (CH), 105.1, 106.0, 113.8, 125.3, 127.1, 135.5, 137.5, 176.1, 176.6, 194.5, 194.6 (C); C_21_H_13_BrN_2_O_2_ [405.25]; MS (EI): *m*/*z* (%) = 404 [M]^+^ (23), 350 [M^+∙^-56] (100); HRMS (EI) (*m*/*z*): Calcd. for [M]^+^ = 404.01549, found [M]^+^ = 404.01552; isocratic HPLC: 99.4% at 254 nm, 99.7% at 280 nm, t_ms_ = 4.38 min, t_m_ = 1.15 min (ACN/H_2_O 60:40); λ_max_ (nm): 275, 328, and 344; gradient HPLC: 99.0% at 254 nm, t_ms_ = 12.8 min, t_m_ = 1.25 min.

##### 3-(1-Methyl-1H-indol-3-yl)-4-(2-phenyl-1H-indol-3-yl)cyclobut-3-ene-1,2-dione (**2u**)

Synthesis according to General Procedure D from 3-chloro-4-(2-phenyl-1*H*-indol-3-yl)cyclobut-3-ene-1,2-dione (**5d**, 310 mg, 1.01 mmol) and 1-methyl-1*H*-indole (190 mL, 1.52 mmol). Crystallization from petroleum ether/acetone yielded a yellow powder (105 mg, 26%). IR (KBr): 3438 cm^−1^ (NH), 1767 cm^−1^, 1732 cm^−1^ (C=O), 1616 cm^−1^, 1565 cm^−1^,1533 cm^−1^ (C=C, arom.); ^1^H-NMR (600 MHz, DMSO-*d*_6_): δ (ppm) = 3.70 (s, 3H, CH_3_), 7.06–7.13 (m, 2H, ArH), 7.18–7.30 (m, 6H, ArH), 7.47 (dt, *J* = 8.2, 0.9 Hz, 1H, ArH), 7.53–7.61 (m, 4H, ArH), 12.49 (s, 1H), 7.69 (s, 1H, ArH); ^13^C-NMR (151 MHz, DMSO-*d*_6_): δ (ppm) = 33.1 (CH_3_), 110.7, 112.0, 120.7, 121.4, 121.7, 122.3, 122.9, 122.9, 128.2, 128.2, 128.6, 135.7 (CH), 102.9, 105.7, 125.6, 126.8, 131.7, 136.7, 136.9, 139.1, 178.5, 178.9, 194.2, 196.5 (C); C_27_H_18_N_2_O_2_ [402.45]; C 80.58, H 6.96, N 4.51; found C 80.22, H 7.06, N 4.66; MS (EI): *m*/*z* (%) = 402 [M]^+^ (14), 374 [M^+^ − 28] (4), 346 [M^+^ − 56] (100); isocratic HPLC: 98.2% at 254 nm, 98.6% at 280 nm, t_ms_ = 4.05 min, t_m_ = 1.15 min (ACN/H_2_O 60:40); λ_max_ (nm): 297, 334; gradient HPLC: 98.1% at 254 nm, t_ms_ = 12.6 min, t_m_ = 1.25 min.

##### 3-(5-Methoxy-1H-indol-3-yl)-4-(2-methyl-1H-indol-3-yl)cyclobut-3-ene-1,2-dione (**2v**)

Synthesis according to General Procedure C (reaction time: 48 h) from 3-chloro-4-(2-methyl-1*H*-indol-3-yl)cyclobut-3-ene-1,2-dione (**5e**, 257 mg, 1.05 mmol) and 5-methoxy-1*H*-indole (149 mg, 1.01 mmol). Crystallization from ethanol/toluene yielded a yellow powder (70 mg, 20%). IR (KBr): 3191 cm^−1^ (NH), 1756 cm^−1^, 1710 cm^−1^ (C=O), 1622 cm^−1^, 1554 cm^−1^,1524 cm^−1^ (C=C, arom.); ^1^H-NMR (600 MHz, DMSO-*d*_6_): δ (ppm) = 2.57 (s, 3H, CH_3_), 3.32 (s, 3H, OCH_3_), 6.81 (dd, *J* = 8.8, 2.5 Hz, 1H, ArH), 6.94 (ddd, *J* = 8.0, 7.1, 1.0 Hz, 1H, ArH), 7.10–7.19 (m, 3H, ArH), 7.38 – 7.46 (m, 2H, ArH), 8.00 (s, 1H, ArH), 12.13 (s, 1H, NH), 12.29 (s, 1H, NH); ^13^C-NMR (151 MHz, DMSO-*d*_6_): δ (ppm) = 13.9, 54.4 (CH_3_), 104.2, 111.4, 113.0, 113.2, 120.3, 120.9, 121.8, 132.2 (CH), 104.0, 106.6, 125.7, 156.0, 131.4, 136.2, 140.9, 154.6, 177.8, 179.0, 194.4, 196.0 (C); C_22_H_16_N_2_O_3_ [356.38]; MS (EI): *m*/*z* (%) = 356 [M]^+^ (30), 300 [M^+^ − 56] (100); HRMS (EI) (*m*/*z*): Calcd. for [M]^+^ = 356.11554, found [M]^+^ = 356.11599; isocratic HPLC: 98.3% at 254 nm, 99.5% at 280 nm, t_ms_ = 3.52 min, t_m_ = 1.11 min (ACN/H_2_O 50:50), λ_max_: 276 nm; gradient HPLC: 95.4% at 254 nm, t_ms_ = 11.0 min, t_m_ = 1.25 min.

##### 3-(2-Methyl-1H-indol-3-yl)-4-(2-phenyl-1H-indol-3-yl)cyclobut-3-ene-1,2-dione (**2w**)

Synthesis according to General Procedure C (reaction time: 28 h) from 3-chloro-4-(2-methyl-1*H*-indol-3-yl)cyclobut-3-ene-1,2-dione (**5e**, 244 mg, 0.99 mmol) and 2-phenyl-1*H*-indole (403 mg, 3.02 mmol). Crystallization from acetone yielded a yellow powder (50 mg, 13%). IR (KBr): 3467 cm^−1^ (NH), 1764 cm^−1^, 1732 cm^−1^ (C=O), 1616 cm^−1^, 1552 cm^−1^,1522 cm^−1^ (C=C, arom.); ^1^H-NMR (400 MHz, DMSO-*d*_6_): δ (ppm) = 2.42 (s, 3H, CH_3_), 6.80 (ddd, *J* = 8.0, 7.1, 1.0 Hz, 1H, ArH), 6.92 (d, *J* = 8.0 Hz, 1H, ArH), 6.96–7.28 (m, 9H, ArH), 7.51 (dt, *J* = 8.1, 0.9 Hz, 1H, ArH), 7.63–7.70 (m, 1H, ArH), 11.85 (s, 1H, NH), 12.34 (s, 1H, NH); ^13^C-NMR (101 MHz, DMSO-*d*_6_): δ (ppm) = 13.7 (CH_3_), 111.0, 112.0, 120.2, 120.3, 120.9, 121.2, 121.7, 122.9, 127.1, 128.0, 128.1 (CH), 103.7, 105.6, 125.7, 127.2, 131.0, 136.1, 136.5, 141.2, 141.9, 180.4, 181.2, 195.2, 196.4 (C); C_27_H_18_N_2_O_2_ [402.45] MS (EI): *m*/*z* (%) = 402 [M]^+∙^ (22), 374 [M^+^ − 28] (100), 346 [M^+^ − 56] (100); HRMS (EI) (*m*/*z*): Calcd. for [M]^+^ = 402.13628, found [M]^+^ = 402.13693; isocratic HPLC: 98.8% at 254 nm, 98.8% at 280 nm, t_ms_ = 6.23 min, t_m_ = 1.11 min (ACN/H_2_O 50:50); λ_max_ (nm): 285, 233; gradient HPLC: 98.5% at 254 nm, t_ms_ = 11.9 min, t_m_ = 1.25 min.

##### 3-(5-Bromo-1H-indol-3-yl)-4-(2-methyl-1H-indol-3-yl)cyclobut-3-ene-1,2-dione (**2x**)

Synthesis according to General Procedure C (reaction time: 26 h) from 3-chloro-4-(2-methyl-1*H*-indol-3-yl)cyclobut-3-ene-1,2-dione (**5e**, 245 mg, 1.00 mmol) and 5-bromo-1*H*-indole (364 mg, 1.86 mmol). Crystallization from acetone yielded a yellow powder (70 mg, 17%). IR (KBr): 3387 cm^−1^ (NH), 1754 cm^−1^, 1714 cm^−1^ (C=O), 1615 cm^−1^, 1550 cm^−1^,1524 cm^−1^ (C=C, arom.); ^1^H-NMR (600 MHz, DMSO-*d*_6_): δ (ppm) = 2.59 (s, 3H, CH_3_), 7.00 (ddd, *J* = 8.0, 7.1, 1.0 Hz, 1H, ArH), 7.09 (dt, *J* = 7.9, 0.9 Hz, 1H, ArH), 7.18 (ddd, *J* = 8.1, 7.1, 1.2 Hz, 1H, ArH), 7.37 (dd, *J* = 8.6, 2.0 Hz, 1H, ArH), 7.44–7.54 (m, 2H, ArH), 7.83 (s, 1H, ArH), 8.14 (d, *J* = 1.9 Hz, 1H, ArH), 12.24 (s, 1H, NH), 12.50 (s, 1H, NH); ^13^C-NMR (151 MHz, DMSO-*d*_6_): δ (ppm) = 14.0 (CH_3_), 111.6, 114.5, 120.4, 121.1, 121.9, 124.6, 125.5, 133.0 (CH), 103.7, 106.2, 113.8, 124.9, 127.2, 135.2, 136.4, 141.6, 178.0, 178.7, 194.6, 195.6 (C); C_21_H_13_BrN_2_O_2_ [405.25]; Anal. calcd. for C_21_H_13_BrN_2_O_2_: C 62.24, H 3.23, N 6.91; found C 62.37, H 3.02, N 6.66; MS (EI): *m*/*z* (%) = 404 [M]^+^ (28), 348 [M^+^ − 56] (100); isocratic HPLC: 99.3% at 254 nm, 98.6% at 280 nm, t_ms_ = 6.00 min, t_m_ = 1.11 min (ACN/H_2_O 50:50); λ_max_ (nm): 276, 328, 334; gradient HPLC: 95.3% at 254 nm, t_ms_ = 11.7 min, t_m_ = 1.25 min.

##### 3-(1-Methyl-1H-indol-3-yl)-4-(2-methyl-1H-indol-3-yl)cyclobut-3-ene-1,2-dione (**2y**)

Synthesis according to General Procedure C (reaction time: 22 h) from 3-chloro-4-(2-methyl-1*H*-indol-3-yl)cyclobut-3-ene-1,2-dione (**5e**, 221 mg, 0.90 mmol) and 1-methyl-1*H*-indole (0.268 mL, 2.15 mmol) yielded a yellow powder (16 mg, 5%). IR (KBr): 3446 cm^−1^ (NH), 3295 cm^−1^ (CH, arom.), 1764 cm^−1^, 1735 cm^−1^ (C=O), 1611 cm^−1^, 1563 cm^−1^,1538 cm^−1^ (C=C, arom.); ^1^H-NMR (600 MHz, DMSO-*d*_6_): δ (ppm) = 2.51 (s, 3H, CH_3_), 3.91 (s, 3H, N-CH_3_), 6.97 (ddd, *J* = 8.1, 7.1, 1.0 Hz, 1H, ArH), 7.09 (ddd, *J* = 8.0, 7.1, 1.0 Hz, 1H, ArH), 7.16 (ddd, *J* = 8.1, 7.1, 1.1 Hz, 1H, ArH), 7.21 (dt, *J* = 7.9, 0.9 Hz, 1H, ArH), 7.29 (ddd, *J* = 8.2, 7.1, 1.1 Hz, 1H, ArH), 7.45 (dt, *J* = 8.1, 0.9 Hz, 1H, ArH), 7.60 (dt, *J* = 8.3, 0.9 Hz, 1H, ArH), 7.72 (d, *J* = 8.0 Hz, 1H, ArH), 8.07 (s, 1H, ArH), 12.17 (s, 1H, NH); ^13^C-NMR (151 MHz, DMSO-*d*_6_): δ (ppm) = 14.0 (CH_3_), 33.3 (N-CH_3_), 111.0, 111.5, 120.3, 121.0, 121.6, 121.9, 123.0, 135.4 (CH), 104.0, 105.5, 122.2, 125.6, 125.7, 136.3, 137.2, 141.0, 177.9, 178.2, 194.57, 195.7 (C); C_22_H_16_N_2_O_2_ [340.38]; MS (EI): *m*/*z* (%) = 340 [M]^+^ (34), 284 [M^+^-56] (100); HRMS (EI) (*m*/*z*): Calcd. for [M]^+^ = 340.12063, found [M]^+^ = 340.10261; isocratic HPLC: 99.6% at 254 nm, 99.7% at 280 nm, t_ms_ = 6.56 min, t_m_ (DMSO)= 1.11 min (ACN/H_2_O 50:50); λ_max_ (nm): 274; gradient HPLC: 97.3% at 254 nm, t_ms_ = 11.9 min, t_m_ = 1.25 min.

##### 3-(5-Bromo-1-methyl-1H-indol-3-yl)-4-(2-methyl-1H-indol-3-yl)cyclobut-3-ene-1,2-dione (**2z**)

Synthesis according to General Procedure D from 3-chloro-4-(2-methyl-1*H*-indol-3-yl)cyclobut-3-ene-1,2-dione (**5e**, 246 mg, 1.02 mmol) and 5-bromo-1-methyl-1*H*-indole (316 mg, 1.50 mmol). Crystallization from ethanol/toluene yielded an orange powder (138 mg, 32%). IR (KBr): 3422 cm^−1^ (NH), 1767 cm^−1^, 1716 cm^−1^ (C=O), 1607 cm^−1^, 1557 cm^−1^,1529 cm^−1^ (C=C, arom.); ^1^H-NMR (400 MHz, DMSO-*d*_6_): δ (ppm) = 2.56 (s, 3H, CH_3_), 3.88 (s, 3H, N-CH_3_), 6.99 (ddd, *J* = 8.0, 7.0, 1.1 Hz, 1H, ArH), 7.11–7.24 (m, 2H, ArH), 7.37–7.50 (m, 2H, ArH), 7.59 (d, *J* = 8.7 Hz, 1H, ArH), 7.98 (s, 1H, ArH), 8.01 (d, *J* = 1.9 Hz, 1H, ArH), 12.23 (s, 1H, NH); ^13^C-NMR (101 MHz, DMSO-*d*_6_): δ (ppm) = 14.2 (CH_3_), 33.5 (N-CH_3_), 111.6, 113.2, 120.4, 121.1, 122.1, 124.8, 125.4, 136.3 (CH), 103.9, 105.2, 114.3, 125.5, 127.5, 136.1, 136.5, 141.4, 177.4, 178.6, 194.6, 195.6 (C); C_22_H_15_BrN_2_O_2_ [419.28]; C 63.02, H 3.61, N 6.68; found C 63.15, H 3.58, N 6.34; MS (EI): *m*/*z* (%) = 418 [M]^+^ (25), 362 [M^+^ − 56] (100); isocratic HPLC: 96.2% at 254 nm, 98.1% at 280 nm, t_ms_ = 4.48 min, t_m_ = 1.15 min (ACN/H_2_O 60:40); λ_max_ (nm): 277, 334; gradient HPLC: 95.6% at 254 nm, t_ms_ = 12.9 min, t_m_ = 1.25 min.

##### 3-(5-Methoxy-1H-indol-3-yl)-4-(2-phenyl-1H-indol-3-yl)cyclobut-3-ene-1,2-dione (**2aa**)

Synthesis according to General Procedure C (reaction time: 65 h) from 3-chloro-4-(5-methoxy-1*H*-indol-3-yl)cyclobut-3-ene-1,2-dione (**5c**, 225 mg, 0.86 mmol) and 2-phenyl-1*H*-indole (272 mg, 1.42 mmol). Crystallization from ethanol/toluene yielded a yellow powder (62 mg, 17%). IR (KBr): 3257 cm^−1^ (NH), 1751 cm^−1^, 1708 cm^−1^ (C=O), 1624 cm^−1^, 1547 cm^−1^,1523 cm^−1^ (C=C, arom.); ^1^H-NMR (600 MHz, DMSO-*d*_6_): δ (ppm) = 3.32 (s, 3H, O-CH_3_), 6.75 (dd, *J* = 8.8, 2.5 Hz, 1H), 7.03 (ddd, *J* = 8.0, 6.9, 1.0 Hz, 1H, ArH), 7.21–7.36 (m, 6H, ArH), 7.38–7.43 (m, 1H, ArH), 7.53–7.62 (m, 4H, ArH), 12.22 (s, 1H, NH), 12.42 (s, 1H, NH); ^13^C-NMR (151 MHz, DMSO-*d*_6_): δ (ppm) = 54.4 (CH_3_), 111.9, 112.8, 113.1, 120.7, 121.1, 122.9, 127.4, 128.0, 128.4, 128.5, 131.3 (CH), 102.8, 104.1, 106.9, 125.8, 131.4, 132.3, 136.6, 139.3, 154.7, 178.3, 180.7, 193.9, 197.2 (C); C_27_H_18_N_2_O_3_ [418.45]; MS (EI): *m*/*z* (%) = 418 [M]^+∙^ (17), 390 [M^+^ − 56] (6), 362 [M^+^ − 56] (100); HRMS (EI) (*m*/*z*): Calcd. for [M]^+^ = 418.13119, found [M]^+^ = 418.13126; isocratic HPLC: 97.1% at 254 nm, 97.2% at 280 nm, t_ms_ = 5.21 min, t_m_ = 1.11 min (ACN/H_2_O 50:50); λ_max_ (nm): 294, 344, 377; gradient HPLC: 95.1% at 254 nm, t_ms_ = 11.6 min, t_m_ = 1.25 min.

##### 3-(5-Fluoro-1H-indol-3-yl)-4-(5-methoxy-1H-indol-3-yl)cyclobut-3-ene-1,2-dione (**2ab**)

Synthesis according to General Procedure C (reaction time: 20 h) from 3-chloro-4-(5-methoxy-1*H*-indol-3-yl)cyclobut-3-ene-1,2-dione (**5c**, 260 mg, 0.99 mmol) and 5-fluoro-1*H*-indole (201 mg, 1.49 mmol). Crystallization from ethanol/DMF yielded a tan powder (184 mg, 51%). IR (KBr): 3443 cm^−1^ (NH), 1747 cm^−1^, 1712 cm^−1^ (C=O), 1629 cm^−1^, 1538 cm^−1^ (C=C, arom.); ^1^H-NMR (600 MHz, DMSO-*d_6_*): δ (ppm) = 3.58 (s, 3H, OCH_3_), 6.91 (dd, *J* = 8.8, 2.5 Hz, 1H, ArH), 7.14 (td, *J* = 9.1, 2.6 Hz, 1H, ArH), 7.31–7.37 (m, 1H, ArH), 7.48 (dd, *J* = 8.8, 0.5 Hz, 1H, ArH), 7.59 (ddd, *J* = 8.8, 4.7, 0.5 Hz, 1H, ArH), 7.75 (dd, *J* = 10.1, 2.6 Hz, 1H, ArH), 8.24 (d, *J* = 3.1 Hz, 1H, ArH), 8.31 (d, *J* = 3.2 Hz, 1H, ArH), 12.39 (d, *J* = 3.3 Hz, 1H, NH), 12.50 (d, *J* = 3.1 Hz, 1H, NH); ^13^C-NMR (151 MHz, DMSO-*d_6_*): δ (ppm) = 55.0 (O-CH_3_), 104.8, 107.6 (d, ^2^*J*_C,F_ = 24.9 Hz), 111.3 (d, ^2^*J*_C,F_ = 26.0 Hz), 113.0, 113.5, 113.8 (d, ^3^*J*_C,F_ = 10.0 Hz), 113.9, 132.6 (CH), 106.2, 106.7 (d, ^4^*J*_C,F_ = 4.2 Hz), 113.8, 125.6, 126.0 (d, ^3^*J*_C,F_ = 10.9 Hz), 133.4, 154.7, 157.8 (d, ^1^*J*_C,F_ = 234.4 Hz), 175.9, 177.0, 194.5, 194.9 (C); C_21_H_13_FN_2_O_2_ [360.34]; MS (EI): *m*/*z* (%) = 360 [M]^+^ (35), 304 [M^+∙^-56] (100); HRMS (EI) (*m*/*z*): Calcd. for [M]^+^ = 360.09047, found [M]^+^ = 360.09119; isocratic HPLC: 97.2% at 254 nm and 98.2% at 280 nm, t_ms_ = 3.68 min, t_m_ = 1.11 min (ACN/H_2_O 50:50); λ_max_ (nm): 276; gradient HPLC: 98.4% at 254 nm, t_ms_ = 11.1 min, t_m_ = 1.25 min.

##### 3-(5-Chloro-1H-indol-3-yl)-4-(5-methoxy-1H-indol-3-yl)cyclobut-3-ene-1,2-dione (**2ac**)

Synthesis according to General Procedure C (reaction time: 24 h) from 3-chloro-4-(5-methoxy-1*H*-indol-3-yl)cyclobut-3-ene-1,2-dione (**5c**, 262 mg, 1.00 mmol) and 5-chloro-1*H*-indole (220 mg, 1.45 mmol). The dark green precipitate was crystallized from ethanol/toluene to yield a yellow powder (195 mg, 52%). IR (KBr): 3428 cm^−1^ (NH), 1750 cm^−1^, 1715 cm^−1^ (C=O), 1624 cm^−1^, 1540 cm^−1^, 1517 cm^−1^ (C=C, arom.); ^1^H-NMR (400 MHz, DMSO-*d*_6_): δ (ppm) = 3.59 (s, 3H, O-CH_3_), 6.93 (dd, *J* = 8.8, 2.5 Hz, 1H, ArH), 7.26–7.35 (m, 2H, ArH), 7.50 (d, *J* = 8.8 Hz, 1H, ArH), 7.61 (dd, *J* = 8.6, 0.5 Hz, 1H, ArH), 8.07 (d, *J* = 2.1 Hz, 1H, ArH), 8.24 (d, *J* = 3.1 Hz, 1H, ArH), 8.33 (d, *J* = 3.2 Hz, 1H, ArH), 12.41 (d, *J* = 3.1 Hz, 1H, NH), 12.55 (d, *J* = 3.1 Hz, 1H, NH); ^13^C-NMR (101 MHz, DMSO-*d*_6_): δ (ppm) = 55.0 (O-CH_3_), 104.9, 113.0, 113.5, 114.1, 121.8, 123.0, 131.6, 132.2 (CH), 106.2, 106.2, 125.5, 125.7, 126.5, 131.7, 135.2, 154.8, 175.7, 177.2, 194.4, 195.0 (C); C_21_H_13_ClN_2_O_3_ [376.80]; Anal. calcd. for C_21_H_13_ClN_2_O_3_: C 66.94, H 3.48, N 7.43; found C 66.65, H 3.38, N 7.13; MS (EI): *m*/*z* (%) = 376 [M]^+^ (28), 320 [M^+∙^-56] (100); isocratic HPLC: 98.3% at 254 nm and 98.9% at 280 nm, t_ms_ = 4.87 min, t_m_ = 1.11 min (ACN/H_2_O 50:50); λ_max_ (nm): 277; gradient HPLC: 95.6% at 254 nm, t_ms_ = 11.5 min, t_m_ = 1.25 min.

##### 3-(5-Bromo-1H-indol-3-yl)-4-(5-methoxy-1H-indol-3-yl)cyclobut-3-ene-1,2-dione (**2ad**)

Synthesis according to General Procedure F from 3-chloro-4-(5-methoxy-1*H*-indol-3-yl)cyclobut-3-ene-1,2-dione (**5c**, 262 mg, 1.00 mmol) and 5-bromo-1*H*-indole (585 mg, 2.98 mmol). Crystallization from petroleum ether/ethyl acetate yielded a yellow powder (84 mg, 20%). IR (KBr): 3418 cm^−1^ (NH), 1746 cm^−1^, 1700 cm^−1^ (C=O), 1530 cm^−1^ (C=C, arom.); ^1^H-NMR (600 MHz, DMSO-*d*_6_): δ (ppm) = 3.58 (s, 3H, CH_3_), 6.92 (ddd, *J* = 8.8, 2.5, 0.4 Hz, 1H, ArH), 7.28–7.34 (m, 1H, ArH), 7.40 (dd, *J* = 8.6, 2.0 Hz, 1H, ArH), 7.48 (dd, *J* = 8.8, 0.5 Hz, 1H, ArH), 7.55 (dd, *J* = 8.6, 0.5 Hz, 1H, ArH), 8.19–8.23 (m, 2H, ArH), 8.32 (d, *J* = 0.4 Hz, 1H, ArH), 12.45 (s, 2H, NH); ^13^C-NMR (151 MHz, DMSO-*d*_6_): δ (ppm) = 54.9 (CH_3_), 104.7, 112.9, 113.4, 114.5, 124.8, 125.5, 131.54, 132.0 (CH), 105.9, 106.1, 113.6, 125.4, 127.3, 131.6, 135.4, 154.7, 175.5, 177.1, 194.3, 194.9 (C); C_21_H_13_BrN_2_O_2_ [421.25]; Anal. calcd. for C_21_H_13_BrN_2_O_2_: C 59.88, H 3.11, N 6.65; found C 59.83, H 3.04, N 6.40; MS (EI): *m*/*z* (%) = 422 [M]^+^ (25), 366 [M^+^ − 56] (100); isocratic HPLC: 99.4% at 254 nm, 99.7% at 280 nm, t_ms_ = 5.24 min, t_m_ = 1.11 min (ACN/H_2_O 50:50); λ_max_ (nm): 278; gradient HPLC: 99.4% at 254 nm, t_ms_ = 11.8 min, t_m_ = 1.25 min.

##### 3-(5-Iodo-1H-indol-3-yl)-4-(5-methoxy-1H-indol-3-yl)cyclobut-3-en-1,2-dione (**2ae**)

Synthesis according to General Procedure C (reaction time: 24 h) from 3-chloro-4-(5-methoxy-1*H*-indol-3-yl)cyclobut-3-ene-1,2-dione (**5c**, 262 mg, 1.00 mmol) and 5-iodo-1*H*-indole (362 mg, 1.45 mmol). The green precipitate crystallized from ethanol/toluene to yield a tan powder (200 mg, 43%). IR (KBr): 3400 cm^−1^ (NH), 1750 cm^−1^, 1703 cm^−1^ (C=O), 1626 cm^−1^, 1539 cm^−1^, 1512 cm^−1^ (C=C, arom.); ^1^H-NMR (400 MHz, DMSO-*d*_6_): δ (ppm) = 3.57 (s, 3H, O-CH_3_), 6.93 (dd, *J* = 8.8, 2.5 Hz, 1H, ArH), 7.30 (d, *J* = 2.5 Hz, 1H, ArH), 7.44 (dd, *J* = 8.6, 0.6 Hz, 1H, ArH), 7.47–7.60 (m, 2H, ArH), 8.17 (d, *J* = 3.0 Hz, 1H), 8.31 (d, *J* = 3.2 Hz, 1H, ArH), 8.37 (d, *J* = 1.7 Hz, 1H, ArH), 12.40 (d, *J* = 3.2 Hz, 1H, NH), 12.52 (d, *J* = 3.0 Hz, 1H, NH); ^13^C-NMR (101 MHz, DMSO-*d*_6_): δ (ppm) = 54.9 (O-CH_3_), 104.9, 113.0, 113.4, 114.9, 131.0, 131.1, 131.6 (2C) (CH), 85.3, 105.7, 106.2, 125.5, 127.7, 131.7, 135.8, 154.7, 175.7, 177.2, 194.4, 195.0 (C); C_21_H_13_IN_2_O_3_ [468.25]; MS (EI): *m*/*z* (%) = 468 [M]^+^ (33), 412 [M^+^ − 56] (100); HRMS (EI) (*m*/*z*): Calcd. for [M]^+^ = 467.99654, found [M]^+∙^= 467.99523; isocratic HPLC: 95.6% at 254 nm and 96.8% at 280 nm, t_ms_ = 6.01 min, t_m_ = 1.11 min (ACN/H_2_O 50:50); λ_max_ (nm): 256, 309; gradient HPLC: 95.6% at 254 nm, t_ms_ = 12.0 min, t_m_ = 1.25 min.

##### 3-(5-Methoxy-1H-indol-3-yl)-4-(1-methyl-1H-indol-3-yl)cyclobut-3-ene-1,2-dione (**2af**)

Synthesis according to General Procedure C (reaction time: 65 h) from 3-chloro-4-(5-methoxy-1*H*-indol-3-yl)cyclobut-3-ene-1,2-dione (**5c**, 245 mg, 0.90 mmol) and 1-methyl-1*H*-indole (0.190 mL, 1.52 mmol) yielded a yellow powder (56 mg, 18%). IR (KBr): 3416 cm^−1^ (NH), 1751 cm^−1^, 1708 cm^−1^ (C=O), 1624 cm^−1^, 1530 cm^−1^,1514 cm^−1^ (C=C, arom.); ^1^H-NMR (600 MHz, DMSO-*d*_6_): δ (ppm) = 3.55 (s, 3H, N-CH_3_), 3.98 (s, 3H, O-CH_3_), 6.90 (dd, *J* = 8.8, 2.5 Hz, 1H, ArH), 7.20 (ddd, *J* = 8.0, 7.0, 1.0 Hz, 1H, ArH), 7.34 (ddd, *J* = 8.2, 7.1, 1.1 Hz, 1H, ArH), 7.41 - 7.44 (m, 1H, ArH), 7.47 (dd, *J* = 8.8, 0.5 Hz, 1H, ArH), 7.65 (dt, *J* = 8.3, 0.9 Hz, 1H, ArH), 7.92 (dt, *J* = 8.0, 1.0 Hz, 1H), 8.25 (s, 1H, ArH), 8.32 (s, 1H, ArH), 12.35 (s, 1H, NH); ^13^C-NMR (151 MHz, DMSO-*d*_6_): δ (ppm) = 33.3 (N-CH_3_), 54.8 (O-CH_3_), 104.7, 110.9, 112.9, 113.2, 121.4, 122.7, 123.0, 131.3, 134.3 (CH), 105.2, 106.3, 125.4, 125.8, 131.5, 137.3, 154.6, 175.6, 176.8, 194.3, 194.8 (C); C_22_H_16_N_2_O_3_ [356.38] C 74.15, H 4.53, N 7.86; found C 74.01, H 4.44, N 7.61; MS (EI): *m*/*z* (%) = 356 [M]^+^ (30), 300 [M^+^ − 56] (100); isocratic HPLC: 99.6% at 254 nm, 99.7% at 280 nm, t_ms_ = 5.12 min, t_m_ = 1.11 min (ACN/H_2_O 50:50); λ_max_ (nm): 275, 351; gradient HPLC: 97.3% at 254 nm, t_ms_ = 11.8 min, t_m_ = 1.25 min.

##### 3-(5-Bromo-1-methyl-1H-indol-3-yl)-4-(5-methoxy-1H-indol-3-yl)cyclobut-3-ene-1,2-dione (**2ag**)

Synthesis according to General Procedure C (reaction time: 20 h) from 3-chloro-4-(5-methoxy-1*H*-indol-3-yl)cyclobut-3-ene-1,2-dione (**5c**, 265 mg, 1.01 mmol) and 5-bromo-1-methyl-1*H*-indole (319 mg, 1.52 mmol). The yellow precipitate crystallized from ethanol/toluene to yield a yellow powder (215 mg, 49%). IR (KBr): 3400 cm^−1^ (NH), 1751 cm^−1^, 1714 cm^−1^ (C=O), 1623 cm^−1^, 1609 cm^−1^,1547 cm^−1^, 1519 cm^−1^ (C=C, arom.); ^1^H-NMR (600 MHz, DMSO-*d*_6_): δ (ppm) = 3.60 (s, 3H, CH_3_), 3.97 (s, 3H, O-CH_3_), 6.92 (dd, *J* = 8.8, 2.5 Hz, 1H, ArH), 7.43 (d, *J* = 2.4 Hz, 1H, ArH), 7.45–7.50 (m, 2H, ArH), 7.63 (dd, *J* = 8.7, 0.5 Hz, 1H, ArH), 8.21 (dd, *J* = 2.0, 0.6 Hz, 1H, ArH), 8.31–8.37 (m, 2H, ArH), 12.41 (s, 1H, NH); ^13^C-NMR (151 MHz, DMSO-*d*_6_): δ (ppm) = 34.1 (CH_3_), 55.5 (O-CH_3_), 105.3, 113.6, 113.9, 125.6, 126.0, 132.0, 135.7 (CH), 105.5, 106.8, 114.7, 126.3, 127.9, 132.2, 136.6, 155.4, 175.4, 177.6, 194.7, 195.3 (C); C_22_H_15_BrN_2_O_3_ [435.28] MS (EI): *m*/*z* (%) = 434 [M]^+^ (27), 406 [M^+^ − 28] (1), 378 [M^+^ − 56] (100); HRMS (EI) (*m*/*z*): Calcd. for [M]^+^ = 434.02606, found [M]^+^ = 434.02622; isocratic HPLC: 97.9% at 254 nm, 98.0% at 280 nm, t_ms_ = 3.83 min, t_m_ = 1.15 min (ACN/H_2_O 60:40); λ_max_ (nm): 279, 349; gradient HPLC: 98.7% at 254 nm, t_ms_ = 12.5 min, t_m_ = 1.25 min.

##### 3-[2-(5-Methoxy-1H-indol-3-yl)-3,4-dioxocyclobut-1-en-1-yl]-1H-indole-5-carbonitrile (**2ah**)

Synthesis according to General Procedure C (reaction time: 24 h) from 3-chloro-4-(5-methoxy-1*H*-indol-3-yl)cyclobut-3-ene-1,2-dione (**5c**, 258 mg, 0.99 mmol) and 1*H*-indole-5-carbonitrile (220 mg, 1.55 mmol). The yellow precipitate was crystallized from ethanol/toluene to yield a yellow powder (202 mg, 56%). IR (KBr): 3370 cm^−1^ (NH), 2220 cm^−1^ (C≡N), 1752 cm^−1^, 1719 cm^−1^ (C=O), 1620 cm^−1^, 1553 cm^−1^, 1520 cm^−1^ (C=C, arom.); ^1^H-NMR (400 MHz, DMSO-*d*_6_): δ (ppm) = 3.58 (s, 3H, CH_3_), 6.94 (dd, *J* = 8.8, 2.5 Hz, 1H, ArH), 7.28 (d, *J* = 2.5 Hz, 1H, ArH), 7.45–7.55 (m, 1H, ArH), 7.65 (dd, *J* = 8.5, 1.6 Hz, 1H, ArH), 7.77 (dd, *J* = 8.4, 0.8 Hz, 1H, ArH), 8.37 (dd, *J* = 20.9, 3.1 Hz, 2H, ArH), 8.47 (dd, *J* = 1.5, 0.7 Hz, 1H, ArH), 12.47 (d, *J* = 3.2 Hz, 1H, NH), 12.82 (d, *J* = 2.9 Hz, 1H, NH); ^13^C-NMR (101 MHz, DMSO-*d*_6_): δ (ppm) = 55.0 (O-CH_3_), 104.9, 113.0, 113.6, 114.0, 125.6, 127.1, 132.0, 132.8 (CH), 103.2, 106.2, 106.9, 120.1, 125.3, 125.5, 131.8, 138.5, 154.9, 174.9, 177.9, 194.2, 195.2 (C); C_21_H_13_N_3_O_3_ [367.36]; MS (EI): *m*/*z* (%) = 367 [M]^+^ (25), 311 [M^+^ − 56] (100); HRMS (EI) (*m*/*z*): Calcd. for [M]^+∙^= 367.09514, found [M]^+^ = 367.09491; isocratic HPLC: 97.5% at 254 nm and 97.5% at 280 nm, t_ms_ = 5.79 min, t_m_ = 1.11 min (ACN/H_2_O 40:60); λ_max_ (nm): 266, 308; gradient HPLC: 96.0% at 254 nm, t_ms_ = 10.4 min, t_m_ = 1.25 min.

##### 3-(7-Chloro-1H-indol-3-yl)-4-(5-methoxy-1H-indol-3-yl)cyclobut-3-ene-1,2-dione (**2ai**)

Synthesis according to General Procedure C (reaction time: 48 h) from 3-chloro-4-(5-methoxy-1*H*-indol-3-yl)cyclobut-3-ene-1,2-dione (**5c**, 267 mg, 1.02 mmol) and 7-chloro-1*H*-indole (228 mg, 1.50 mmol). The yellow precipitate recrystallized from ethanol/toluene to yield a yellow powder (199 mg, 52%). IR (KBr): 3420 cm^−1^, 3383 cm^−1^ (NH), 1754 cm^−1^, 1718 cm^−1^ (C=O), 1620 cm^−1^, 1586 cm^−1^,1540 cm^−1^, 1513 cm^−1^ (C=C, arom.); ^1^H-NMR (600 MHz, DMSO-*d*_6_): δ (ppm) = 3.54 (s, 3H, CH_3_), 6.91 (dd, *J* = 8.8, 2.5 Hz, 1H, ArH), 7.17 (t, *J* = 7.9 Hz, 1H, ArH), 7.23 (d, *J* = 2.4 Hz, 1H, ArH), 7.37 (dd, *J* = 7.6, 0.9 Hz, 1H, ArH), 7.48 (d, *J* = 8.9 Hz, 1H, ArH), 7.90 (dt, *J* = 7.9, 0.8 Hz, 1H, ArH), 8.11 (d, *J* = 3.0 Hz, 1H, ArH), 8.30 (d, *J* = 3.2 Hz, 1H, ArH), 12.42 (d, *J* = 3.2 Hz, 1H, NH), 12.79 (d, *J* = 3.0 Hz, 1H, NH); ^13^C-NMR (151 MHz, DMSO-*d*_6_): δ (ppm) = 54.9 (CH_3_), 104.8, 113.0, 113.6, 121.5, 122.2, 122.6, 131.3, 132.0 (CH) 106.2, 107.4, 116.8, 125.5, 127.2, 131.8, 133.6, 154.8, 175.6, 177.9, 194.1, 195.4 (C); C_21_H_13_ClN_2_O_3_ [376.80]; C 66.94, H 3.48, N 7.43; found C 67.05, H 3.36, N 7.37; MS (EI): *m*/*z* (%) = 376 [M]^+^ (32), 320 [M^+^ − 56] (100); isocratic HPLC: 97.5% at 254 nm, 98.0% at 280 nm, t_ms_ = 5.69 min, t_m_ = 1.15 min (ACN/H_2_O 50:50); λ_max_ (nm): 274; gradient HPLC: 97.8% at 254 nm, t_ms_ = 11.8 min, t_m_ = 1.25 min.

##### 3-(7-Bromo-1H-indol-3-yl)-4-(5-methoxy-1H-indol-3-yl)cyclobut-3-ene-1,2-dione (**2aj**)

Synthesis according to General Procedure D from 3-chloro-4-(5-methoxy-1*H*-indol-3-yl)cyclobut-3-ene-1,2-dione (**5c**, 270 mg, 1.03 mmol) and 7-bromo-1*H*-indole (294 mg, 1.50 mmol). Crystallization from ethanol/toluene yielded a yellow powder (162 mg, 37%). IR (KBr): 3325 cm^−1^ (NH), 1749 cm^−1^, 1708 cm^−1^ (C=O), 1619 cm^−1^, 1536 cm^−1^,1514 cm^−1^ (C=C, arom.); ^1^H-NMR (600 MHz, DMSO-*d*_6_): δ (ppm) = 3.54 (s, 3H, CH_3_), 6.91 (dd, *J* = 8.8, 2.5 Hz, 1H, ArH), 7.12 (t, *J* = 7.9 Hz, 1H, ArH), 7.22 (d, *J* = 2.4 Hz, 1H, ArH), 7.46–7.54 (m, 2H, ArH), 7.93 (dt, *J* = 8.0, 0.8 Hz, 1H, ArH), 8.08 (d, *J* = 3.2 Hz, 1H, ArH), 8.30 (d, *J* = 3.2 Hz, 1H, ArH), 12.43 (d, *J* = 3.2 Hz, 1H, NH), 12.63 (d, *J* = 3.1 Hz, 1H, NH); ^13^C-NMR (151 MHz, DMSO-*d*_6_): δ (ppm) = 54.9 (CH_3_), 104.8, 113.0, 113.6, 121.9, 122.5, 125.7, 131.2, 132.0 (CH), 104.9, 106.2, 107.5, 125.4, 126.9, 131.8, 135.1, 154.8, 175.7, 177.9, 194.1, 195.4 (C); C_21_H_13_BrN_2_O_3_ [421.25]; C 59.88, H 3.11, N 6.65; found C 59.92, H 3.01, N 6.65; MS (EI): *m*/*z* (%) = 420 [M]^+∙^ (26), 364 [M^+^ − 56] (100); isocratic HPLC: 97.7% at 254 nm, 98.5% at 280 nm, t_ms_ = 5.69 min, t_m_ = 1.15 min (ACN/H_2_O 50:50); λ_max_ (nm): 275; gradient HPLC: 98.2% at 254 nm, t_ms_ = 12.0 min, t_m_ = 1.25 min.

##### 3-(7-Iodo-1H-indol-3-yl)-4-(5-methoxy-1H-indol-3-yl)cyclobut-3-ene-1,2-dione (**2ak**)

Synthesis according to General Procedure D from 3-chloro-4-(5-methoxy-1*H*-indol-3-yl)cyclobut-3-ene-1,2-dione (5**c**, 263 mg, 1.01 mmol) and 7-iodo-1*H*-indole (244 mg, 1.00 mmol). Crystallization from acetone yielded a yellow powder (111 mg, 24%). IR (KBr): 3413 cm^−1^ (NH), 1754 cm^−1^, 1721 cm^−1^ (C=O), 1608 cm^−1^, 1544 cm^−1^,1512 cm^−1^ (C=C, arom.); ^1^H-NMR (600 MHz, DMSO-*d*_6_): δ (ppm) = 3.53 (s, 3H, CH_3_), 6.90 (dd, *J* = 8.8, 2.5 Hz, 1H, ArH), 6.93–6.99 (m, 1H, ArH), 7.21 (d, *J* = 2.4 Hz, 1H, ArH), 7.48 (d, *J* = 8.8 Hz, 1H, ArH), 7.68 (dd, *J* = 7.4, 1.0 Hz, 1H, ArH), 7.92 (dt, *J* = 8.0, 0.9 Hz, 1H, ArH), 8.04 (d, *J* = 3.2 Hz, 1H, ArH), 8.28 (d, *J* = 3.2 Hz, 1H, ArH), 12.33 (d, *J* = 3.1 Hz, 1H, ArH), 12.42 (d, *J* = 3.1 Hz, 1H, NH); ^13^C-NMR (151 MHz, DMSO-*d*_6_): δ (ppm) = 54.9 (CH_3_), 104.8, 113.0, 113.6, 122.5, 122.9, 131.0, 131.8, 132.1 (CH), 77.8, 106.2, 107.6, 125.4, 125.8, 131.9, 138.6, 154.8, 175.9, 177.8, 194.2, 195.4 (C); C_21_H_13_IN_2_O_3_ [468.25]; C 53.87, H 2.80, N 5.98; found C 54.00, H 2.58, N 5.78; MS (EI): *m*/*z* (%) = 468 [M]^+∙^ (33), 364 [M^+^ − 56] (100); isocratic HPLC: 99.4% at 254 nm, 99.8% at 280 nm, t_ms_ = 7.13 min, t_m_ = 1.15 min (ACN/H_2_O 50:50); λ_max_ (nm): 275; gradient HPLC: 96.8% at 254 nm, t_ms_ = 12.2 min, t_m_ = 1.25 min.

##### 3-(7-Ethyl-1H-indol-3-yl)-4-(5-methoxy-1H-indol-3-yl)cyclobut-3-ene-1,2-dione (**2al**)

Synthesis according to General Procedure D from 3-chloro-4-(5-methoxy-1*H*-indol-3-yl)cyclobut-3-ene-1,2-dione (**5c**, 267 mg, 1.02 mmol) and 7-ethyl-1*H*-indole (0.205 mL, 1.50 mmol). Crystallization from acetone yielded a yellow powder (62 mg, 16%). IR (KBr): 3400 cm^−1^, 3379 cm^−1^ (NH), 1753 cm^−1^, 1714 cm^−1^ (C=O), 1612 cm^−1^, 1587 cm^−1^,1536 cm^−1^, 1514 cm^−1^ (C=C, arom.); ^1^H-NMR (400 MHz, DMSO-*d*_6_): δ (ppm) = 1.29 (t, *J* = 7.5 Hz, 3H, CH_2_-CH_3_), 2.94 (q, *J* = 7.5 Hz, 2H, CH_2_-CH_3_), 3.51 (s, 3H, CH_3_), 6.89 (dd, *J* = 8.8, 2.5 Hz, 1H, ArH), 7.03–7.14 (m, 2H, ArH), 7.29 (d, *J* = 2.5 Hz, 1H, ArH), 7.47 (d, *J* = 8.8 Hz, 1H, ArH), 7.70 (dd, *J* = 7.2, 1.8 Hz, 1H, ArH), 8.12 (d, *J* = 3.2 Hz, 1H, ArH), 8.23 (d, *J* = 3.1 Hz, 1H, ArH), 12.34 (d, *J* = 3.2 Hz, 1H, NH), 12.44 (d, *J* = 3.1 Hz, 1H, NH); ^13^C-NMR (101 MHz, DMSO-*d*_6_): δ (ppm) = 14.6 (CH_2_-CH_3_), 54.8 (OCH_3_), 23.6 (CH_2_-CH_3_), 104.8, 112.9, 113.4, 120.1, 121.4, 121.8, 130.5, 131.5 (CH), 106.3, 106.8, 125.2, 125.6, 128.2, 131.7, 135.4, 154.6, 176.4, 177.2, 194.5, 195.1 (C); C_23_H_18_N_2_O_3_ [370.41]; MS (EI): *m*/*z* (%) = 370 [M]^+^ (30), 314 [M^+^ − 56] (100); HRMS (EI) (*m*/*z*): Calcd. for [M]^+^ = 370.13119, found [M]^+^ = 370.13142; isocratic HPLC: 95.5% at 254 nm, 96.7% at 280 nm, t_ms_ = 5.71 min, t_m_ = 1.15 min (ACN/H_2_O 50:50); λ_max_ (nm): 350, 273; gradient HPLC: 96.2% at 254 nm, t_ms_ = 11.9 min, t_m_ = 1.25 min.

##### 3-(6-Bromo-1H-indol-3-yl)-4-(5-methoxy-1H-indol-3-yl)cyclobut-3-ene-1,2-dione (**2am**)

Synthesis according to General Procedure D from 3-chloro-4-(5-methoxy-1*H*-indol-3-yl)cyclobut-3-ene-1,2-dione (**5c**, 267 mg, 1.02 mmol) and 6-bromo-1*H*-indole (293 mg, 1.49 mmol). Crystallization from ethanol/toluene yielded a yellow powder (42 mg, 10%). IR (KBr): 3307 cm^−1^ (NH), 1751 cm^−1^, 1710 cm^−1^ (C=O), 1611 cm^−1^, 1529 cm^−1^,1510 cm^−1^ (C=C, arom.); ^1^H-NMR (600 MHz, DMSO-*d*_6_): δ (ppm) = 3.56 (s, 3H, O-CH_3_), 6.91 (dd, *J* = 8.8, 2.5 Hz, 1H, ArH), 7.27–7.34 (m, 2H, ArH), 7.48 (d, *J* = 8.8 Hz, 1H, ArH), 7.77 (d, *J* = 1.7 Hz, 1H, ArH), 7.89 (d, *J* = 8.5 Hz, 1H, ArH), 8.20 (s, 1H, ArH), 8.28 (s, 1H, ArH), 12.43 (s, 2H, NH); ^13^C-NMR (151 MHz, DMSO-*d*_6_): δ (ppm) = 54.9 (CH_3_), 104.8, 113.0, 113.52, 115.2, 124.0, 124.4, 131.8 (2C) (CH), 106.2, 106.5, 115.7, 124.2, 125.6, 131.7, 137.7, 154.8, 175.7, 177.5, 194.3, 195.2 (C); C_21_H_13_BrN_2_O_3_ [421.25]; Anal. calcd. for C_21_H_13_BrN_2_O_3_: C 59.88, H 3.11, N 6.65; found C 59.59, H 3.04, N 6.59; MS (EI): *m*/*z* (%) = 420 [M]^+^ (27), 364 [M^+^ − 56] (100); isocratic HPLC: 97.5% at 254 nm, 98.2% at 280 nm, t_ms_ = 4.25 min, t_m_ = 1.15 min (ACN/H_2_O 50:50); λ_max_ (nm): 278; gradient HPLC: 95.0% at 254 nm, t_ms_ = 12.0 min, t_m_ = 1.25 min.

##### 3-(4-Bromo-1H-indol-3-yl)-4-(5-methoxy-1H-indol-3-yl)cyclobut-3-en-1,2-dione **(2an**)

Synthesis according to General Procedure D from 3-chloro-4-(5-methoxy-1*H*-indol-3-yl)cyclobut-3-ene-1,2-dione (**5c**, 267 mg, 1.02 mmol) and 4-bromo-1*H*-indole (0.188 mL, 1.50 mmol). Crystallization from ethyl acetate yielded a yellow powder (20 mg, 5%). IR (KBr): 3238 cm^−1^ (NH), 1769 cm^−1^, 1713 cm^−1^ (C=O), 1623 cm^−1^, 1528 cm^−1^,1517 cm^−1^ (C=C, arom.); ^1^H-NMR (600 MHz, DMSO-*d*_6_): δ (ppm) = 2.96 (s, 3H, CH_3_), 6.76 (dd, *J* = 8.8, 2.5 Hz, 1H, ArH), 7.10–7.18 (m, 1H, ArH), 7.26–7.34 (m, 2H, ArH), 7.37–7.45 (m, 1H, ArH), 7.58 (dd, *J* = 8.2, 0.8 Hz, 1H, ArH), 7.91 (s, 1H, ArH), 8.40 (s, 1H, ArH), 12.37 (s, 2H, NH); ^13^C-NMR (151 MHz, DMSO-*d*_6_): δ (ppm) = 53.8 (CH_3_), 111.9, 113.2, 113.4, 113.6, 123.9, 124.6, 128.4, 132.7 (CH), 103.0, 105.3, 107.3, 125.1, 125.9, 131.7, 137.8, 154.9, 178.6, 183.6, 194.7, 198.0 (C); C_21_H_13_BrN_2_O_3_ [421.25] MS (EI): *m*/*z* (%) = 420 [M]^+^ (26), 364 [M^+^ − 56] (100); HRMS (EI) (*m*/*z*): Calcd. for [M]^+∙^= 420.01041, found [M]^+^ = 420.01102; isocratic HPLC: 97.2% at 254 nm, 97.8% at 280 nm, t_ms_ = 3.19 min, t_m_ = 1.15 min (ACN/H_2_O 45:55); λ_max_ (nm): 276, 364; gradient HPLC: 96.2% at 254 nm, t_ms_ = 10.9 min, t_m_ = 1.25 min.

##### 3-(5-Bromo-1H-indol-3-yl)-4-(5-methoxy-1-methyl-1H-indol-3-yl)cyclobut-3-en-1,2-dione (**2ao**)

Synthesis according to General Procedure D from 3-(5-bromo-1*H*-indol-3-yl)-4-chlorocyclobut-3-ene-1,2-dione (**5f**, 314 mg, 1.01 mmol) and 5-methoxy-1-methyl-1*H*-indole (193 mg, 1.20 mmol). Crystallization from chloroform/DMF yielded a yellow powder (80 mg, 18%). IR (KBr): 3422 cm^−1^ (NH), 1751 cm^−1^, 1712 cm^−1^ (C=O), 1625 cm^−1^, 1544 cm^−1^,1518 cm^−1^ (C=C, arom.); ^1^H-NMR (600 MHz, DMSO-*d*_6_): δ (ppm) = 3.58 (s, 3H, CH_3_), 3.97 (d, *J* = 0.4 Hz, 3H, CH_3_), 6.95–7.01 (m, 1H, ArH), 7.34 (d, *J* = 2.4 Hz, 1H, ArH), 7.37–7.43 (m, 1H, ArH), 7.56 (ddd, *J* = 11.3, 8.8, 0.5 Hz, 2H, ArH), 8.21–8.28 (m, 2H, ArH), 8.41 (t, *J* = 0.5 Hz, 1H, ArH), 12.61 (d, *J* = 2.9 Hz, 1H, NH); ^13^C-NMR (151 MHz, DMSO-*d*_6_): δ (ppm) = 33.6, 55.0 (CH_3_), 105.2, 112.1, 112.9, 114.5, 124.9, 125.6, 132.1, 135.0 (CH), 105.0, 106.1, 113.7, 126.0, 127.2, 132.6, 135.4, 155.1, 175.4, 176.5, 194.2, 194.9 (C); C_22_H_15_BrN_2_O_3_ [435.28]; Anal. calcd. for C_22_H_15_BrN_2_O_3_: C 60.71, H 3.47, N 6.44; found C 60.52, H 3.19, N 6.32; MS (EI): *m*/*z* (%) = 434 [M]^+^ (30), 378 [M^+^ − 56] (100); isocratic HPLC: 98.3% at 254 nm, 99.0% at 280 nm, t_ms_ = 4.09 min, t_m_ = 1.15 min (ACN/H_2_O 60:40); λ_max_ (nm): 229, 278, 350; gradient HPLC: 97.0% at 254 nm, t_ms_ = 12.7 min, t_m_ = 1.25 min.

##### 3-(5-Bromo-1H-indol-3-yl)-4-(5-hydroxy-1H-indol-3-yl)cyclobut-3-ene-1,2-dione (**2ap**)

To a suspension of 3-(5-bromo-1*H*-indol- 3-yl)-4-(5-methoxy-1*H*-indol-3-yl)cyclobut-3-ene-1,2-dione (**2ad**, 100 mg, 0.237 mmol) in anhydrous dichloromethane (10 mL) was added a solution of boron tribromide (1 M in dichloromethane, 4.00 mL, 4 mmol). The reaction was stirred at rt for 15 h. Under ice cooling, water (50 mL) was carefully added and the mixture was stirred for a further hour. The suspension was extracted with ethyl acetate (2 × 100 mL). The combined organic layers were washed with water (2 × 50 mL) and saturated NaCl solution (1 × 50 mL) and dried with anhydrous Na_2_SO_4_. After subsequent evaporation to dryness, the resulting residue was crystallized from petroleum ether/acetone to yield a yellow powder (55 mg, 57%). IR (KBr): 3422 cm^−1^ (NH), 1751 cm^−1^, 1712 cm^−1^ (C=O), 1625 cm^−1^, 1544 cm^−1^,1518 cm^−1^ (C=C, arom.); ^1^H-NMR (600 MHz, DMSO-*d*_6_): δ (ppm) = 6.78 (dd, *J* = 8.7, 2.4 Hz, 1H, ArH), 7.23 (d, *J* = 2.4 Hz, 1H, ArH), 7.39 (d, *J* = 8.8 Hz, 1H, ArH), 7.41 (dd, *J* = 8.6, 2.0 Hz, 1H, ArH), 7.55 (d, *J* = 8.6 Hz, 1H, ArH), 8.14 (d, *J* = 3.1 Hz, 1H, ArH), 8.24 (d, *J* = 3.2 Hz, 1H, ArH), 8.30 (d, *J* = 1.9 Hz, 1H, ArH), 9.14 (s, 1H, ArH), 12.30 (d, *J* = 3.1 Hz, 1H, NH), 12.55 (d, *J* = 3.1 Hz, 1H, NH); ^13^C-NMR (151 MHz, DMSO-*d*_6_): δ (ppm) = 107.3, 113.1, 113.3, 114.6, 124.9, 125.6, 131.4, 132.0 (CH), 105.7, 106.1, 113.7, 125.9, 127.2, 131.0, 135.5, 152.7, 175.4, 177.4, 194.4, 194.9 (C); C_20_H_11_BrN_2_O_3_ [407.2]; Anal. calcd. for C_20_H_11_BrN_2_O_3_: C 58.99, H 2.72, N 6.88; found C 58.61, H 2.74, N 6.35; MS (EI): *m*/*z* (%) = 406 [M]^+^ (29), 350 [M^+^ − 56] (100); isocratic HPLC: 96.4% at 254 nm, 98.5% at 280 nm, t_ms_ = 5.35 min, t_m_ = 1.15 min (ACN/H_2_O 40:60); λ_max_ (nm): 255, 309; gradient HPLC: 95.0% at 254 nm, t_ms_ = 10.4 min, t_m_ = 1.25 min.

##### 3,4-Bis(5-iodo-1H-indol-3-yl)cyclobut-3-ene-1,2-dione **(2aq**)

Synthesis according to General Procedure E from squaric acid dichloride (**4**, 150 mg, 0.99 mmol) anf 5-iodo-1*H*-indole (729 mg, 3.00 mmol). Crystallization from ethyl acetate/ethanol yielded an orange powder (70 mg, 12%). IR (KBr): 3375 cm^−1^ (NH), 1755 cm^−1^, 1695 cm^−1^ (C=O), 1529 cm^−1^, 1505 cm^−1^ (C=C, arom.); ^1^H-NMR (600 MHz, DMSO-*d_6_*): δ (ppm) = 7.42–7.47 (m, 2H, ArH), 7.57 (dd, *J* = 8.5, 1.7 Hz, 2H, ArH), 8.30 (s, 2H, ArH), 8.34 (d, *J* = 1.8 Hz, 2H, ArH), 12.62 (s, 2H, NH); ^13^C-NMR (151 MHz, DMSO-*d_6_*): δ (ppm) = 115.5, 131.6, 131.6, 132.2 (CH), 86.1, 106.0, 128.0, 136.4, 177.1, 195.1 (C); C_20_H_10_I_2_N_2_O_2_ [564.12]; Anal. calcd. for C_20_H_10_I_2_N_2_O_2_: C 42.58, H 1.79, N 4.97; found C 45.52, H 1.52, N 4.76; MS (EI): *m*/*z* (%) = 564 [M]^+∙^(33), 508 [M^+^ − 56] (100); isocratic HPLC: 97.1% at 254 nm and 98.0% at 280 nm, t_ms_ = 5.03 min, t_m_ = 1.11 min (ACN/H_2_O 60:40); λ_max_ (nm): 280, 329, 344; gradient HPLC: 96.5% at 254 nm, t_ms_ = 13.4 min, t_m_ = 1.25 min.

##### 3-[5-(Benzyloxy)-1H-indol-3-yl]-4-(5-methoxy-1H-indol-3-yl)cyclobut-3-ene-1,2-dione (**2ar**)

Synthesis according to General Procedure F from 3-chloro-4-(5-methoxy-1*H*-indol-3-yl)cyclobut-3-ene-1,2-dione (**5c**, 274 mg, 1.05 mmol) and 5-(Benzyloxy)-1*H*-indole (670 mg, 3.00 mmol). Crystallization from petroleum ether/ethylacetate yielded an orange powder (35 mg, 7%). IR (KBr): 3307 cm^−1^ (NH), 1747 cm^−1^, 1711 cm^−1^ (C=O), 1624 cm^−1^, 1536 cm^−1^, 1508 cm^−1^ (C=C, arom.); ^1^H-NMR (600 MHz, DMSO-*d_6_*): δ (ppm) = 3.47 (s, 3H, O-CH_3_), 4.71 (s, 2H, O-CH_2_), 6.92 (ddd, *J* = 27.7, 8.8, 2.5 Hz, 2H, ArH), 7.23–7.36 (m, 7H, ArH), 7.47 (dd, *J* = 11.3, 8.8 Hz, 2H, ArH), 8.22 (s, 2H, ArH), 12.33 (s, 2H, NH); ^13^C-NMR (101 MHz, DMSO-*d_6_*): δ (ppm) = 54.8 (CH_3_), 69.4 (CH_2_), 104.7, 106.2 (2C), 113.1, 113.4 (2C), 113.5, 127.5 (2C), 127.7, 128.4 (2C), 131.4, 131.7 (CH), 106.4 (2C) 125.7, 125.8, 131.8, 137.1, 153.6, 154.6, 176.3, 176.4, 194.7, 194.8 (C); C_28_H_20_N_2_O_4_ [448.48]; MS (EI): *m*/*z* (%) = 448 [M]^+^ (74), 392 [M^+^ − 56] (100); HRMS (EI) (*m*/*z*): Calcd. for [M]^+^∙= 448.14176, found [M]^+^ = 448.14204; isocratic HPLC: 98.4% at 254 nm and 99.1% at 280 nm, t_ms_ = 7.02 min, t_m_ = 1.11 min (ACN/H_2_O 50:50); λ_max_ (nm): 352, 277; gradient HPLC: 98.5% at 254 nm, t_ms_ = 12.2 min, t_m_ = 1.25 min.

### 4.3. Molecular Docking

For docking studies, the plasmodial protein kinase crystal structures 1V0P, 4MVF, 2PML, 3LLT, and 3NIE were downloaded from the Protein Data Bank [32] and three homology models of *Pf*GSK-3 were selected, which had been created using the crystal structures of human GSK-3 (pdb-id: 1J1B, 2OW3 and 1R0E, respectively) [33]. After protonation optimization and energy minimization (defaults) conducted with MOE (Molecular Operating Environment, 2013.08 ed., Chemical Computing Group Inc. Montreal, QB, Canada), the .pdb files of the protein structures were used for the subsequent docking experiments. In the same way, the ligands were prepared with MOE and saved as mol2 files. The docking experiments were carried out with GOLD Suite 2.2.5 [31] without the definition of constraints. The spatial extent of the ATP binding pocket was defined as a 10 Å zone with respect to either the originally enclosed ligand or, in the homology models, with respect to the gatekeeper residue. After the relevant water molecules in the binding pocket for the automatic toggle were established, the remaining water molecules were removed. For the respective ligands, ten genetic algorithm runs were generated, the options “save lone pairs” and “allow early termination” being disabled. The docking accuracy was set to 200%. The evaluated docking solutions by the integrated ChemScore.kinase templates were stored with ChemScore values in descending order as .mol2 or .sdf files. The thus rated poses were mapped and analyzed using the visualization program Chimera [51]. By additional visual inspection, the poses were interpreted regarding meaningful orientation, considering important polar interactions, occupation of the hydrophobic back pocket, and optional formation of halogen bonds. Chimera is developed by the Resource for Biocomputing, Visualization, and Informatics at the UCSF (supported by NIFMS P41-GM103311).

### 4.4. Luciferase-Based Viability Screening for Antiplasmodial Activity

Screening for the antiplasmodial activity of test compounds was performed by luciferase-based viability assays as reported previously [30]. In brief, asexual erythrocytic stages of transgenic NF54-*luc P. falciparum* were cultured as described elsewhere [28,52]. Parasite cultures with parasitaemia of 0.5–1% were incubated in the presence of 3 µM test compounds for 48 h. After the subsequent addition of Bright-Glo substrate solution (Promega, Madison, WI), the resulting luminescence was measured. Untreated cultures (0.01% DMSO) were used as negative controls. Experiments were performed in triplicate and were repeated as a whole at least twice. Blasticidin (Sigma-Aldrich, St. Louis, MO, USA) was included as a positive control [53]. Test compounds exhibiting satisfactorily inhibition activity in 3 µM concentration (in most cases >20% inhibition of viability) were rated as actives of which IC_50_ values were determined from dose-response curves. 

### 4.5. Cytotoxicity Assay on Human THP-1 Cell Line

The cytotoxicity of selected antiplasmodial bisindolylcyclobutenediones was determined against THP-1 cells following a protocol described previously [54,55,56]. In brief, THP-1 cells were incubated with test compounds in serial compound dilutions in the presence of 0.01% DMSO at 37 °C. After 48 h, Alamar Blue was added, and after 4 h the fluorescence was read (λ_ex_ = 544 nm, λ_em_ = 590 nm) using a microplate reader. THP-1 cells in wells containing medium plus 0.01% DMSO served as negative controls. Experiments were performed in triplicate and were repeated as a whole at least twice.

### 4.6. Production of Recombinant PfGSK-3

*Pf*GSK-3 was produced as published before [42]. In brief, *Pf*GSK-3 was cloned into pOPIN J expression vector [57] which was transformed into *E. coli* BL21 (DE3)-RIL. Expression of recombinant 6xHis-GST-*Pf*GSK-3 was induced by incubation with isopropyl β-d-1-thiogalactopyranoside (IPTG). The recombinant protein was purified by affinity chromatography using glutathione resin and elution by digestion with human rhinovirus 3C protease.

### 4.7. PfGSK-3 Inhibition Assay

The *Pf*GSK-3 inhibition assay was carried out using the commercial KinaseGlo Plus assay (KinaseGlo Plus, Promega) as described before [42]. In brief, the kinase and a corresponding substrate (GS-1 substrate, Promega) in the presence of ATP were used to perform substrate phosphorylation. Subsequently, luminescence is generated utilizing luciferase which is a component of the KinaseGlo Reagent. The intensity of the luminescence signal corresponds to the amount of remaining ATP in the reaction mixture. For the investigation, potential kinase inhibitors were dissolved in DMSO and were added directly to the kinase reaction. The final DMSO concentration in the kinase reaction was at most 1%. As the negative control, 1% DMSO without test compound was measured. A control reaction without kinase was measured simultaneously. The difference between kinase reaction and control reaction corresponds to the kinase activity. While the published selective *Pf*GSK-3 inhibitor 5v [28] exhibited the expected inhibitory activity in this assay, the potent antiplasmodial bisindolylcyclobutenediones **2k**, **2ad**, and **2ae** failed to show kinase inhibitory activity up to a concentration of 10 µM.

## 5. Conclusions

In view of the high incidence and the spreading resistance to approved drugs, new active agents against the malaria pathogens are urgently needed. Such new drugs should hit previously unaddressed targets and thus be based on novel mechanisms of action. Although plasmodial protein kinases have been frequently proposed as biological targets for malaria therapy, no drugs have yet been developed on this basis. Against this background, novel *N*-unsubstituted bisindolylcyclobutenediones were synthesized as analogs to the kinase-inhibiting bisindolylmaleimides using a sequential Friedel-Crafts acylation starting from squaric acid dichloride. Docking experiments on various plasmodial kinases revealed plausible orientations in the ATP-binding pocket of the enzymes. When the new compounds were screened in vitro on erythrocytic *P. falciparum* forms, several derivatives showed good inhibition values with favorable selectivity profiles. The 7-chloro-5’-methoxy-substituted compound **2ai** proved to be the most interesting representative, showing submicromolar antiparasitic activity and a three-digit selectivity index against a human cell line. Whether inhibition of plasmodial protein kinases is responsible for the antiparasitic activity of the new compounds remains unclear for the time being. The protein kinase *Pf*GSK-3, initially assumed to be the target structure, was not inhibited by particularly active representatives of the new substance class. As selective submicromolar antiplasmodial agents, the *N*-unsubstituted bisindolylcyclobutenediones are promising starting structures in searching for therapeutic antimalarials, although for a rational development the biological target addressed by these compounds remains to be identified.

## 6. Patents

The syntheses and antiplasmodial activities of compounds reported in this article have been claimed by some of the authors in a patent application [58].

## Figures and Tables

**Table 1:** Structures and antiplasmodial activity of bisindolylcyclobutenediones **2a**–**ar** and bisindolylmaleimide **8** ^a^.

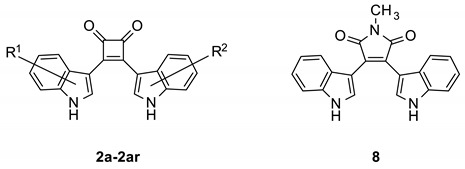				
Entry	R^1^	R^2^	IC_50_ [µM] ^b^	Inhibition at 3 µM [%] ^c^
**2a**	H	H	8.33	17.9 ± 3.3
**2b**	H	5-Br	3.02	65.9 ± 1.8
**2c**	H	5-Cl	4.23	44.7 ± 0.5
**2d**	H	5-OCH_3_	n.d.	18.3 ± 5.0
**2e**	H	5-OCH_2_Ph	n.d.	−4.43 ± 10.6
**2f**	H	5-CN	6.72	26.8 ± 1.56
**2g**	H	2-CH_3_	>30	23.4 ± 5.4
**2h**	H	2-Ph	6.38	65.0 ± 2.8
**2i**	1-CH_3_	1-CH_3_	n.d.	3.53 ± 1.7
**2k**	H	1-[3-(dimethyl-amino)propyl]	0.376	98.1 ± 1.8
**2l**	2-CH_3_	2-CH_3_	n.d.	18.3 ± 6.3
**2m**	2-Ph	2-Ph	2.47	41.1 ± 0.6
**2n**	5-OCH_3_	5-OCH_3_	n.d.	19.7 ± 3.6
**2o**	5-Br	5-Br	>30	n.d.
**2p**	5-CN	5-CN	n.d.	−3.10 ± 0.9
**2q**	H	1-CH_3_	2.97	52.0 ± 1.8
**2r**	H	5-I	0.915	31.3 ± 2.7
**2s**	2-Ph	5-Br	n.d.	16.0 ± 0.7
**2t**	5-Br	1-CH_3_	n.d.	1.38 ± 3.3
**2u**	2-Ph	1-CH_3_	1.72	67.7 ± 0.7
**2v**	5-OCH_3_	2-CH_3_	10.0	34.2 ± 8.0
**2w**	2-Ph	2-CH_3_	9.26	41.1 ± 1.7
**2x**	5-Br	2-CH_3_	>30	45.6 ± 1.8
**2y**	1-CH_3_	2-CH_3_	2.67	59.6 ± 0.2
**2z**	5-Br, 1-CH_3_	2-CH_3_	n.d.	12.7 ± 2.8
**2aa**	2-Ph	5-OCH_3_	n.d.	22.4 ± 2.7
**2ab**	5-F	5-OCH_3_	n.d.	19.2 ± 3.1
**2ac**	5-Cl	5-OCH_3_	1.52	87.0 ± 0.5
**2ad**	5-Br	5-OCH_3_	0.47	98.8 ± 0.7
**2ae**	5-I	5-OCH_3_	0.64	99.1 ± 0.3
**2af**	1-CH_3_	5-OCH_3_	n.d.	13.6 ± 3.9 ^d^
**2ag**	5-Br, 1-CH_3_	5-OCH_3_	n.d.	−0.06 ± 2.43
**2ah**	5-CN	5-OCH_3_	6.92	26.8 ± 1.1
**2ai**	7-Cl	5-OCH_3_	0.691	84.5 ± 1.0
**2aj**	7-Br	5-OCH_3_	0.296	99.5 ± 0.4
**2ak**	7-I	5-OCH_3_	0.116	98.8 ± 1.2
**2al**	7-C_2_H_5_	5-OCH_3_	0.511	87.0 ± 0.5
**2am**	6-Br	5-OCH_3_	n.d.	9.55 ± 5.8
**2an**	4-Br	5-OCH_3_	n.d.	25.7 ± 2.7
**2ao**	5-Br	1-CH_3_, 5-OCH_3_	0.504	95.4 ± 1.1
**2ap**	5-Br	5-OH	n.d.	2.52 ± 1.4
**2aq**	5-I	5-I	n.d.	1.79 ± 1.8
**2ar**	5-OBz	5-OCH_3_	n.d.	21.7 ± 2.5
**8**	-	-	4.0	34.6 ± 0.8
BSD ^e^	-	-	0.288	97.6 ± 0.5

^a^ n.d. = not determined. ^b^ Concentration [µM] for 50% inhibition of *P. falciparum* (NF54-*luc* strain) erythrocytic stages. ^c^ Inhibition of *P. falciparum* (NF54-*luc* strain) erythrocytic stages at 3 µM. ^d^ determined at 30 µM. ^e^ Blasticidin (BSD) was used as the positive control.

**Table 2:** Selectivity of the most active bisindolylcyclobutenediones on *P. falciparum* parasites versus the human THP-1 cell line.

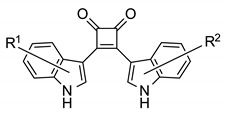					
Entry	R^1^	R^2^	IC_50, *Pf*_ [µM] ^a^	IC_50, THP-1_ [µM] ^b^	SI ^c^
**2b**	H	5-Br	3.02	23.0	7.6
**2h**	H	2-Ph	6.38	6.37	1.0
**2k**	H	1-[3-(dimethyl-amino)propyl]	0.38	12.8	34
**2q**	H	1-CH_3_	2.97	14.0	4.7
**2r**	H	5-I	0.92	7.25	7.9
**2u**	2-Ph	1-CH_3_	1.72	2.10	1.2
**2y**	1-CH_3_	2-CH_3_	2.67	8.40	3.1
**2ad**	5-Br	5-OCH_3_	0.47	14.1	30
**2ae**	5-I	5-OCH_3_	0.64	14.5	23
**2ai**	7-Cl	5-OCH_3_	0.69	179	259
**2aj**	7-Br	5-OCH_3_	0.30	1.42	4.7
**2ak**	7-I	5-OCH_3_	0.12	0.84	7.0
**2al**	7-C_2_H_5_	5-OCH_3_	0.51	0.70	1.4
**2ao**	5-Br	1-CH_3_, 5-OCH_3_	0.50	3.13	6.3

^a^ Inhibition (IC_50_) of *Plasmodium falciparum* (NF54-*luc* strain) erythrocytic stages. ^b^ Inhibition (IC_50_) of human THP-1 cell line. ^c^ Selectivity Index (SI) = IC_50, THP-1_/IC_50, *Pf.*_

## Data Availability

The data presented in this study are available in Section 4: “Materials and Methods” and in the Supplementary Materials published with this article.

## Supplementary Materials

The following are available online. Table S1: Structures of all test compounds, Table S2: ^1^H NMR-spectra of test compounds displayed in Table 2, Figure S1: Docking pose of **2b** (orange) and **2ad** (blue) in the ATP binding pocket of a *Pf*GSK-3 homology model.

## Sample Availability

Samples of the compounds are not available from the authors.
