# Supplementary material for: Synthesis and Antiplasmodial Activity of Bisindolylcyclobutenediones
Source: Molecules. 2021 Aug 5;26(16):4739. doi: 10.3390/molecules26164739 (PMC8402075; doi:10.3390/molecules26164739)
Supplement: Supplementary file 1 [file molecules-26-04739-s001.zip › molecules-1274475-supplementary.pdf]

D. H. Lande , A. Nasereddin, A. Alder, T. W. Gilberger, R. Dzikowski, J. Grünefeld, C. Kunick

# Synthesis and Antiplasmodial Activity of Bisindolylcyclobutenediones: Supplementary Materials

Content: Table S1: Structures of all test compounds 2 (pages 1-5), Table S2: $^{1}$H NMR-spectra of test compounds displayed in Table 2 (Pages 6-12), Figure S1: Docking pose of **2b** and **2ad** in a *Pf*GSK-3 homology model (page 13).

**Table S1:** Structures of compounds mentioned in the article

*3-Chloro-4-(1H-indol-3-yl)cyclobut-3-ene-1,2-dione* **(5a)**

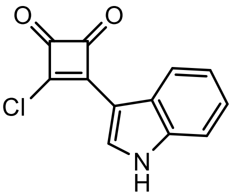

*3-Chloro-4-(1-methyl-1H-indol-3-yl)cyclobut-3-ene-1,2-dione* **(5b)**

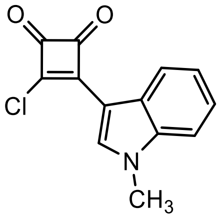

*3-Chloro-4-(5-methoxy-1H-indol-3-yl)cyclobut-3-ene-1,2-dione* **(5c)**

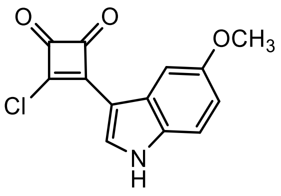

*3-Chloro-4-(2-phenyl-1H-indol-3-yl)cyclobut-3-ene-1,2-dione* **(5d)**

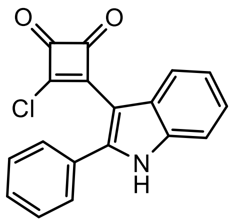

*3-(5-Bromo-1H-indol-3-yl)-4-chlorcyclobut-3-ene-1,2-dione* **(5e)**

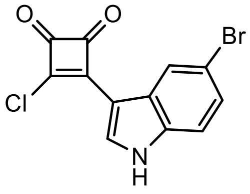

*3-Chloro-4-(2-methyl-1H-indol-3-yl)cyclobut-3-ene-1,2-dione* **(5f)**

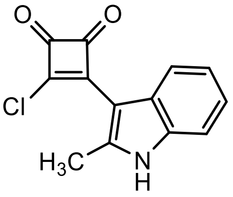

*3,4-Bis(1H-indol-3-yl)cyclobut-3-ene-1,2-dione* **(2a)**

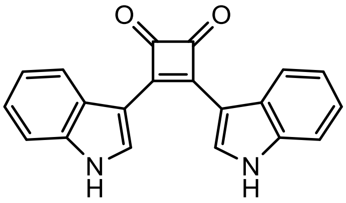

*3-(5-Bromo-1H-indol-3-yl)-4-(1H-indol-3-yl)cyclobut-3-ene-1,2-dione* **(2b)**

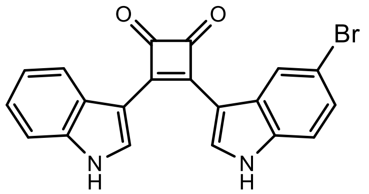

*3-(5-Chloro-1H-indol-3-yl)-4-(1H-indol-3-yl)cyclobut-3-ene-1,2-dione* **(2c).**

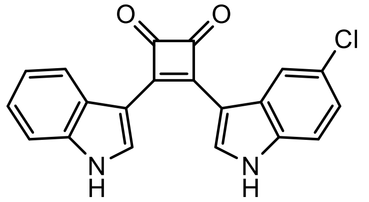

*3-(1H-Indol-3-yl)-4-(5-methoxy-1H-indol-3-yl)cyclobut-3-ene-1,2-dione* **(2d)**

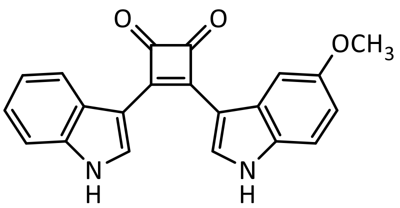

*3-[5-(Benzyloxy)-1H-indol-3-yl]-4-(1H-indol-3-yl)cyclobut-3-ene-1,2-dione* **(2e)**

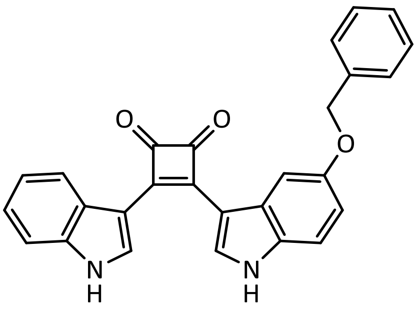

*3-[2-(1H-Indol-3-yl)-3,4-dioxocyclobut-1-ene-1-yl]-1H-indol-5-carbonitrile* **(2f)**

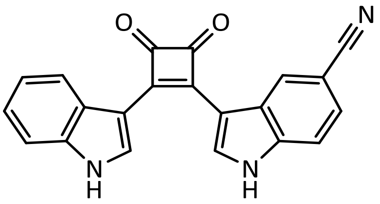

*3-(1H-Indol-3-yl)-4-(2-methyl-1H-indol-3-yl)cyclobut-3-ene-1,2-dione* **(2g)**

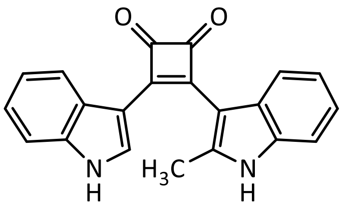

*3-(1H-Indol-3-yl)-4-(2-phenyl-1H-indol-3-yl)cyclobut-3-ene-1,2-dione* **(2h)**

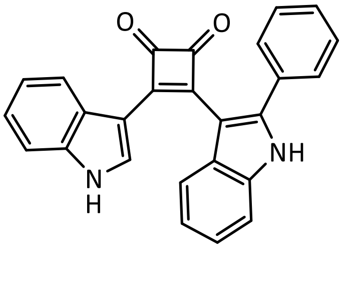

*3,4-Bis(1-methyl-1H-indol-3-yl)cyclobut-3-ene-1,2-dione* **(2i)**

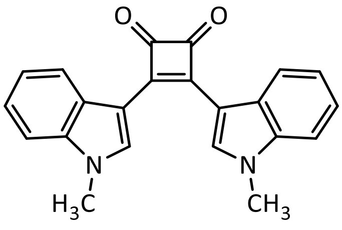

*3-{1-[3-(Dimethylamino)propyl]-1H-indol-3-yl}-4-(1H-indol-3-yl)cyclobut-3-ene-1,2-dione* **(2k)**

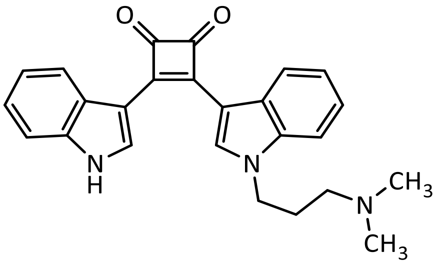

*3,4-Bis(2-methyl-1H-indol-3-yl)cyclobut-3-ene-1,2-dione* **(2l).**

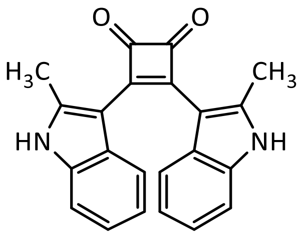

*3,4-Bis(2-phenyl-1H-indol-3-yl)cyclobut-3-ene-1,2-dione* **(2m)**

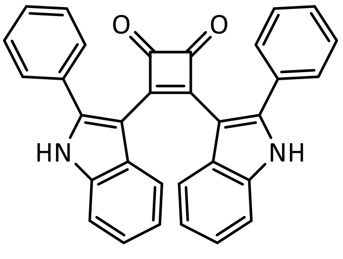

*3,4-Bis(5-methoxy-1H-indol-3-yl)cyclobut-3-ene-1,2-dione* **(2n)**

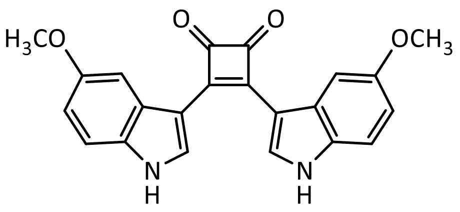

*3,4-Bis(5-bromo-1H-indol-3-yl)cyclobut-3-ene-1,2-dione* **(2o)**

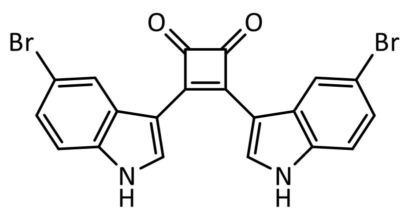

*3,3′-(3,4-Dioxocyclobut-1-ene-1,2-diyl)bis(1H-indole-5-carbonitrile)* **(2p)**

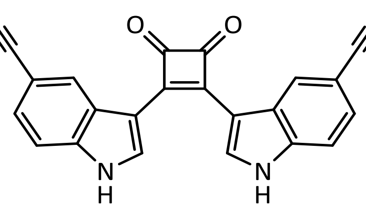

*3-(1H-Indol-3-yl)-4-(1-methyl-1H-indol-3-yl)cyclobut-3-ene-1,2-dione* **(2q)**

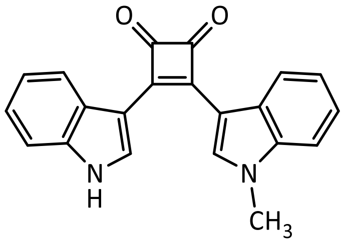

*3-(1H-Indol-3-yl)-4-(5-iod-1H-indol-3-yl)cyclobut-3-ene-1,2-dione* **(2r)**

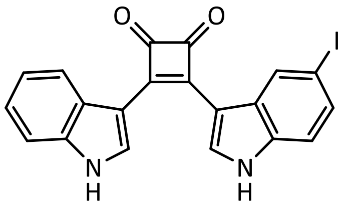

*3-(5-Bromo-1H-indol-3-yl)-4-(2-phenyl-1H-indol-3-yl)cyclobut-3-ene-1,2-dione* **(2s)**

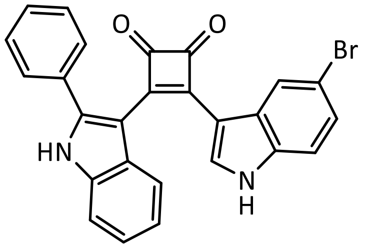

*3-(5-Bromo-1H-indol-3-yl)-4-(1-methyl-1H-indol-3-yl)cyclobut-3-ene-1,2-dione* **(2t)**

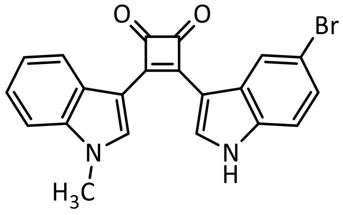

*3-(1-Methyl-1H-indol-3-yl)-4-(2-phenyl-1H-indol-3-yl)cyclobut-3-ene-1,2-dione* **(2u)**

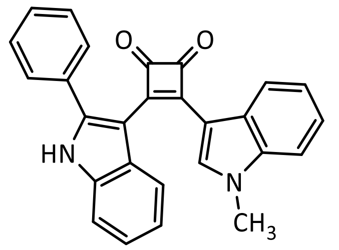

*3-(5-Methoxy-1H-indol-3-yl)-4-(2-methyl-1H-indol-3-yl)cyclobut-3-ene-1,2-dione* **(2v)**

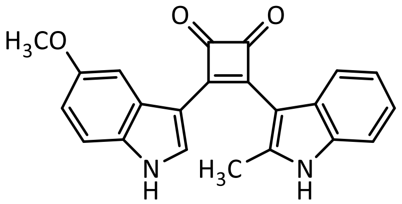

*3-(2-Methyl-1H-indol-3-yl)-4-(2-phenyl-1H-indol-3-yl)cyclobut-3-ene-1,2-dione* **(2w)**

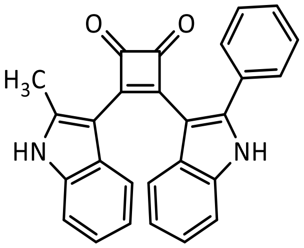

*3-(5-Bromo-1H-indol-3-yl)-4-(2-methyl-1H-indol-3-yl)cyclobut-3-ene-1,2-dione* **(2x)**

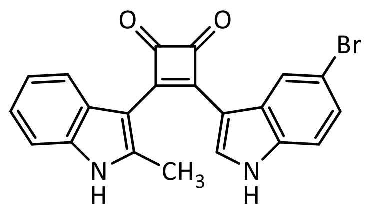

*3-(1-Methyl-1H-indol-3-yl)-4-(2-methyl-1H-indol-3-yl)cyclobut-3-ene-1,2-dione* **(2y)**

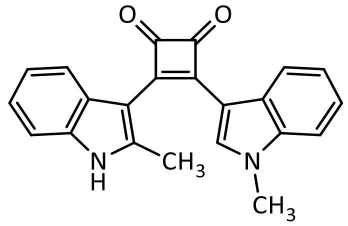

*3-(5-Bromo-1-methyl-1H-indol-3-yl)-4-(2-methyl-1H-indol-3-yl)cyclobut-3-ene-1,2-dione* **(2z)**

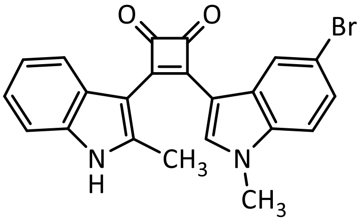

*3-(5-Methoxy-1H-indol-3-yl)-4-(2-phenyl-1H-indol-3-yl)cyclobut-3-ene-1,2-dione* **(2aa)**

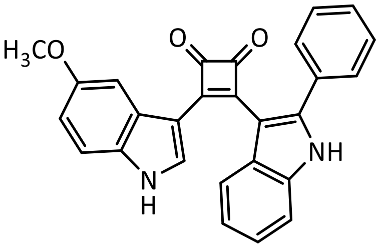

*3-(5-Fluoro-1H-indol-3-yl)-4-(5-methoxy-1H-indol-3-yl)cyclobut-3-ene-1,2-dione* **(2ab)**

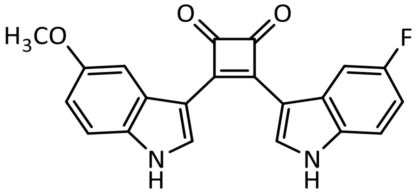

*3-(5-Chloro-1H-indol-3-yl)-4-(5-methoxy-1H-indol-3-yl)cyclobut-3-ene-1,2-dione* **(2ac)**

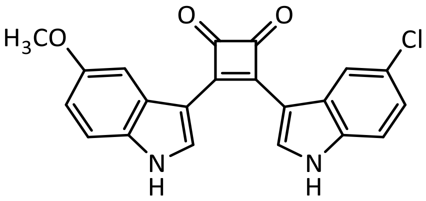

*3-(5-Bromo-1H-indol-3-yl)-4-(5-methoxy-1H-indol-3-yl)cyclobut-3-ene-1,2-dione* **(2ad)**

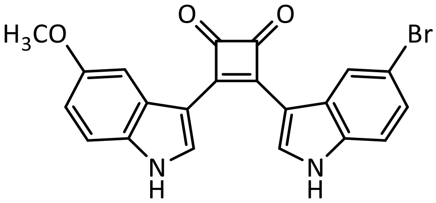

*3-(5-Iodo-1H-indol-3-yl)-4-(5-methoxy-1H-indol-3-yl)cyclobut-3-ene-1,2-dione* **(2ae)**

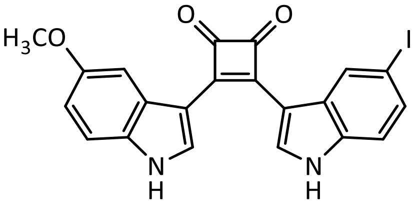

*3-(5-Methoxy-1H-indol-3-yl)-4-(1-methyl-1H-indol-3-yl)cyclobut-3-ene-1,2-dione* **(2af)**

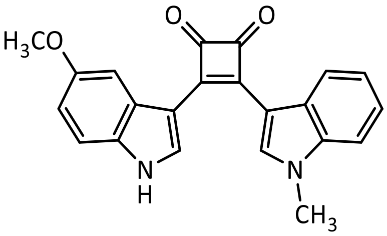

*3-(5-Bromo-1-methyl-1H-indol-3-yl)-4-(5-methoxy-1H-indol-3-yl)cyclobut-3-ene-1,2-dione* **(2ag)**

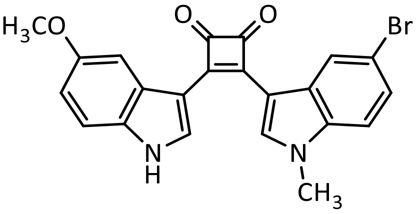

*3-[2-(5-Methoxy-1H-indol-3-yl)-3,4-dioxocyclobut-1-en-1-yl]-1H-indole-5-carbonitrile* **(2ah)**

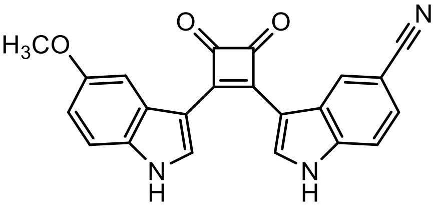

*3-(7-Chloro-1H-indol-3-yl)-4-(5-methoxy-1H-indol-3-yl)cyclobut-3-ene-1,2-dione* **(2ai)**

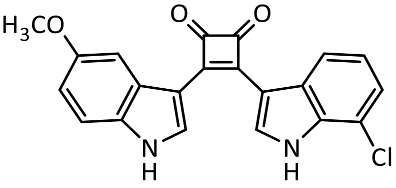

*3-(7-Bromo-1H-indol-3-yl)-4-(5-methoxy-1H-indol-3-yl)cyclobut-3-ene-1,2-dione* **(2aj)**

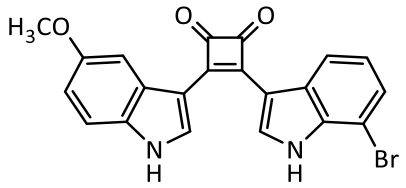

*3-(7-Iodo-1H-indol-3-yl)-4-(5-methoxy-1H-indol-3-yl)cyclobut-3-ene-1,2-dione* **(2ak)**

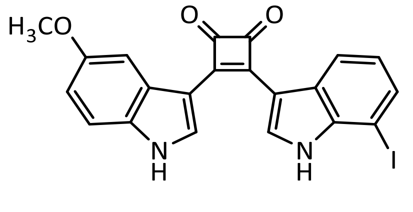

*3-(7-Ethyl-1H-indol-3-yl)-4-(5-methoxy-1H-indol-3-yl)cyclobut-3-ene-1,2-dione* **(2al)**

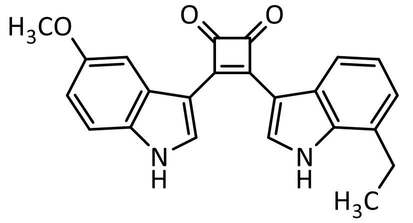

*3-(6-Bromo-1H-indol-3-yl)-4-(5-methoxy-1H-indol-3-yl)cyclobut-3-ene-1,2-dione* **(2am)**

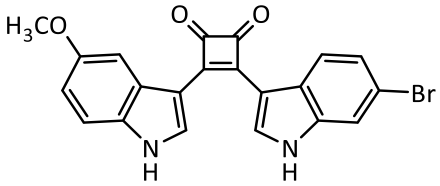

*3-(4-Bromo-1H-indol-3-yl)-4-(5-methoxy-1H-indol-3-yl)cyclobut-3-ene-1,2-dione* **(2an)**

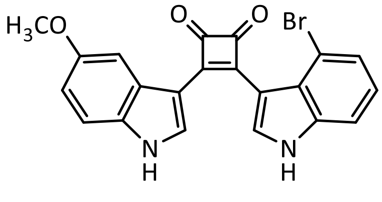

*3-(5-Bromo-1H-indol-3-yl)-4-(5-methoxy-1-methyl-1H-indol-3-yl)cyclobut-3-ene-1,2-dione* **(2ao)**

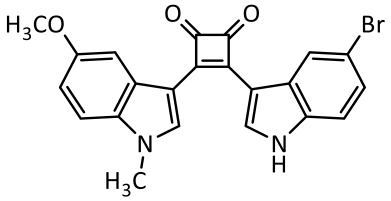

*3-(5-Bromo-1H-indol-3-yl)-4-(5-hydroxy-1H-indol-3-yl)cyclobut-3-ene-1,2-dione* **(2ap)**

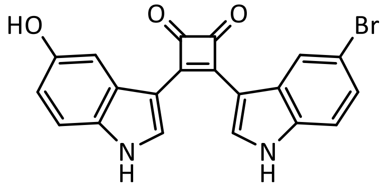

*3,4-Bis(5-iodo-1H-indol-3-yl)cyclobut-3-ene-1,2-dione* **(2aq)**

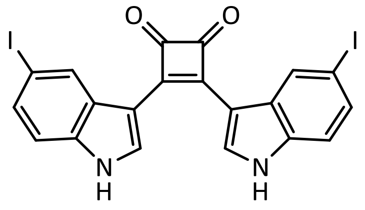

*3-[5-(Benzyloxy)-1H-indol-3-yl]-4-(5-methoxy-1H-indol-3-yl)cyclobut-3-ene-1,2-dione* **(2ar)**

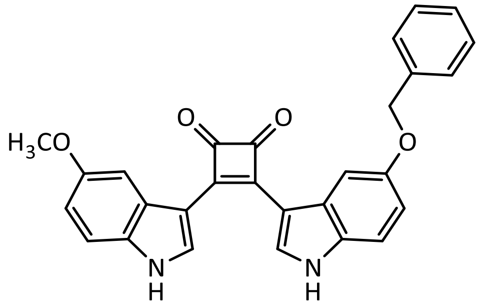

**Table S2:** $^{1}$H NMR Spectra of compounds displayed in Table 2

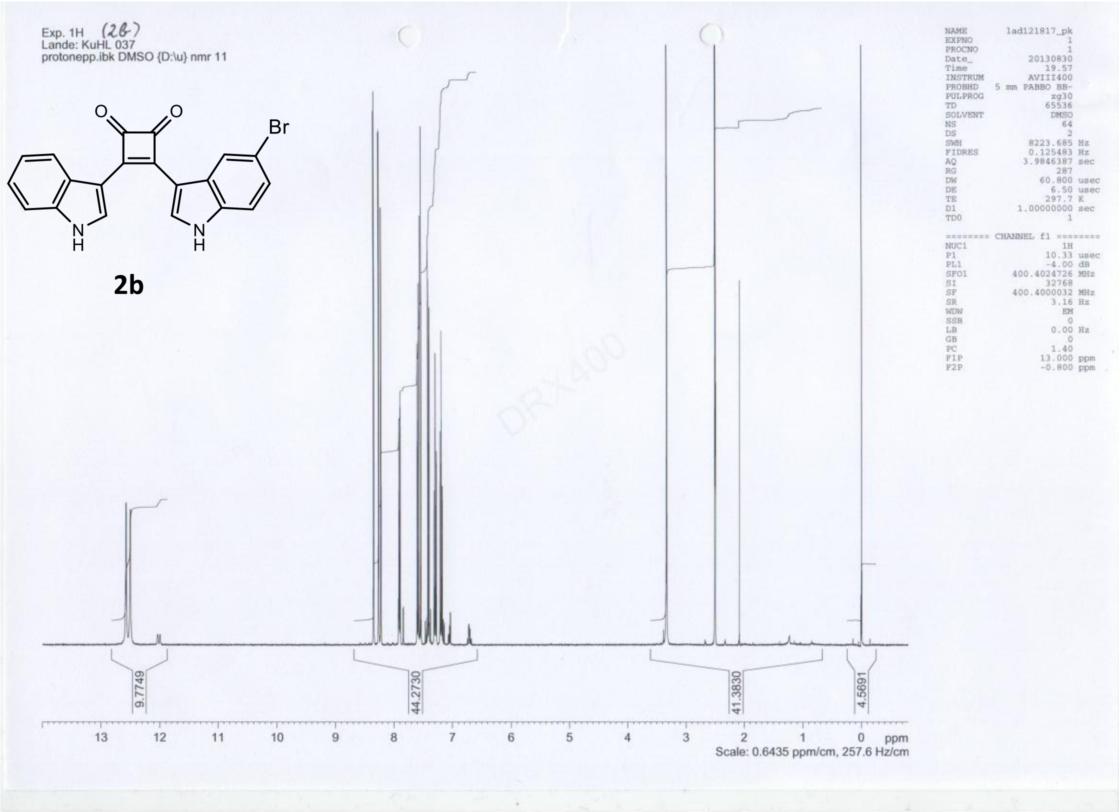


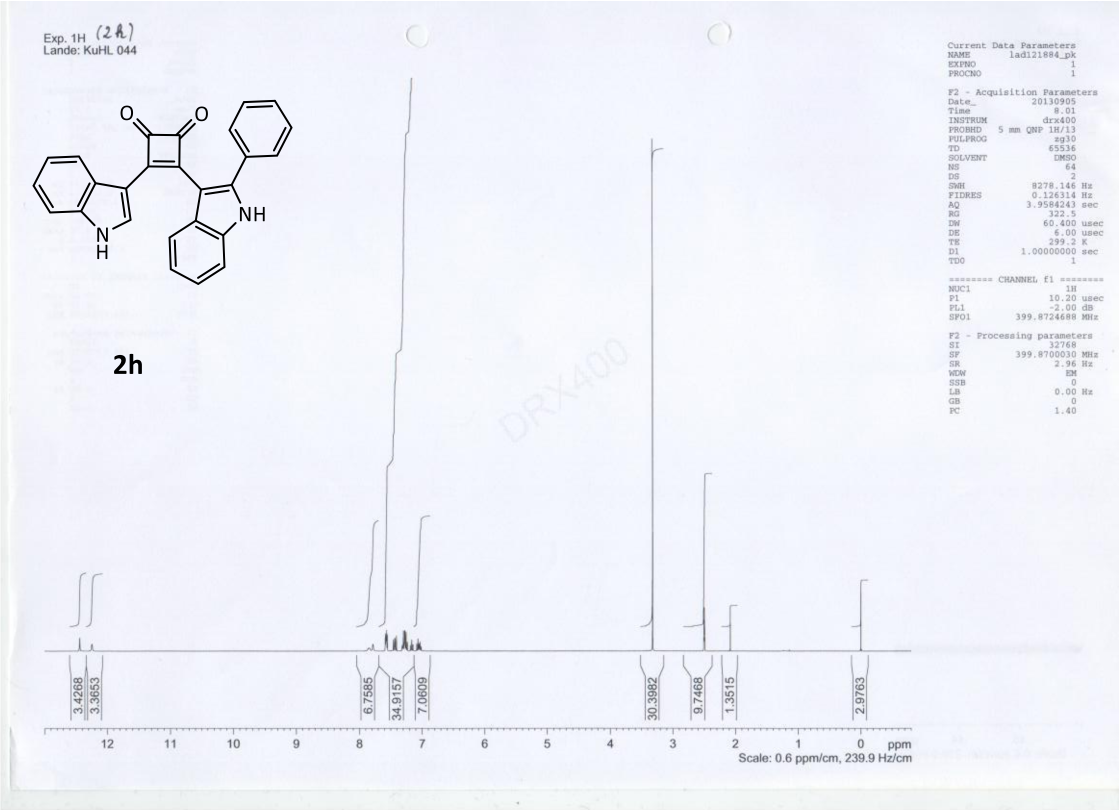

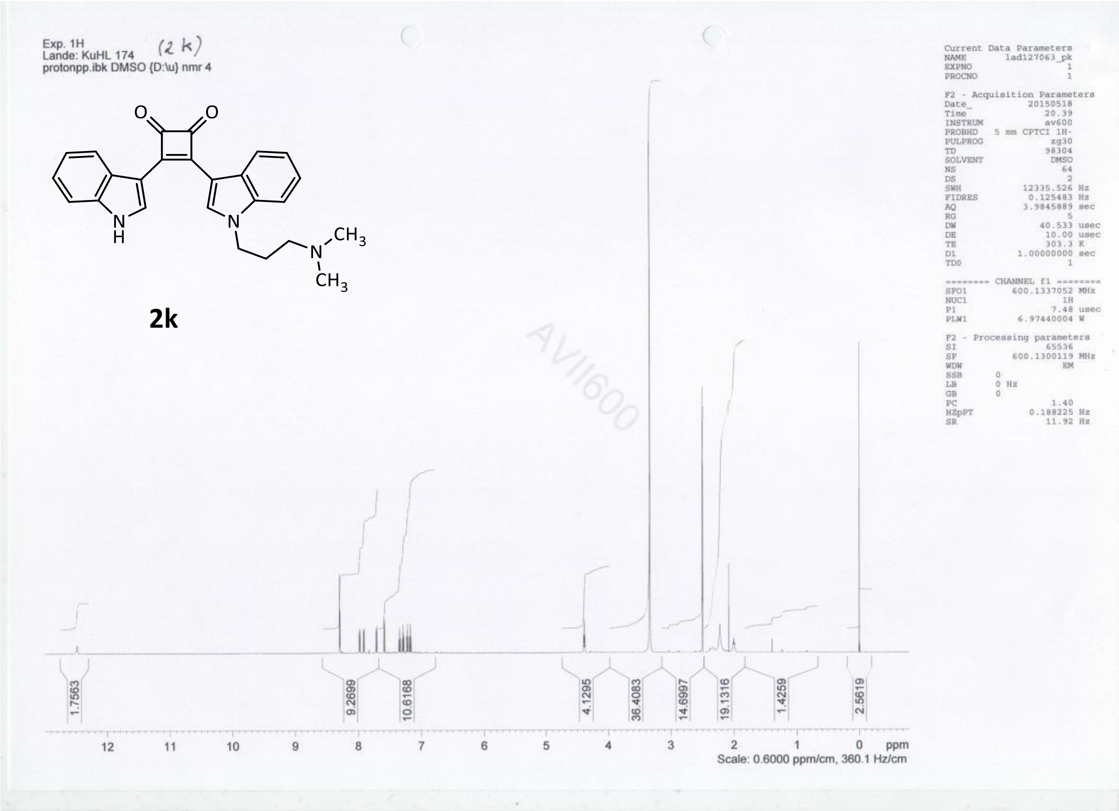
Exp. 1H
Lande: KuHL 174
protonpp.ibk DMSO {D:\u} nmr 4
2k
1.7563
9.2699
10.6168
4.1295
36.4083
14.6997
19.1316
1.4259
2.5619
ppm
Scale: 0.6000 ppm/cm, 360.1 Hz/cm

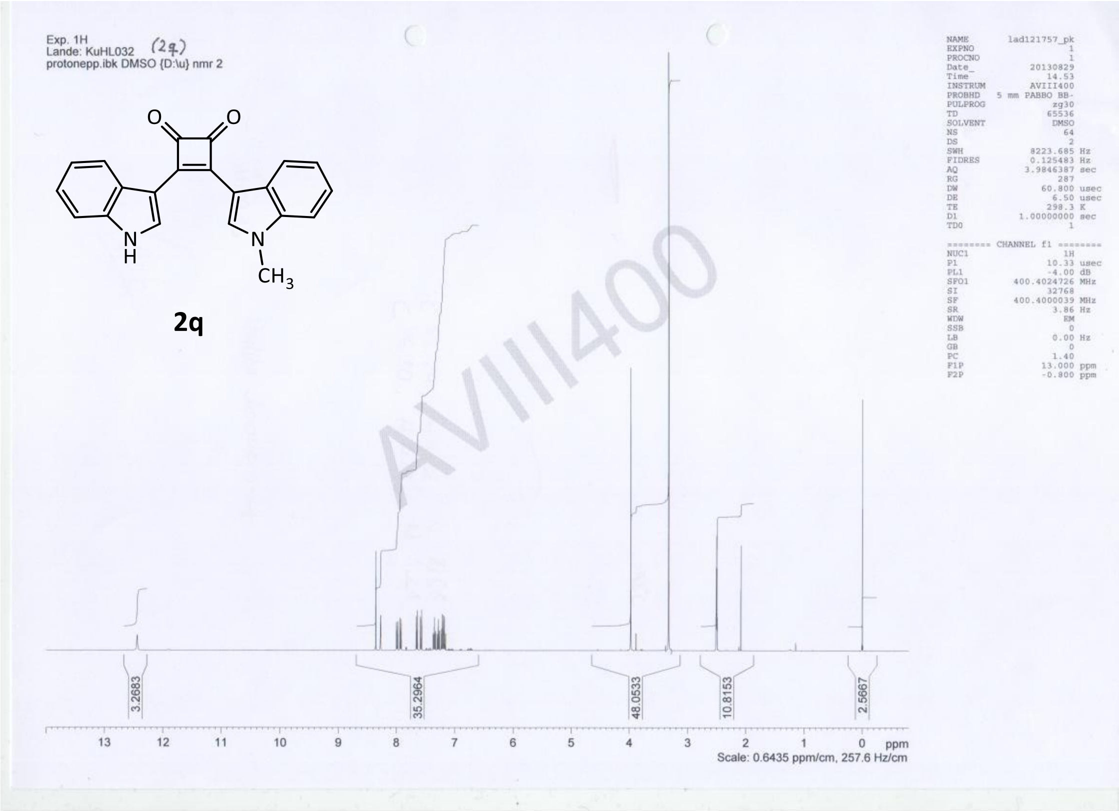
Exp. 1H
Lande: KuHL032
protonepp.ibk DMSO {D:\u} nmr 2
2q
3.2683
35.2964
48.0533
10.8153
2.5667
ppm
Scale: 0.6435 ppm/cm, 257.6 Hz/cm

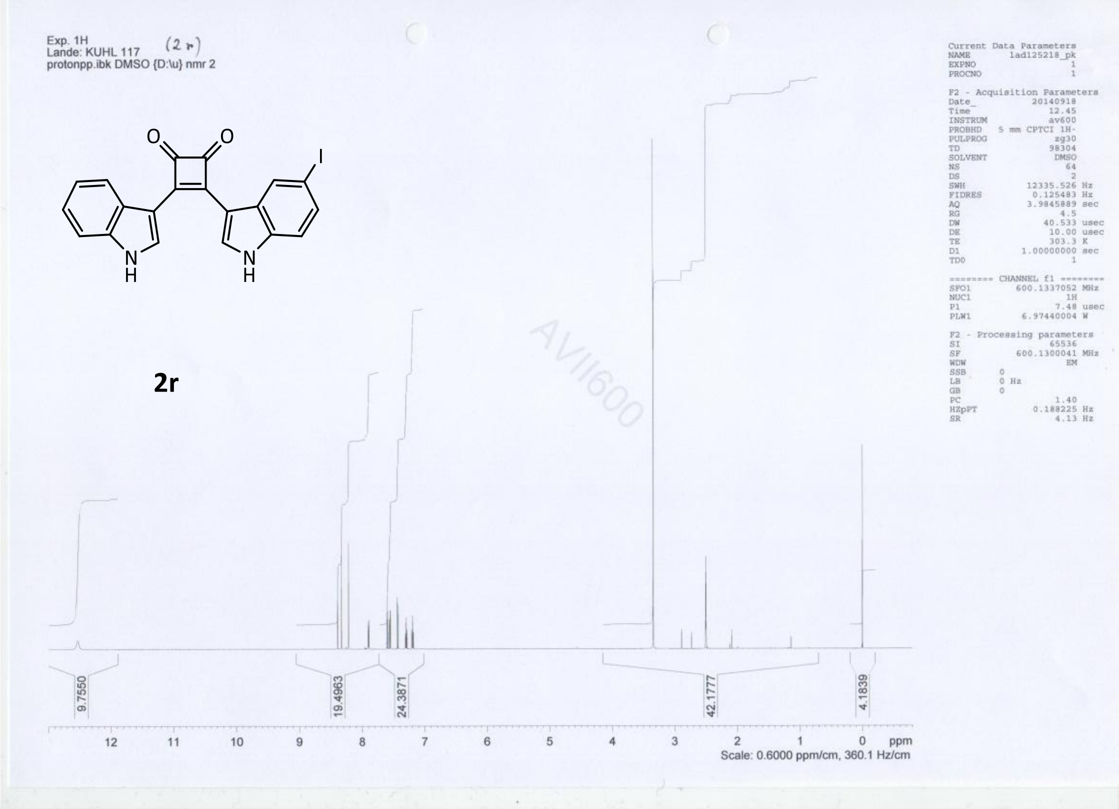

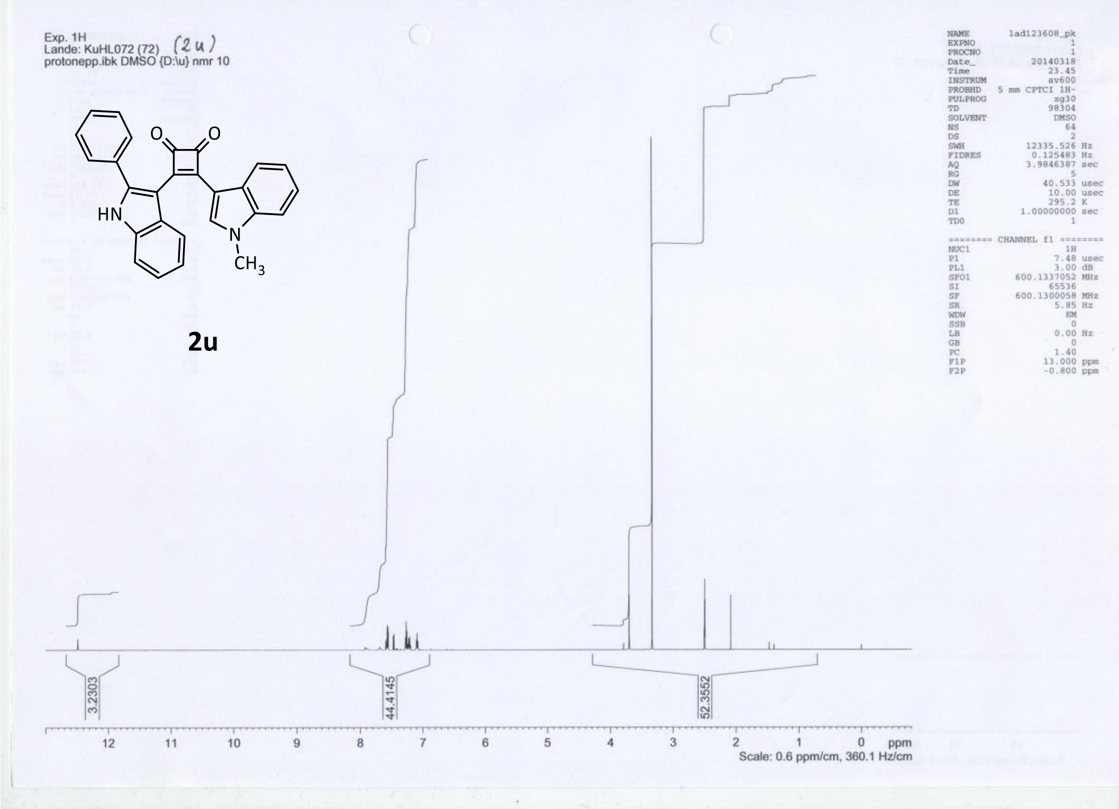

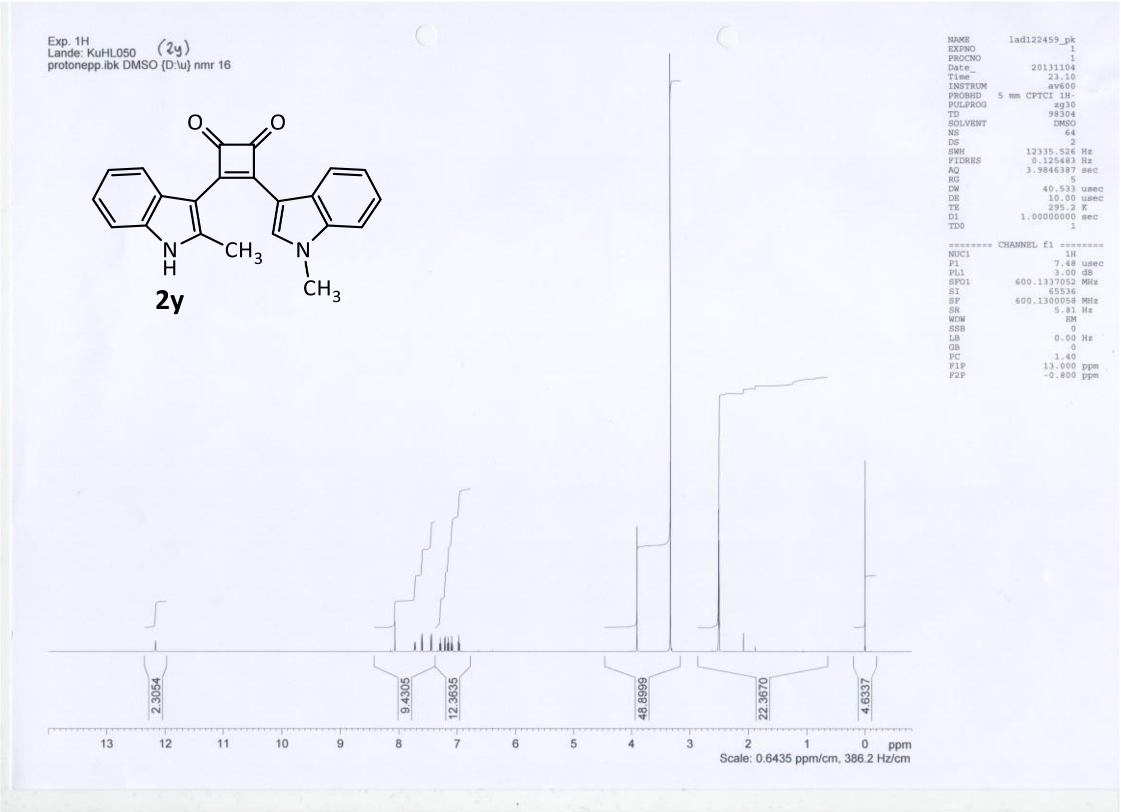
Exp. 1H
Lande: KuHL050 (2y)
protonepp.ibk DMSO {D:\u} nmr 16
2y
NAME lad122459_pk
EXPNO 1
PROCNO 1
Date_ 20131104
Time 23.10
INSTRUM av600
PROBHD 5 mm CPTCI 1H-
PULPROG zg30
TD 98304
SOLVENT DMSO
NS 64
DS 2
SWH 12335.526 Hz
FIDRES 0.125483 Hz
AQ 3.9846387 sec
RG 5
DW 40.533 usec
DE 10.00 usec
TE 295.2 K
D1 1.00000000 sec
TD0 1
======== CHANNEL f1 ========
NUC1 1H
P1 7.48 usec
PL1 3.00 dB
SFO1 600.1337052 MHz
SI 65536
SF 600.1300058 MHz
SR 5.81 Hz
WDW EM
SSB 0
LB 0.00 Hz
GB 0
PC 1.40
F1P 13.000 ppm
F2P -0.800 ppm
2.3054
9.4305
12.3635
48.8999
22.3670
4.6337
ppm
Scale: 0.6435 ppm/cm, 386.2 Hz/cm

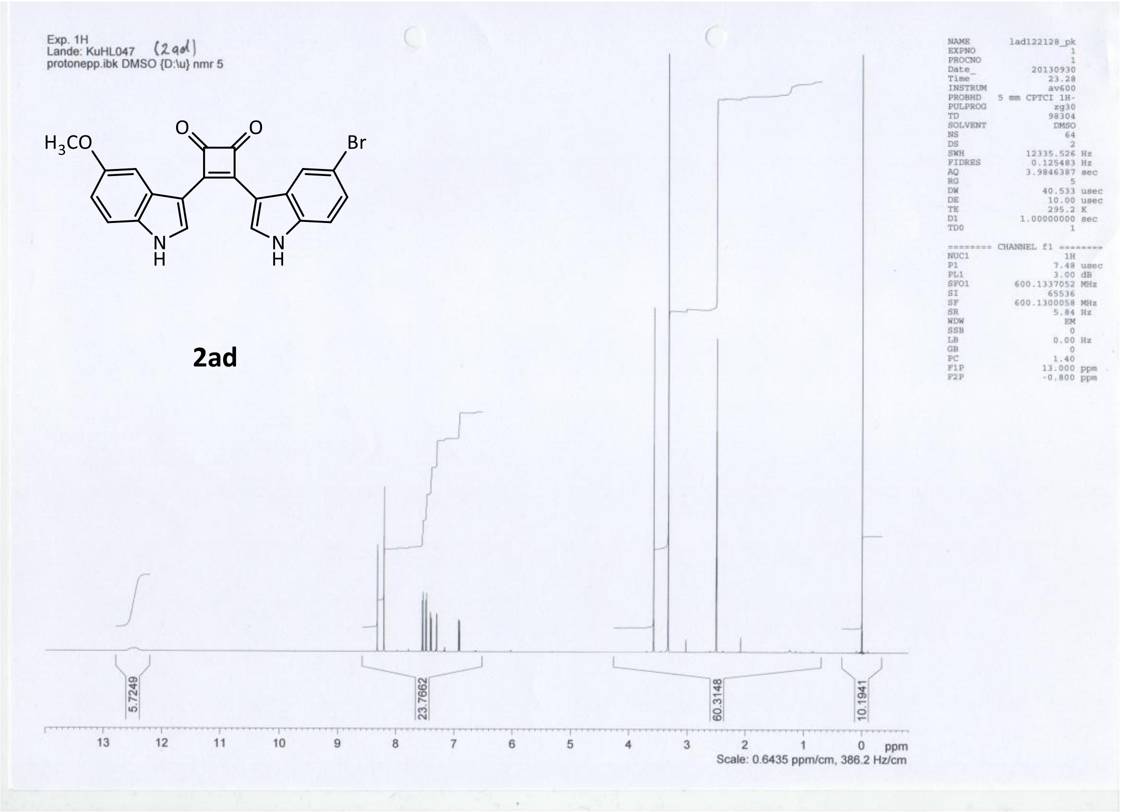
Exp. 1H
Lande: KuHL047 (2ad)
protonepp.ibk DMSO {D:\u} nmr 5
Br
2ad
NAME lad122128_pk
EXPNO 1
PROCNO 1
Date_ 20130930
Time 23.28
INSTRUM av600
PROBHD 5 mm CPTCI 1H-
PULPROG zg30
TD 98304
SOLVENT DMSO
NS 64
DS 2
SWH 12335.526 Hz
FIDRES 0.125483 Hz
AQ 3.9846387 sec
RG 5
DW 40.533 usec
DE 10.00 usec
TE 295.2 K
D1 1.00000000 sec
TD0 1
======== CHANNEL f1 ========
NUC1 1H
P1 7.48 usec
PL1 3.00 dB
SFO1 600.1337052 MHz
SI 65536
SF 600.1300058 MHz
SR 5.84 Hz
WDW EM
SSB 0
LB 0.00 Hz
GB 0
PC 1.40
F1P 13.000 ppm
F2P -0.800 ppm
5.7249
23.7662
60.3148
10.1941
ppm
Scale: 0.6435 ppm/cm, 386.2 Hz/cm

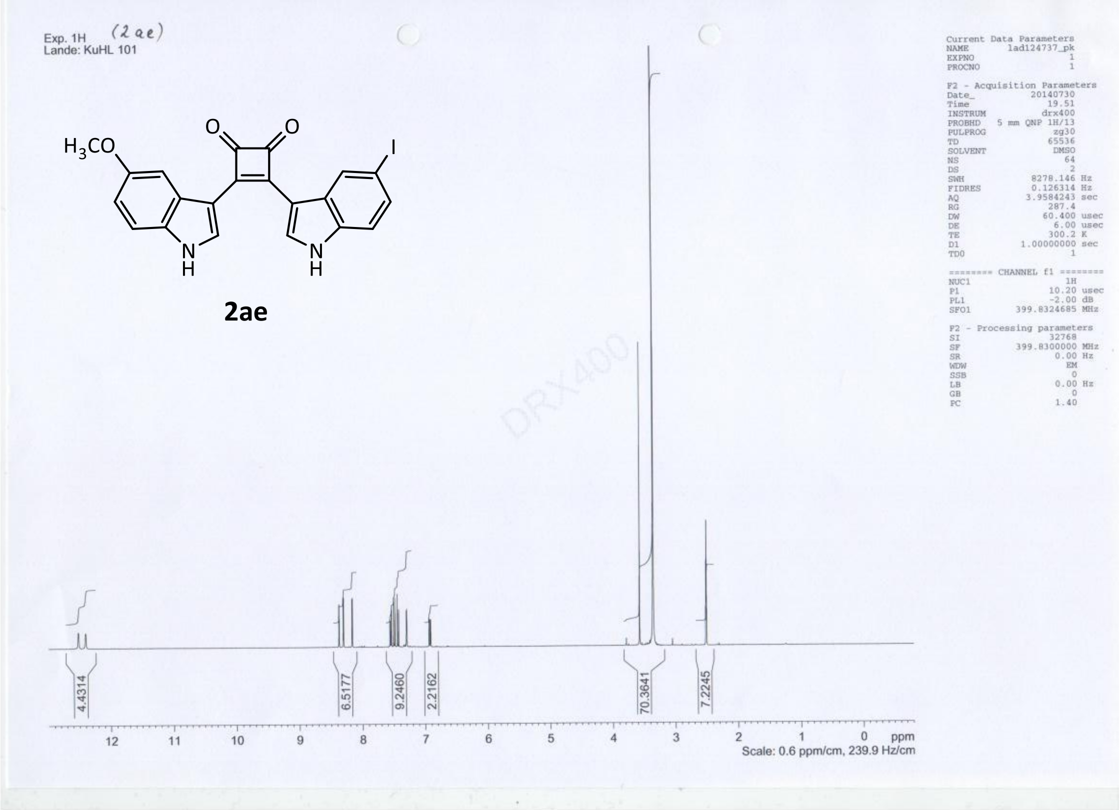
Exp. 1H (2ae)
Lande: KuHL 101
H3CO
2ae
ppm
Scale: 0.6 ppm/cm, 239.9 Hz/cm

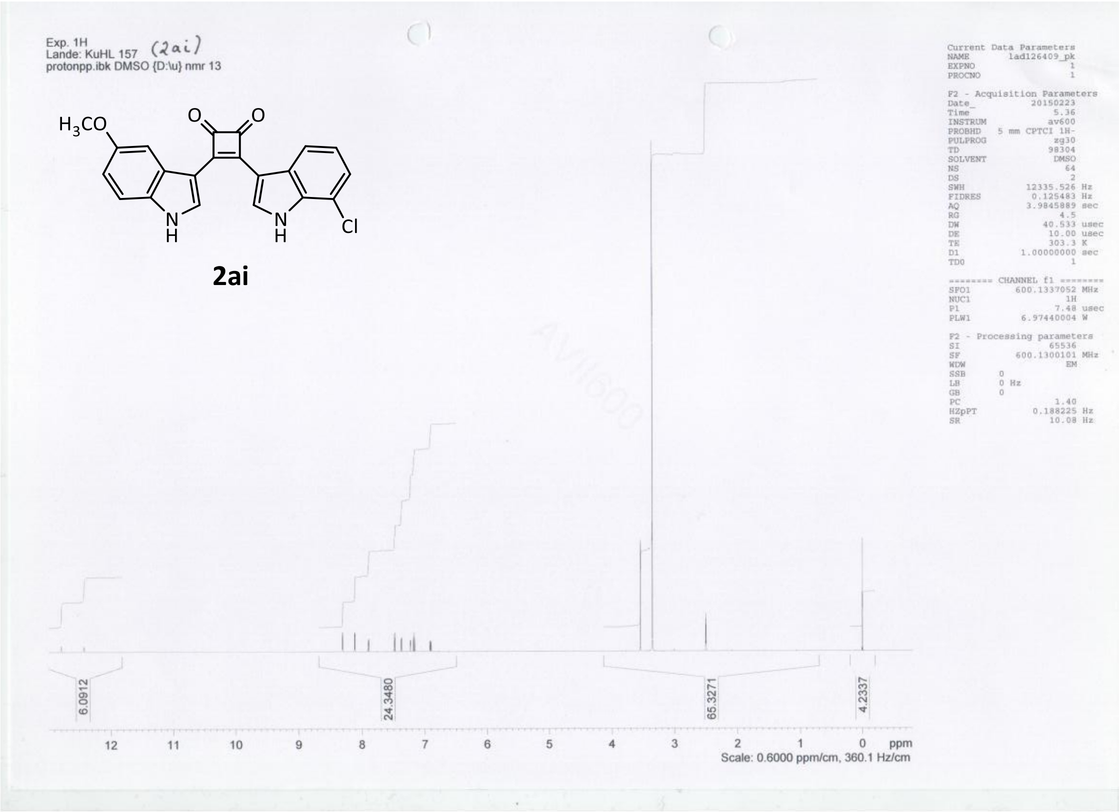
Exp. 1H
Lande: KuHL 157 (2ai)
protonpp.ibk DMSO {D:\u} nmr 13
H3CO
Cl
2ai
ppm
Scale: 0.6000 ppm/cm, 360.1 Hz/cm

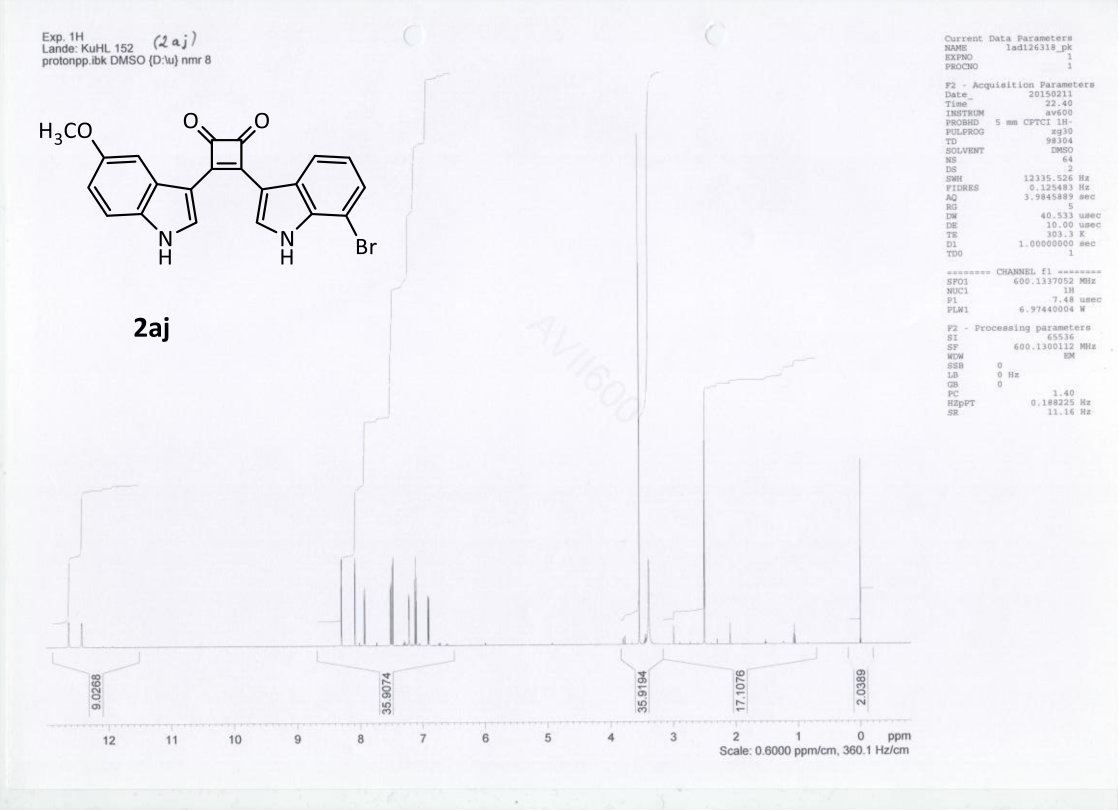
Exp. 1H
Lande: KuHL 152 (2aj)
protonpp.ibk DMSO {D:\u} nmr 8
H3CO
O
O
N
H
N
H
Br
2aj
Current Data Parameters
NAME 1ad126318_pk
EXPNO 1
PROCNO 1
F2 - Acquisition Parameters
Date_ 20150211
Time 22.40
INSTRUM av600
PROBHD 5 mm CPTCI 1H-
PULPROG zg30
TD 98304
SOLVENT DMSO
NS 64
DS 2
SWH 12335.526 Hz
FIDRES 0.125483 Hz
AQ 3.9845889 sec
RG 5
DW 40.533 usec
DE 10.00 usec
TE 303.3 K
D1 1.00000000 sec
TD0 1
======== CHANNEL f1 ========
SFO1 600.1337052 MHz
NUC1 1H
P1 7.48 usec
PLW1 6.97440004 W
F2 - Processing parameters
SI 65536
SF 600.1300112 MHz
WDW EM
SSB 0
LB 0 Hz
GB 0
PC 1.40
HZpPT 0.188225 Hz
SR 11.16 Hz
9.0268
35.9074
35.9194
17.1076
2.0389
12 11 10 9 8 7 6 5 4 3 2 1 0 ppm
Scale: 0.6000 ppm/cm, 360.1 Hz/cm

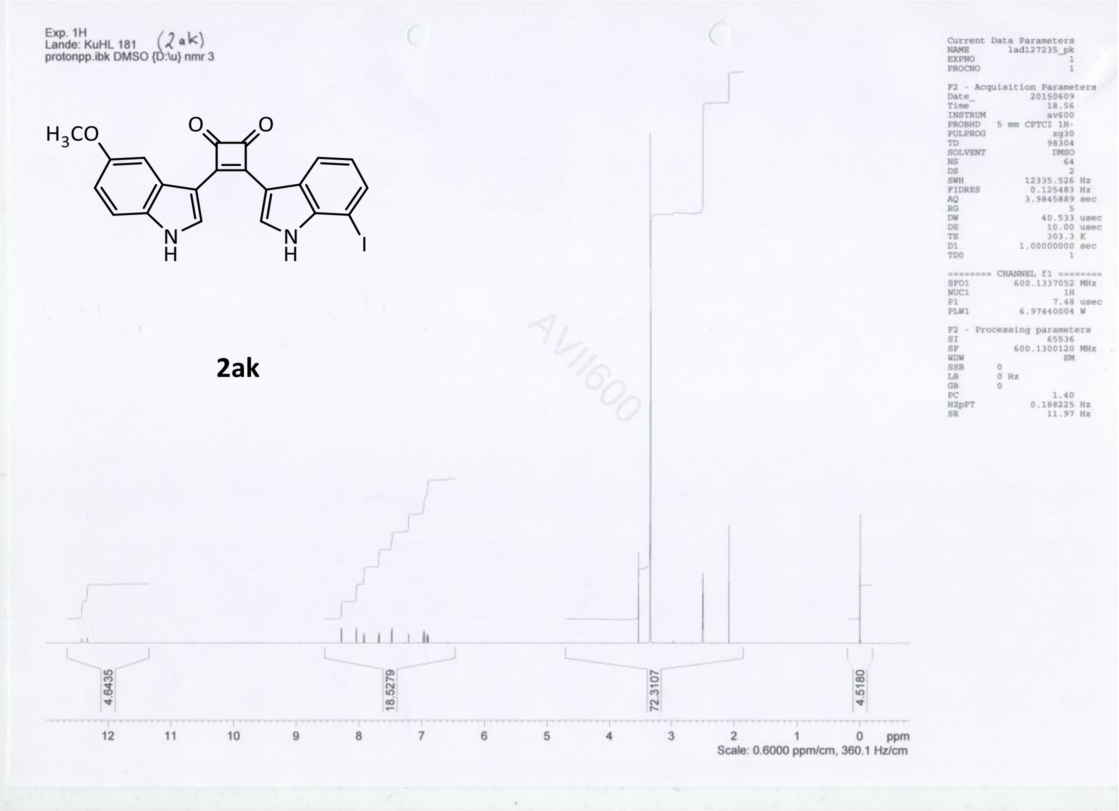
Exp. 1H
Lande: KuHL 181 (2ak)
protonpp.ibk DMSO {D:\u} nmr 3
H3CO
O
O
N
H
N
H
I
2ak
Current Data Parameters
NAME 1ad127235_pk
EXPNO 1
PROCNO 1
F2 - Acquisition Parameters
Date_ 20150609
Time 18.56
INSTRUM av600
PROBHD 5 mm CPTCI 1H-
PULPROG zg30
TD 98304
SOLVENT DMSO
NS 64
DS 2
SWH 12335.526 Hz
FIDRES 0.125483 Hz
AQ 3.9845889 sec
RG 5
DW 40.533 usec
DE 10.00 usec
TE 303.3 K
D1 1.00000000 sec
TD0 1
======== CHANNEL f1 ========
SFO1 600.1337052 MHz
NUC1 1H
P1 7.48 usec
PLW1 6.97440004 W
F2 - Processing parameters
SI 65536
SF 600.1300120 MHz
WDW EM
SSB 0
LB 0 Hz
GB 0
PC 1.40
HZpPT 0.188225 Hz
SR 11.97 Hz
4.6435
18.5279
72.3107
4.5180
12 11 10 9 8 7 6 5 4 3 2 1 0 ppm
Scale: 0.6000 ppm/cm, 360.1 Hz/cm

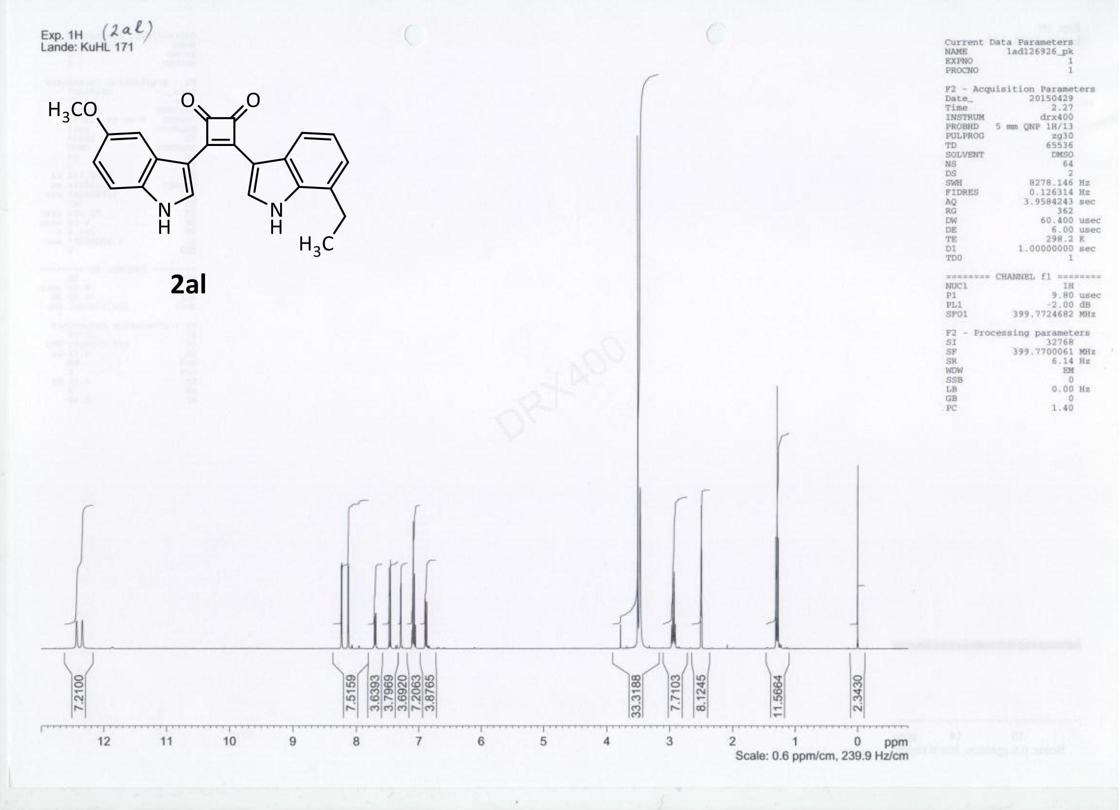
Exp. 1H (2al)
Lande: KuHL 171
2al
7.2100
7.5159
3.6393
3.7969
3.6920
7.2063
3.8765
33.3188
7.7103
8.1245
11.5664
2.3430
ppm
Scale: 0.6 ppm/cm, 239.9 Hz/cm
DRX400

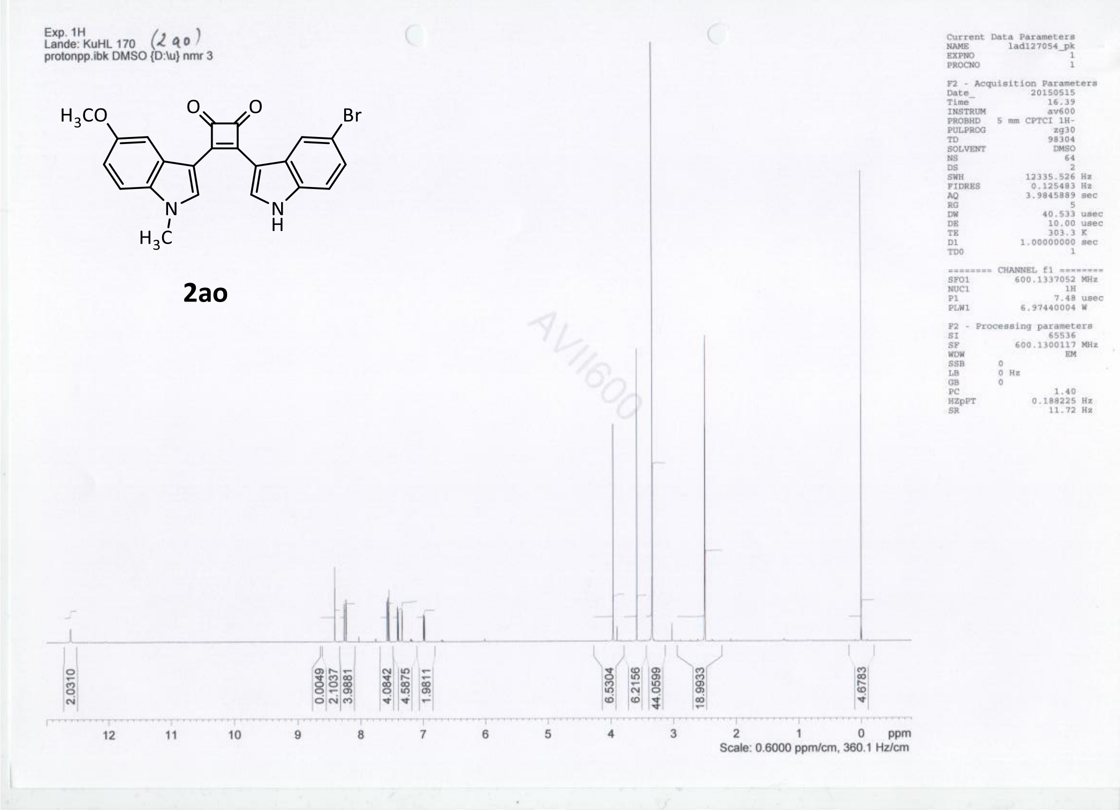
Exp. 1H
Lande: KuHL 170 (2ao)
protonpp.ibk DMSO {D:\u} nmr 3
2ao
2.0310
0.0049
2.1037
3.9881
4.0842
4.5875
1.9811
6.5304
6.2156
44.0599
18.9933
4.6783
ppm
Scale: 0.6000 ppm/cm, 360.1 Hz/cm
AVII600

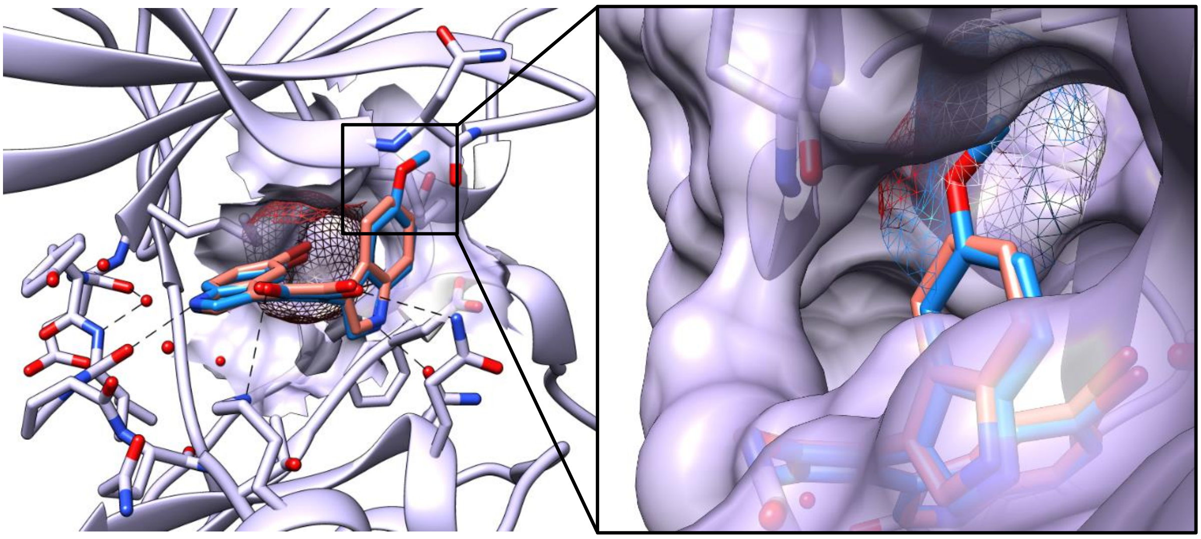

**Figure S1:** Suggested orientation generated by docking of **2b** (orange) and of **2ad** (blue) in the ATP binding pocket of a *Pf*GSK-3 homology model (light purple). While the nitrogen of the bromo-substituted indole ring acts as hydrogen bond donor to the hinge area, the bromo substituent occupies a hydrophobic back pocket. The second indole ring is orientated perpendicular. Detail shown on the right: The methoxy group of **2ad** fills a pocket at the roof of the ATP binding site. Although the results of these docking experiments could very well explain the antiplasmodial effect of the binsindoloylcyclobutenediones reported here, the enzymatic assays later showed that *Pf*GSK-3 is not the relevant biological target of this class of compounds.
